# Synthesis of a Naphthalocyanine-Like Dye: The First Report on Zn(II)-1,6-methano[10]annulenecyanine

**DOI:** 10.3390/molecules25092164

**Published:** 2020-05-05

**Authors:** Nicholas Roberto da Silva Gobo, Timothy John Brocksom, Kleber Thiago de Oliveira

**Affiliations:** 1 Departamento de Química, Universidade Federal de São Carlos, São Carlos CEP 13.565-905, São Paulo 01000-000, Brazil; nicholas.gobo@accert.com (N.R.d.S.G.); brocksom@terra.com.br (T.J.B.); 2 ACCERT Chemistry and Biotechnology, São Carlos CEP 13.574-033, São Paulo 01000-000, Brazil

**Keywords:** annulene, naphthalocyanine, phthalocyanine, NIR chromophores and dyes

## Abstract

The synthesis of the new dye 1,6-methano[10]annulenecyanine is described. For this purpose, the 3,4-dicyano-1,6-methano[10]annulene and 3,4-carboxyimide-1,6-methano[10]annulene buildings blocks were synthesized in six to eight steps. In both cases, these building blocks were then cyclotetramerized to furnish a new Zn(II)-1,6-methano[10]annulenecyanine which presents a strong red-shifted absorption band at 800 nm and high solubility in common organic solvents.

## 1. Introduction

Near-infrared (NIR) dyes have received increasing attention due to their versatile applications in materials science [1,2,3,4,5], medical applications [6,7,8,9,10,11,12,13,14], catalysis and other advanced technology devices [12,15,16,17]. In this context, phthalocyanines and naphthalocyanines are very robust dyes with relevant photophysical properties and must be highlighted [18,19,20,21,22,23].

These classes of compounds are not naturally occurring, unlike their analogues porphyrins, chlorins and bacteriochlorins [24,25]. Phthalocyanines were accidentally discovered at the beginning of the last century and only systematically studied in the 1930s by Linstead and Robertson, who finally established their structure and a general synthetic methodology [26,27,28,29,30,31]. Since then their thermal stability, low solubility and intense color took up attention. Over the past few decades many improved synthetic approaches for phthalocyanine and also naphthalocyanine derivatives have been established, with a relevant molecular diversity which allows the modulation of both chemical and photochemical properties of these dyes [26]. In general, the classic synthetic approach to obtain phthalocyanines and naphthalocyanines is the cyclotetramerization of their building blocks phthalonitriles and naphthalonitriles, respectively [26,27,28,29,30,31]. Depending on the building block substitution pattern, it is possible to modulate the photophysical properties of the phthalocyanine/naphthalocyanine of interest, fine-tuning the dye for the desired application. The literature furnishes a vast number of interesting and creative molecules using this approach [26,27,28,29,30,31].

**Figure 1:**
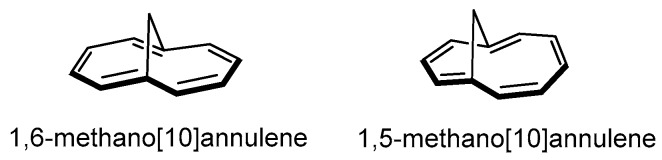
Vogel’s bridged annulenes.

Seeking for new NIR building blocks our group decided to explore a class of molecules called 1,x-methano[10]annulenes, especially the 1,6-methano[10]annulene (Figure 1) [32]. These compounds have fascinated a number of scientists since Emanuel Vogel’s discovery in 1964 [33]. Vogel and coworkers achieved the required planarity of fully conjugated 10-membered carbon rings by inserting a carbon bridge between C-1 and C-6 (Figure 1), thus furnishing 1,6-methano[10]annulene as an example of an aromatic cyclodecapentaene ring. Up to now, this class of compounds was extensively studied in synthetic, theoretical and biological areas [34]. However, to our knowledge, suitable derivatives of these compounds have not been described in the literature as building blocks for phthalocyanine and naphthalocyanine-like compounds.

**Scheme 1:**
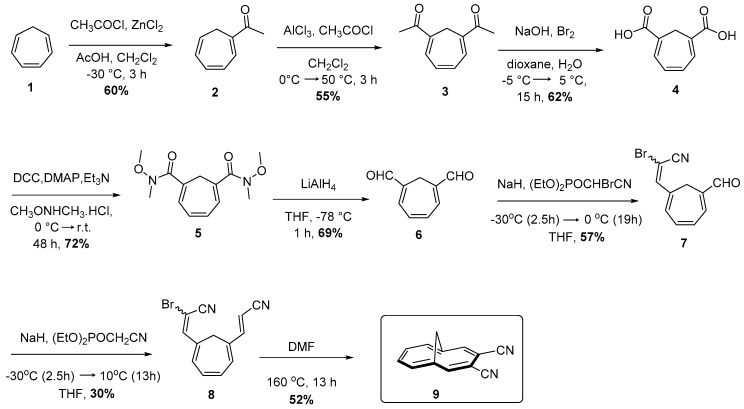
Synthetic approach to the annulenenitrile **9** building block.

**Scheme 2:**
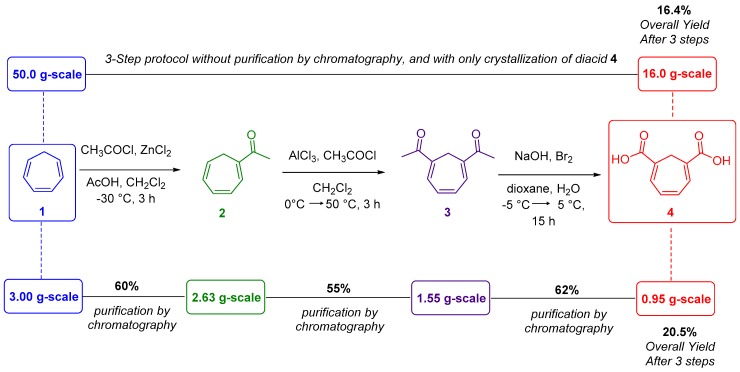
Scale-up of the intermediate **4**.

**Scheme 3:**
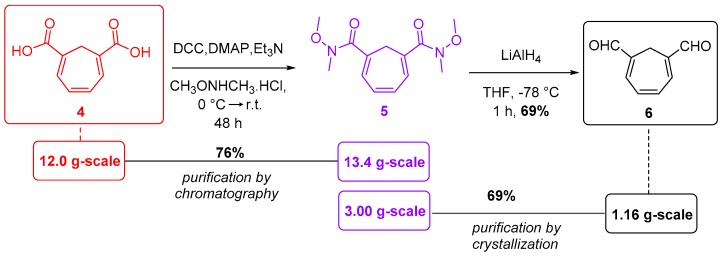
Scale-up of the intermediate **6**.

**Scheme 4:**
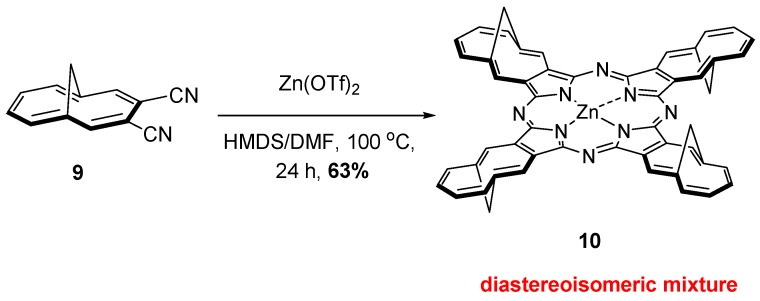
Synthesis of annulenecyanine **10**.

**Scheme 5:**
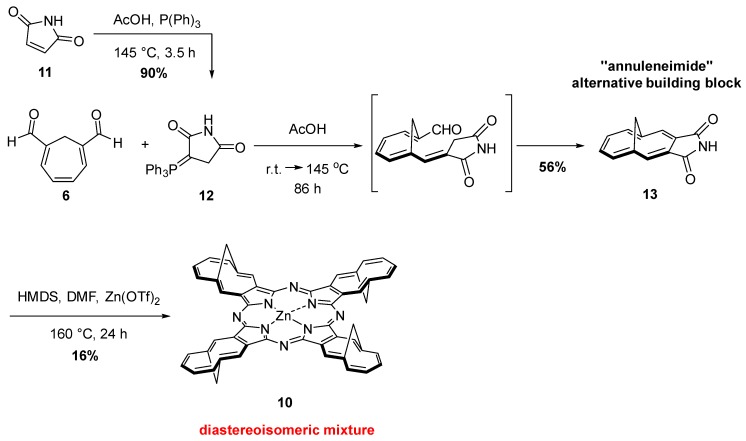
Alternative approach to the synthesis of **10**.

Herein, we describe two approaches for the synthesis of a new naphthalocyanine-like compound which we name as Zn(II)-1,6-methano[10]annulenecyanine (**10**), using annulenenitrile **9** and annulenemaleimide **13** as building blocks (Scheme 1, Scheme 2, Scheme 3, Scheme 4 and Scheme 5).

## 2. Results and Discussion

### Synthesis

The synthesis of the building block 3,4-dicyano-1,6-methano[10]annulene (**9**) was carried out as shown in Scheme 1 [35]. First, cycloheptatriene (**1**) was mono-acetylated at low temperature by a Friedel–Crafts reaction furnishing compound **2** in 60% yield. Then, the resulting ketone **2** was acetylated again, yielding the 1,3-diketone **3** in 55% yield. The diacid **4** was obtained in 62% yield by submitting compound **3** to the haloform reaction. Subsequently, the *bis*-Weinreb amide **5** was prepared using *N-O*-dimethylhydroxylamine hydrochloride (72% yield), and the dialdehyde **6** obtained after reduction with LiAlH_4_ at −78 °C (69% yield). The next steps required selective olefinations of dialdehyde **6** with two different olefination agents (Scheme 1). First, compound **7** was obtained in 57% yield by a chemoselective Horner–Wadsworth–Emmons olefination of dialdehyde **6**. The literature describes an olefination under phase-transfer catalysis conditions that could give compound **8**, but, in our hands only HBr elimination was observed [36]. Therefore, a modified procedure to reach **8** was tested and furnished the desired compound in 30% yield. Attempts to optimize this yield were tested, but unsuccessfully. The annulenenitrile **9** was obtained by a one-pot electrocyclization followed by HBr elimination/aromatization (52% yield) (Scheme 1). The overall yield for the eight steps is 0.90%.

As the synthesis of annulenenitrile **9** involves eight steps we decided to scale-up some reactions in order to obtain gram-scale amounts of intermediates like **4** (Scheme 2) and **6** (Scheme 3). First, we considered the linear synthesis of **4** (Scheme 2) starting from 3.00 g of **1** and isolating each intermediate by column chromatography, thus obtaining **4** in a 0.95 g-scale (20.5% overall yield after three steps). Attempts to synthesize **4** from **1** with no intermediate purification by chromatography were performed starting from 50.0 g of cycloheptatriene (**1**) and, to our delight, the diacid **4** was obtained in 16.4% overall yield in a very short time. Additionally, only a simple final crystallization yielded 16.0 g of **4**.

Next, we tested two additional optimizations. First, the synthesis of the bis-Weinreb amide **5** was carried out starting from 12.0 g of **4**, thus obtaining **5** in 76% (13.4 g - Scheme 3). Furthermore, the scaled-up reduction of 3.0 g of **5** was successfully achieved, obtaining the di-aldehyde **6** in 1.16 g (69% yield). It is important to mention that the reduction of Weinreb amides requires a finely temperature-controlled reaction, with difficult scalabilities in batch conditions.

After achieving improved conditions for obtaining annulenonitrile **9** in practical amounts, we tested the first cyclotetramerization condition for obtaining the desired Zn(II)-1,6-methano[10]annulenecyanine (**10**) (Scheme 4) using *N,N*-dimethylethanolamine (DMAE) and zinc acetate dihydrate under thermal conditions. However, the new dye **10** was obtained in only trace amounts as determined by UV–Vis spectroscopy [5,12]. Fortunately, using zinc triflate in hexamethyldisilazane (HMDS) and *N,N*-dimethylformamide (DMF) at 100 °C for 24 h yielded **10** in 63% yield after purification by chromatography [37].

**Figure 2:**
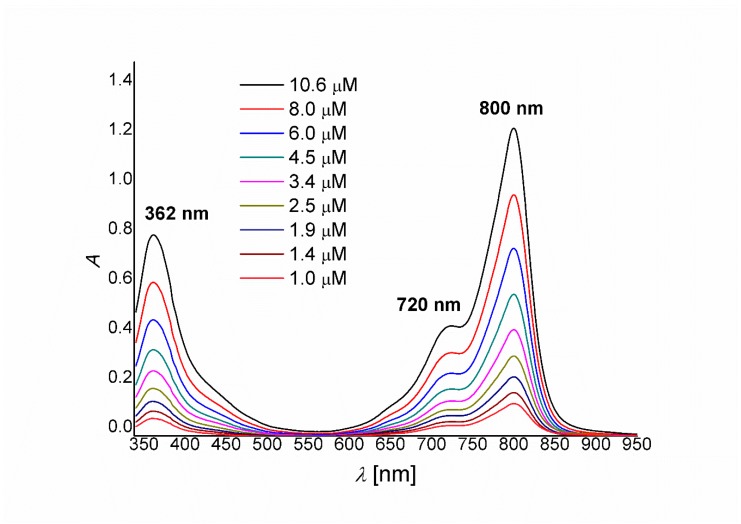
UV–Vis spectra of annulenocyanine **10** in DMF.

The compound **10** (diastereoisomeric mixture) was initially characterized by UV–Vis spectroscopy showing characteristic naphthalocyanine-like absorption bands at 362 and 800 nm (Figure 2). Additionally, solutions of **10** in DMF indicated good solubility up to 10 µM and apparent non-aggregation in solution, which are very important properties of highly conjugated naphthalocyanine-like dyes. This preliminary observation on non-aggregation was obtained only considering UV–Vis analysis at concentrations up to 10.6 mM in DMF, since linear increases of absorbance are achieved with increased concentrations (see Figures S56–S58 in the supporting information). However, since a mixture of diastereoisomers is present in solution, further studies are necessary to conclude about the aggregation of **10**.

**Figure 3:**
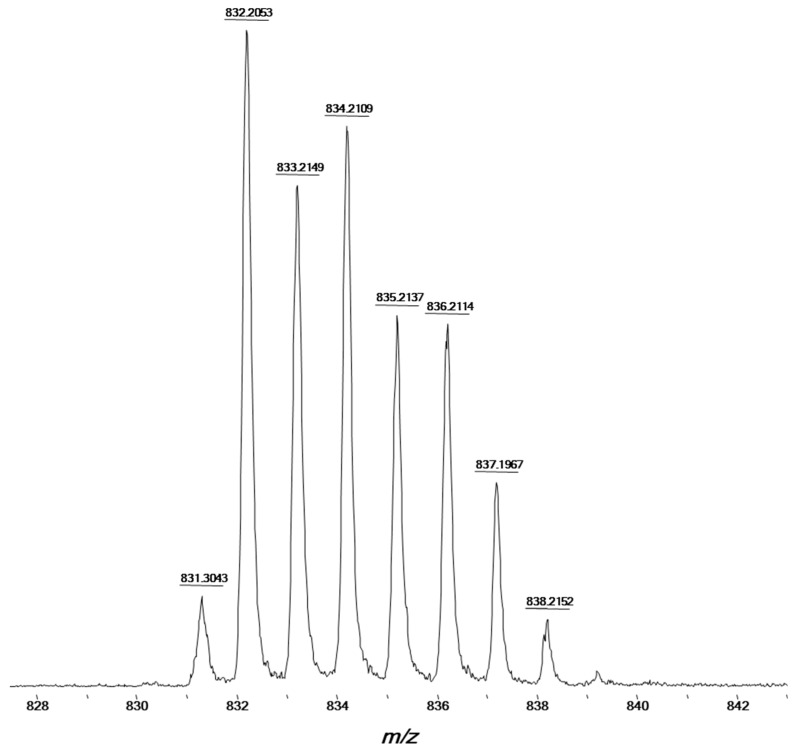
HRMS-MALDI-TOF of **10**.

The HRMS-MALDI-TOF analysis of **10** was also consistent with a characteristic isotopic pattern for metallated naphthalocyanine-like compounds and the experimental *m/z* for [M]^+^ correspond to the expected for this dye (Figure 3 - Calc. for [M]^+^, C_52_H_32_N_8_Zn^+^, 832.2041, found: 832.2053).

Attempts to perform the separation of the diastereoisomers of **10** were tested by HPLC, but unsuccessfully, making the NMR characterizations even more difficult. We tested different deuterated solvents (THF-*d*_8_, DMF-*d*_7_, acetone-*d*_6_ and CDCl_3_) but in all cases aggregation and complex mixtures of signals were observed making it difficult to complete assignments. Despite this, it was possible to identify in the ^1^H-NMR signals at the aromatic region 7.50–8.30 ppm corresponding to the hydrogens of the macrocycle periphery, and multiplets at 0.82–0.92 ppm which are consistent with the expected for CH_2_-bridged [10]annulenes (signals at upfield region—see examples of the precursors **9** and **13** in the supporting information). It is important to comment that difficulties for characterizations by NMR of naphthalocyanine and phthalocyanine derivatives are well-known in the literature, and the evidence presented here is consistent with the expected for the new dye **10**.

We then decided to find improvements for our original linear synthetic approach (Scheme 1, Scheme 2, Scheme 3 and Scheme 4, 0.57% overall yield, nine steps).

Our alternative approach for the synthesis of **10** uses the precursor annulenemaleimide **13** (Scheme 5). For the synthesis of **13** we decided to use the same di-aldehyde intermediate **6**, which in a one-pot Witting olefination with the ylide **12** and a 10π electrocyclization and dehydration yielded **13** in 56% yield [38,39]. Subsequently, compound **13** was submitted to the same cyclotetramerization conditions and furnished **10** in 16% yield. Attempts to optimize this last step were carried out, but no better results were achieved. Overall, the diastereomeric mixture of dye **10** is now obtained in seven steps and 0.91% overall yield.

**Figure 4:**
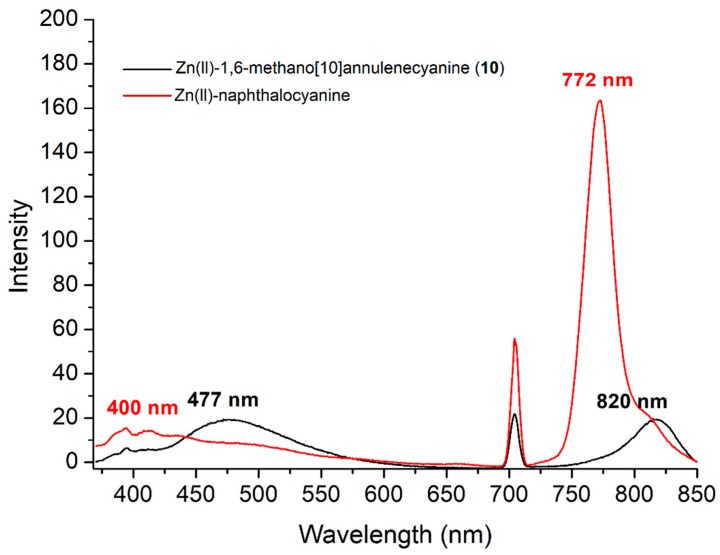
Emission spectra of compound **10** in comparison with standard Zn(II)-naphthalocyanine (**ZnNc**), both excited at 350 nm and analyzed in DMF.

The fluorescence quantum yield (Φ_F_) of **10** was determined using as standard the corresponding zinc naphthalocyanine by exiting both at 350 nm (Figure 4). We found the Φ_F_ for **10** as being 0.01 with an emission band at 820 nm, a common value for organic compounds which present aggregation and dissipate energy in solution by non-radiant processes [40,41]. Therefore, the fluorescence technique reinforces that comprehensive studies on aggregation should be conducted in the future, in order to understand better the self-association properties of **10** (being a mixture of diastereoisomers) in solutions. These additional studies will be essential for using this new dye as a photosensitizer and for NIR applications.

Overall, this first report on the dye **10** opens up many possibilities for the synthesis of hybrid systems with modulated chemical, photochemical and photophysical properties.

## 3. Materials and Methods

All reagents and starting materials and solvents were purchased from commercial sources and used as received or purified when necessary. Some reactions were carried out under an argon atmosphere as specified in the experimental procedures (see the supporting information). For NMR spectra (performed in CDCl_3_ or in DMSO-*d*_6_ solutions) tetramethylsilane was used as internal reference for ^1^H (0 ppm), and C-D coupling signal as internal reference for ^13^C (CDCl_3_—77.0 ppm and DMSO—39.5 ppm).

Flash chromatography was carried out using silica gel (230–400 mesh). Infrared spectra were registered using KBr cells for liquid (films) and KBr pellets for solids. Fluorescence emission spectra were recorded using 1 cm optical length cuvettes at 25 °C and *N,N*-dimethylformamide as solvent. Analytical TLC was carried out on precoated aluminum sheets with silica gel (0.2 mm thick). UV–Vis analyses were performed using a double beam spectrometer with 0.1 nm of resolution. High resolution mass spectrometry was carried out on a MALDI-TOF for compound **10**, and ESI-TOF for compounds **2**, **3**, **4**, **5**, **6**, **7**, **8**, **9** and **13**.

Experimental details, spectroscopic and spectrometric data of all key compounds are available online in the supporting information.

## 4. Experimental Procedures

*1-(cyclohepta-1,3,5-trien-1-yl)ethan-1-one* (**2**): To a suspension of zinc (II) chloride (36.0 g, 264 mmol) in 23 mL of dichloromethane at −30 °C, under an argon atmosphere, 7.00 mL (7.73 g; 98.4 mmol) of acetyl chloride and 5.60 mL (5.87 g; 97.8 mmol) of acetic acid were added. Then, 3.40 mL (3.00 g; 32.4 mmol) of cycloheptatriene (**1**) were added dropwise (during *ca* 10 min). The reaction mixture was stirred for 3 h at −30 °C. The reaction mixture was quenched with 50 g of ice and neutralized with sodium bicarbonate. The organic layer was washed with brine (50 mL) and dried over anhydrous sodium sulphate. The solvent was removed under vacuum and the organic residue was purified by chromatography on silica gel using CH_2_Cl_2_:hexano (5:5) → (6:4) as eluent, furnishing a yellow oil as product. **Yield:** 60% (2.63 g; 19.6 mmol). **^1^H-NMR** (CDCl_3_, 400.15 MHz), *δ* ppm): 2.38 (s, 3H); 2.65 (d, 2H, *J*_1_ = 7,0 Hz); 5.57 (dt, 1H, *J*_1_ = 9.0 Hz, *J*_2_ = 7.0 Hz); 6.27 (dd, 1H, *J*_1_ = 9.3 Hz, *J*_2_ = 5.7 Hz); 6.70 (ddt, 1H, *J*_1_ = 11.2 Hz, *J*_2_ = 6.0 Hz, *J*_3_ = 0.8 Hz); 6.86 (dd, 1H, *J*_1_ = 11.2 Hz, *J*_2_ = 5.7 Hz); 7.09 (d, 1H, *J* = 6.0 Hz). **^13^C-NMR** (CDCl_3_, 100.0 MHz) *δ* (ppm): 25.4; 26.3; 125.9; 127.2; 129.3; 131.8; 133.1; 136.1; 197.6. **IR:** *ν*_max_: (KBr): 3308 cm^−1^; 3024 cm^−1^; 2928 cm^−1^; 2886 cm^−1^; 2839 cm^−1^; 1732 cm^−1^; 1666 cm^−1^; 1605 cm^−1^; 1528 cm^−1^; 1431 cm^−1^; 1385 cm^−1^; 1364 cm^−1^; 1209 cm^−1^; 1211 cm^−1^; 1182 cm^−1^; 976 cm^−1^; 789 cm^−1^; 758 cm^−1^; 708 cm^−1^. **HRMS** (ESI-TOF): **calc. for** [M+H]^+^, C_9_H_11_O^+^, 135.0804; **found:** 135.0806.

*1,1’-(cyclohepta-3,5,7-triene-1,3-diyl)diethanone* (**3**): In a suspension containing 1.49 g(11.2 mmol) of aluminium chloride and 6.00 mL of dry dichloromethane at 0 °C, 0.79 mL (870 mg; 11.2 mmol) of acetyl chloride was added under an argon atmosphere. After 5 min, 500 mg (3.73 mmol) of **2** was added. Then, the cooling bath was removed and the reaction mixture heated to 55 °C for 3 h. After this period, the reaction mixture was cooled to 0 °C and 5 mL of water at 5 °C was added. The reaction mixture was neutralized with sodium bicarbonate solution and filtered in a sintered funnel. The filtrate was washed with ethyl acetate (5 × 50 mL). The solvent was removed under vacuum and the organic residue (a brown oil) was purified by column chromatography on silica gel using toluene: ethyl acetate (95:5) as eluent, furnishing a yellow oil as product. **Yield:** 50% (333 mg; 1.89 mmol). **^1^H-NMR** (CDCl_3_, 400.15 MHz), *δ* (ppm): 2.24(s, 6H); 2.99(s, 2H); 6.98 (dd, 2H, *J*_1_ = 4.0 Hz, *J*_2_ = 3.0 Hz); 7.17 (dd, 2H, *J*_1_ = 3.9 Hz, *J*_2_ = 3.0 Hz). **^13^C-NMR** (CDCl_3_, 100.0 MHz) *δ* (ppm): 24.0; 26.7; 132.6; 134.1; 134.4; 197.1. **HRMS** (ESI-TOF): calc for [M+H]^+^, C_11_H_13_O_2_^+^, 177.0910; **found:** 177.0912. **Note:** Compound **3** is unstable under light and room temperature exposure. It must be stored at low temperature and protected from light.

*cyclohepta-3,5,7-triene-1,3-dicarboxylic acid* (**4**): To 150 mL aqueous sodium hydroxide solution (1.2 mol/L), 14 mL of 1,4-dioxane was added. Then this mixture was cooled to −5 °C and 2.60 mL of bromine (8.06 g; 50.7 mmol) was added. After 5 min, a solution containing 1.5 g (8.5 mmol) of compound **3** in 14 mL 1,4-dioxane was added dropwise (*ca* 10 min). Then, the reaction mixture was allowed to reach 5 °C and stirred for 15 h. After that, 100 mL of a solution 0.3 mol/L of sodium metabisulfite was added and stirred for 1h. The pH of the medium was adjusted to 3–4 with 10% solution of hydrochloric acid. The product was obtained as a precipitate which was filtered and washed with water (100 mL), ethanol (50 mL) and ethyl ether (50 mL) and dried. **Yield:** 62% (949 mg; 5.26 mmol). **^1^H-NMR** (DMSO, 400.15 MHz), *δ* (ppm): 2.88 (s, 2H); 6.91 (t, 2H, *J* = 2.8 Hz); 7.19 (t, 2H, *J* = 2.8 Hz); 12.60 (s, 2H). **^13^C-NMR** (DMSO, 100.0 MHz) *δ* (ppm): 25.1; 125.7; 132.4; 133.6; 166.5. **IR:** *ν*_max_: (KBr pellet): 744 cm^−1^; 905 cm^−1^; 935 cm^−1^; 1227 cm^−1^; 1421 cm^−1^; 1440 cm^−1^; 1609 cm^−1^; 1678 cm^−1^; 2619 cm^−1^; 2928 cm^−1^; 2974 cm^−1^; 3004 cm^−1^; 3474 cm^−1^. **HRMS** (ESI-TOF): calc for [M+Na]^+^, C_9_H_8_O_4_Na^+^, 203.0315; found: 203.0317.

*N^1^,N^3^-dimethoxy-N^1^,N^3^-dimethylcyclohepta-3,5,7-triene-1,3-dicarboxamide* (**5**): To a suspension containing 900 mg (5.00 mmol) of diacid **4** in 60 mL of dichloromethane at 0 °C and under an argon atmosphere was added 1.65 g (16.9 mmol) of *N-O*-dimethylhydroxylamine hydrochloride, 390.0 mg (3.18 mmol) of *N*,*N*-Dimethyl-4-aminopyridine, 2.70 g (13.1 mmol) of *N*, *N’*-Dicyclohexylcarbodiimide and 3.60 mL (2.61 g; 25.6 mmol) of triethylamine. The reaction mixture was stirred for 1 h at 0 °C, then 48 h at room temperature. After this period, the solvent was removed under vacuum and the residue partially dissolved with 150 mL of ethyl acetate/pentane mixture (1:1) and filtered off. The organic phase was concentrated under vacuum and the residue was purified by column chromatography on silica gel using dichloromethane/ethyl acetate (8:2) → (6:4) as eluent furnishing a yellow oil as product. **Yield:** 72% (960 mg; 3.60 mmol). **^1^H-NMR** (CDCl_3_, 400.15 MHz), *δ* (ppm): 2.84 (s, 2H); 3.18 (s, 6H); 3.53 (s, 6H); 6.62–6.63 (m, 4H). **^13^C-NMR** (CDCl_3_, 100.0 MHz) *δ* (ppm): 30.7; 33.4; 61.2; 129.4; 130.2; 132.0; 169.5. **HRMS** (ESI-TOF): calc. for [M+H]^+^, C_9_H_9_O_2_^+^, 267.1339; **found:** 267.1341. **IR:** *ν*_max_: (KBr): 750 cm^−1^; 763 cm^−1^; 973 cm^−1^; 1275 cm^−1^; 1380 cm^−1^; 1610 cm^−1^; 1634 cm^−1^; 2934 cm^−1^; 2963 cm^−1^.

*Cyclohepta-3,5,7-triene-1,3-dicarbaldehyde* (**6**): To a suspension containing 2.73 g (71.9 mmol) of lithium aluminum hydride and 180 mL of anhydrous tetrahydrofuran at −78 °C under an argon atmosphere, amide **5** (3.00 g; 11.3 mmol) previously dissolved in 80 mL of anhydrous tetrahydrofuran was added dropwise (*ca* 80 min). The reaction was stirred for 20 min, 100 mL of 0.50 mM aqueous solution of potassium bisulphate was added and the reaction allowed to reach 0 °C. Then 200 mL of 5% aqueous solution (wt/v) of citric acid was added and the reaction mixture was extracted with dichloromethane (3 × 100 mL). The organic layer was dried over anhydrous sodium sulphate, filtered and the solvent removed under vacuum (thermal bath at 20 °C). The aldehyde **6** was crystalized in an ethyl acetate/pentane mixture, furnishing a yellow-pale solid (999 mg; 6.73 mmol). The mother liquor was purified by chromatography on silica gel using CH_2_Cl_2_ → CH_2_Cl_2_:AcOEt (9:1) furnishing 155.7 mg (1.05 mmol). **Total** **Yield:** 69% (1.15 g, 7.78 mmol). **^1^H-NMR** (CDCl_3_, 400.15 MHz), *δ* (ppm): 3.08 (s, 2H); 7.00 (d, *J* = 2.7 Hz); 7.04 (dd, 2H, *J*_1_ = 3.8 Hz, *J*_2_ = 2.7 Hz); 9.54 (s, 2H). **^13^C-NMR** (CDCl_3_, 100.0 MHz) *δ* (ppm): 19.0; 134.2; 136.2; 141.3; 191.0. **IR:** *ν*_max_: (KBr pellet): 1059 cm^−1^; 1233 cm^−1^; 1431 cm^−1^; 1609 cm^−1^; 1682 cm^−1^; 2777 cm^−1^, 2849 cm^−1^, 2920 cm^−1^, 3020 cm^−1^, 3308 cm^−1^, 3449 cm^−1^. **HRMS** (ESI-TOF): **calc for** [M+H]^+^, C_9_H_9_O_2_^+^, 149.0597; **found:** 149.0599. **mp:** 121–123 °C.

*2-bromo-3-(6-formylcyclohepta-1,3,5-trien-1-yl)acrylonitrile* (**7**): To a previously flamed round-bottom flask, under an argon atmosphere, 205 mg sodium hydride (60% wt.) and 10 mL of anhydrous tetrahydrofuran were added, then cooled to 0 °C. A solution containing 1.33 g (5.19 mmol) of diethyl(bromo(cyano)methyl)phosphonate previously dissolved in 20 mL of anhydrous tetrahydrofuran was added dropwise (ca 5 min) and stirred for 30 min (the solution acquired an intense red color). This solution was transferred via cannula (25 min) to a solution containing aldehyde 6 (500.0 mg, 3.37 mmol) in 30 mL of anhydrous tetrahydrofuran at −30 °C. The reaction mixture was stirred for 2 h at −30 °C and 12 h at 0 °C. After that, the reaction was quenched with 60 mL of saturated ammonium acetate solution and washed with dichloromethane (3 × 70 mL). The organic phases were united and dried over anhydrous sodium sulphate, filtered and the solvent removed under vacuum. The organic residue was purified by column chromatography on silica gel using the gradient hexane → hexane:ethyl acetate (9.5:0.5) as eluent, furnishing a yellow solid as product. Yield: 58% (486 mg; 1.94 mmol). **^1^H-NMR** (CDCl_3_, 400.15 MHz), *δ* (ppm): 2.96 (2H, s); 6.87–7.04 (5H, m); 7.15 (1H, s); 9.57 (1H, s). **^13^C-NMR** (CDCl_3_, 100.0 MHz) *δ* (ppm): 26.1; 87.7; 115.4; 129.6; 130.9; 132.0; 133.5; 135.3; 141.2; 148.3; 191.1. **IR:** *ν*_max_: (KBr pellet): 738 cm^−1^; 752 cm^−1^; 875 cm^−1^; 1149 cm^−1^; 1193 cm^−1^; 1232 cm^−1^; 1429 cm^−1^; 1516 cm^−1^; 1566 cm^−1^; 1585 cm^−1^; 1674 cm^−1^; 2205 cm^−1^; 2725 cm^−1^; 2821 cm^−1^. HRMS (ESI-TOF): **calc for** [M+Na]^+^, C_11_H_8_BrNNaO, 271.9681; **found**: 271.9690. **mp**: 92–94 °C.

*2-bromo-3-(6-((E)-2-cyanovinyl)cyclohepta-1,3,5-trien-1-yl)acrylonitrile* (**8**): To a previously flamed round-bottom flask, under an argon atmosphere, 970 mg (3.88 mmol) of compound **7** was dissolved in 50 mL of anhydrous tetrahydrofuran. This solution was cooled to −78 °C. In another flamed round-bottom flask with a suspension at 0 °C of 194 mg NaH (60% wt.) and 15 mL of anhydrous tetrahydrofuran, 810 µL (887 mg; 5.00 mmol) of diethylcyanomethylphosphonate was added dropwise (5 min). The mixture was left to react for 30 min, and then transferred via cannula to the solution containing compound **7** at −78 °C. The reaction mixture was left to react for 36 h, and then quenched with 50 mL of a saturated solution of NH_4_Cl and the reaction mixture was allowed to reach room temperature. The reaction mixture was extracted with dichloromethane (3 × 100 mL) and the organic layer washed with 50 mL of water. The organic layer was dried over anhydrous Na_2_SO_4_, filtered and the solvent removed under vacuum. The organic residue was purified by column chromatography on silica gel using the gradient hexane to hexane: AcOEt (9.5: 0.5) as eluent, furnishing a yellow solid as product. **Yield**: 30% (315 mg; 1.15 mmol). **^1^H-NMR** (CDCl_3_, 400.15 MHz), *δ* (ppm): 3.05 (2H, s); 5.34 (1H, d, *J* = 12.1 Hz); 6.27–6.91 (4H, m); 6.88 (1H, d, *J* = 12.1 Hz); 7.40 (1H, s). **^13^C**-**NMR** (CDCl_3_, 100 MHz) *δ* (ppm): 31.4; 86.3; 96.1; 116.0; 117.5; 127.4; 128.9; 132.6; 132.9; 133.1; 133.5; 148.2; 148.9. **IR:** *ν*_max_: (KBr pellet): 754 cm^−1^; 864 cm^−1^; 923 cm^−1^; 1088 cm^−1^; 1177 cm^−1^; 1246 cm^−1^; 1381 cm^−1^; 1445 cm^−1^; 1574 cm^−1^; 1589 cm^−1^; 2203 cm^−1^; 2849 cm^−1^; 2913 cm^−1^; 3059 cm^−1^; 3447 cm^−1^. **HRMS** (ESI-TOF): **calcd for** [M+H]^+^, C_13_H_10_BrN_2_, 273.0022, **found:** 273.0034. **mp:** 47–50 °C.

*(annulenonitrile) - bicyclo[4.4.1]undeca-1(10),2,4,6,8-pentaene-3,4-dicarbonitrile* (**9**): A solution of 233 mg (0.44 mmol) of compound **8** in 25 mL of freshly distilled DMF was degassed for 10 min under an argon atmosphere. Then, this solution was heated to 160 °C and stirred for 13 h. After this period, 50 mL of water was added and the reaction extracted with toluene (3 × 50 mL) The organic layer was washed with 50 mL of water, dried over anhydrous Na_2_SO_4_, filtered and the solvent removed under vacuum. The organic residue was purified by chromatography on neutral alumina utilizing gradient hexane to hexane: AcOEt (8:2) as eluent, furnishing a yellow solid as product. **Yield**: 52% (85.0 mg; 0.44 mmol). **^1^H-NMR** (CDCl_3_, 400,15 MHz), *δ* (ppm): –0.23 (1H, dt, ^2^*J* = 9.6 Hz, ^4^*J* = 1.2 Hz); −0.01 (1H, dt, ^2^*J* = 9.6 Hz, ^4^*J* = 1.2 Hz); 7.30–7.36 (2H, m); 7.53–7.60 (2H, m); 8.02(2H, s). **^13^C-NMR** (CDCl_3_, 100 MHz) *δ* (ppm): 34.1; 110.1; 115.8; 118.5; 129.9; 130.1; 137.3. **IR:** *ν*_max_: (KBr pellet): 718 cm^−1^; 870 cm^−1^; 908 cm^−1^; 1022 cm^−1^; 1261 cm^−1^; 1437 cm^−1^; 1458 cm^−1^; 2218 cm^−1^; 2851 cm^−1^; 2918 cm^−1^; 2962 cm^−1^; 3031 cm^−1^; 3445 cm^−1^. **HRMS** (ESI-TOF): **calc for** [M+H]^+^, C_13_H_9_N_2_^+^, 193.0760; **found:** 193.0759. **mp:** 185–187 °C.

*Zn(II)-1,6-methano[10]annulenecyanine* (**10**) via *annulenonitrile* (**9**): To a high pressure glass tube under an argon atmosphere were added 50.0 mg (260 µmol) annulenonitrile **9**, 24.2 mg (60.0 µmol) of zinc (II) triflate – Zn(OTf)_2_, 114 µL (87.8 mg; 540 µmol) of hexamethyldisilazane (HMDS) and 266 µL (253 mg; 3.46 mmol) of *N*,*N*-dimethylformamide (DMF). The reaction mixture was stirred at 120 °C for 24 h. After this period, the solvent was removed and the organic residue was purified by chromatography on silica gel utilizing CH_2_Cl_2_: MeOH (9.5:0.5) as eluent furnishing a green solid as product. For additional purification, it was necessary to utilize preparative TLC utilizing CH_2_Cl_2_:MeOH (9:1) as eluent. **Yield:** 63% (34.8 mg; 40.0 µmol). **UV-Vis** (DMF), *ν*_max_, (log ε): 362(4.89), 720(4.62), 800(5.07). **HRMS** (MALDI-TOF): **calc for** [M]^+^, C_52_H_32_N_8_Zn^+^, 832.2041, **found**: 832.2053.

*3-(triphenylphosphoranylidene)pyrrolidine-2,5-dione* (**12**): To a round-bottom flask containing 10 mL of acetic acid, 485.0 mg (5.00 mmol) of maleimide **11** and 1.38 g (5.25 mmol) of triphenylphosphine were added. The reaction mixture was stirred and refluxed at 125°C for 3.5 h. After this period, the acetic acid was removed under vacuum and the organic residue dissolved in acetone (10 mL), and then diethyl ether was slowly added resulting in the precipitation of an off-white solid. This solid was filtered, washed with diethyl ether (3 × 20 mL) and dried under vacuum. **Yield:** 90% (1.61 g; 4.49 mmol). **^1^H-NMR** (CDCl_3_, 400.15 MHz), *δ* (ppm): 1.65 (1H, bs); 3.03 (2H, s); 7.51–7.65 (15H, m). **^13^C-NMR** (CDCl_3_, 100.0 MHz) *δ* (ppm): 38.5; 125.1; 126.0; 128.6; 128.7; 128.8; 129.3; 129.4; 132.9; 133.0; 133.5; 133.6; 133.8; 134.0; 171.0; 178.0. **IR:** *ν*_max_: (KBr pellet): 3453 cm^−1^; 3111 cm^−1^; 3087 cm^−1^; 2961 cm^−1^; 2818 cm^−1^; 2743 cm^−1^; 1715 cm^−1^; 1616 cm^−1^, 1483 cm^−1^; 1435 cm^−1^, 1373 cm^−1^; 1312 cm^−1^, 1287 cm^−1^; 1213 cm^−1^_;_ 1167 cm^−1^; 1109 cm^−1^; 997 cm^−1^; 899 cm^−1^; 837 cm^−1^. **mp**: 218–220 °C.

*(annulenoimide) - 1H-5,10-methanocyclodeca[c]pyrrole-1,3(2H)-dione* (**13**): To a round-bottom flask containing 8 mL of acetic acid, 100 mg (0.68 mmol) of dialdehyde **6** and 364 mg (1.00 mmol) of phosphorane **12** were added. The reaction mixture was heated to 145 °C and stirred for 86 h. After this period, the acetic acid was removed under vacuum and the organic residue was purified by chromatography on silica gel utilizing hexane:AcOEt (6:4) as eluent, furnishing a yellow solid as product. **Yield:** 56% (80.7 mg; 0.38 mmol). **^1^H-NMR** (CDCl_3_, 400.15 MHz), *δ* (ppm): −0.20 (1H, dt, *J* = 9.9 Hz, *J* = 1.1 Hz); 0.10 (1H, dt, *J* = 9.9 Hz, *J* = 1.1 Hz); 7.32–7.38 (2H, m); 7.58–7.65 (2H, m); 7.96 (1H, bs); 8.27(2H, s). **^13^C-NMR** (CDCl_3_, 100.0 MHz) *δ* (ppm): 35.4; 119.5; 129.1; 129.4; 130.3; 130.8; 169.9. **IR:** *ν*_max_: (KBr pellet): 3184 cm^−1^; 3046 cm^−1^; 2955 cm^−1^; 2916 cm^−1^; 2848 cm^−1^; 1800 cm^−1^; 1757 cm^−1^; 1688 cm^−1^_,_ 1520 cm^−1^; 1422 cm^−1^, 1368 cm^−1^; 1163 cm^−1^_,_ 1020 cm^−1^; 872 cm^−1^_,_ 745 cm^−1^. **HRMS** (ESI-TOF): calc for [M+H]^+^, C_13_H_10_NO_2_^+^, 212.0706; **found:** 212.0706. **mp** 230–232 °C.

*Zn(II)-1,6-methano[10]annulenecyanine (10) via annulenoimide* (**13**): To a high pressure glass tube were added under an argon atmosphere 119.0 mg (560.0 µmol) of annulenoimide **13**, 51.2 mg (140 µmol) of Zn(OTf)_2_, 470 µL (360.0 mg; 2.23 mmol) of HMDS and 43 µL (41.8 mg; 573 µmol) of DMF. The reaction mixture was stirred at 160 °C for 24 h. After this period, the solvent was removed and the organic residue was purified by chromatography on silica gel utilizing CH_2_Cl_2_:MeOH (9.8:0.2) as eluent, furnishing a green solid as product. For additional purification, it was necessary to utilize preparative TLC utilizing CH_2_Cl_2_:MeOH (9.4:0.6) as eluent. **Yield:** 16% (22.5 mg; 27.0 µmol).

## 5. Conclusions

We have developed two different approaches for the first total synthesis of Zn(II)-1,6-methano[10]annulenecyanine (**10**). Multistep synthetic approaches for naphthalocyanine and phthalocyanine derivatives always presents low overall yields but are very necessary for dye-discovery with improved photochemical properties, particularly compounds with NIR absorption bands. This synthesis is the first part of the dye-discovery process and many additional photochemical and photophysical studies are necessary to achieve all the potential of **10** and related compounds. It is important to highlight that only preliminary studies on aggregation are reported in this communication. However, as mentioned before, this phenomenon should be evaluated in different solvents, with different techniques and, if possible, using the separated diastereoisomers, before presenting conclusions on self-association properties of **10** in solutions.

Herein, we have also demonstrated for the first time the potential of bridged-annulene derivatives as precursors for phthalocyanine and naphthalocyanine-like dyes, thus opening up many possibilities for the synthesis of hybrid structures with common precursors like phthalimides and phthalonitriles.

## Supplementary Materials

The following are available online. It contains details and characterization data along with copies of the ^1^H-NMR and ^13^C-NMR spectra and high-resolution mass spectra for compounds.
