# Supplementary material for: Synthesis of a Naphthalocyanine-Like Dye: The First Report on Zn(II)-1,6-methano[10]annulenecyanine
Source: Molecules. 2020 May 5;25(9):2164. doi: 10.3390/molecules25092164 (PMC7248870; doi:10.3390/molecules25092164)
Supplement: Supplementary file 1 [file molecules-25-02164-s001.pdf]

Supplementary

Synthesis of a Naphthalocyanine-like Dye: The First Report on Zn(II)-1,6-methano[10]annulenecyanine

1. General procedures

All reagents and starting materials and solvents were purchased from commercial sources and used as received or purified when necessary. Some reactions were carried out under an argon atmosphere as specified in the experimental procedures (see the supporting information). For NMR spectra (performed in $CDCl_3$ or in DMSO-d6 solutions) tetramethylsilane was used as an internal reference for ^1H (0 ppm), and C-D coupling signal as an internal reference for ^{13}C ($CDCl_3$—77.0 ppm and DMSO—39.5 ppm).

Flash chromatography was carried out using silica gel (230–400 mesh). Infrared spectra were registered using KBr cells for liquid (films) and KBr pellets for solids. Fluorescence emission spectrums were recorded using 1 cm optical length cuvettes at 25 °C and N,N-dimethylformamide as solvent. Analytical TLC was carried out on precoated aluminum sheets with silica gel (0.2 mm thick). UV–Vis analyses were performed using a double beam spectrometer with 0.1 nm of resolution. High resolution mass spectrometry was carried out on a MALDI-TOF for compound **10**, and ESI-TOF for compounds **2**, **3**, **4**, **5**, **6**, **7**, **8**, **9** and **13**.

2. Experimental Procedures

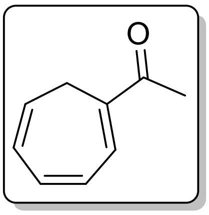

Figure S1. Compound **2**.

1-(cyclohepta-1,3,5-trien-1-yl)ethan-1-one (**2**): To a suspension of zinc (II) chloride (36.00 g, 264.0 mmol) in 23 mL of dichloromethane at –30 °C, under an argon atmosphere, 7.00 mL (7.73 g; 98.4 mmol) of acetyl chloride and 5.60 mL (5.87 g; 97.8 mmol) of acetic acid were added. Then, 3.40 mL (3.00 g; 32.4 mmol) of cycloheptatriene (**1**) was added dropwise (during *ca* 10 min). The reaction mixture was stirred for 3 h at –30 °C. The reaction mixture was quenched with 50 g of ice and neutralized with sodium bicarbonate. The organic layer was washed with brine (50 mL) and dried over anhydrous sodium sulphate. The solvent was removed under vacuum and the organic residue was purified by chromatography on silica gel using CH_2Cl_2 : hexano (5 : 5) → (6 : 4) as eluent, furnishing a yellow oil as product. **Yield:** 60% (2.63 g; 19.6 mmol). **^{1}H-NMR** ($CDCl_3$, 400.15 MHz), δ (ppm): 2.38 (s, 3H); 2.65 (d, 2H, J_1 = 7,0 Hz); 5.57 (dt, 1H, J_1 = 9.0 Hz, J_2 = 7.0 Hz); 6.27 (dd, 1H, J_1 = 9.3 Hz, J_2 = 5.7 Hz); 6.70 (ddt, 1H, J_1 = 11.2 Hz, J_2 = 6.0 Hz, J_3 = 0.8 Hz); 6.86 (dd, 1H, J_1 = 11.2 Hz, J_2 = 5.7 Hz); 7.09 (d, 1H, J = 6.0 Hz). **^{13}C-NMR** ($CDCl_3$, 100.0 MHz) δ (ppm): 25.4; 26.3; 125.9; 127.2; 129.3; 131.8; 133.1; 136.1; 197.6. **IR:** ν_{max}: (KBr): 3308 cm^{-1}; 3024 cm^{-1}; 2928 cm^{-1}; 2886 cm^{-1}; 2839 cm^{-1}; 1732 cm^{-1}; 1666 cm^{-1}; 1605 cm^{-1}; 1528 cm^{-1}; 1431 cm^{-1}; 1385 cm^{-1}; 1364 cm^{-1}; 1209 cm^{-1}; 1211 cm^{-1}; 1182 cm^{-1}; 976 cm^{-1}; 789 cm^{-1}; 758 cm^{-1}; 708 cm^{-1}. **HRMS** (ESI-TOF): **calc. for** $[M+H]^+$, $C_9H_{11}O^+$, 135.0804; **found:** 135.0806.

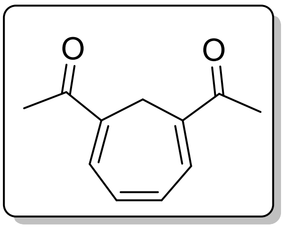

Figure S2. Compound **3**.

1,1′-(cyclohepta-3,5,7-triene-1,3-diyl)diethanone (**3**): In a suspension containing 1.49 g(11.2 mmol) of aluminium chloride and 6.00 mL of dry dichloromethane at 0 °C, 0.79 mL (870 mg; 11.2 mmol) of acetyl chloride was added under an argon atmosphere. After 5 min, 500.0 mg (3.73 mmol) of **2** was added. Then, the cooling bath was removed and the reaction mixture heated to 55 °C for 3 h. After this period, the reaction mixture was cooled to 0 °C and 5 mL of water at 5 °C was added. The reaction mixture was neutralized with sodium bicarbonate solution and filtered in a sintered funnel. The filtrate was washed with ethyl acetate (5 × 50 mL). The solvent was removed under vacuum and the organic residue (a brown oil) was purified by column chromatography on silica gel using toluene: ethyl acetate (95:5) as eluent, furnishing a yellow oil as product. **Yield:** 50% (333 mg; 1.89 mmol). **^{1}H-NMR** ($CDCl_3$, 400.15 MHz), δ (ppm): 2.24(s, 6H); 2.99(s, 2H); 6.98 (dd, 2H, J_1 = 4.0 Hz, J_2 = 3.0 Hz); 7.17 (dd, 2H, J_1 = 3.9 Hz, J_2 = 3.0 Hz). **^{13}C-NMR** ($CDCl_3$, 100.0 MHz) δ (ppm): 24.0; 26.7; 132.6; 134.1; 134.4; 197.1. **HRMS** (ESI-TOF): calc for $[M+H]^+$, $C_{11}H_{13}O_2^+$, 177.0910; **found:** 177.0912.

Note: Compound **3** is unstable under light and room temperature exposure. It must be stored at low temperature and protected from light.

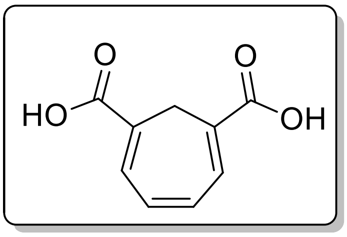

Figure S3. Compound **4**.

Cyclohepta-3,5,7-triene-1,3-dicarboxylic acid (**4**): To 150 mL aqueous sodium hydroxide solution (1.2 mol/L), 14 mL of 1,4-dioxane was added. Then this mixture was cooled to –5 °C and 2.60 mL of bromine (8.06 g; 50.7 mmol) was added. After 5 min, a solution containing 1.5 g (8.5 mmol) of compound **3** in 14 mL 1,4-dioxane was added dropwise (*ca* 10 min). Then, the reaction mixture was allowed to reach 5 °C and stirred for 15 h. After that, 100 mL of a solution 0.3 mol/L of sodium metabisulfite was added and stirred for 1h. The pH of the medium was adjusted to 3–4 with 10% solution of hydrochloric acid. The product was obtained as a precipitate which was filtered and washed with water (100 mL), ethanol (50 mL) and ethyl ether (50 mL) and dried. **Yield:** 62% (949 mg; 5.26 mmol). **^{1}H-NMR** (DMSO, 400.15 MHz), δ (ppm): 2.88 (s, 2H); 6,91 (t, 2H, J = 2.8 Hz); 7,19 (t, 2H, J = 2.8 Hz); 12.60 (s, 2H). **^{13}C-NMR** (DMSO, 100.0 MHz) δ (ppm): 25.1; 125.7; 132.4; 133.6; 166.5. **IR:** ν_{max}: (KBr pellet): 744 cm^{-1}; 905 cm^{-1}; 935 cm^{-1}; 1227 cm^{-1}; 1421 cm^{-1}; 1440 cm^{-1}; 1609 cm^{-1}; 1678 cm^{-1}; 2619 cm^{-1}; 2928 cm^{-1}; 2974 cm^{-1}; 3004 cm^{-1}; 3474 cm^{-1}. **HRMS** (ESI-TOF): calc for $[M+Na]^+$, $C_9H_8O_4Na^+$, 203.0315; found**:** 203.0317[1].

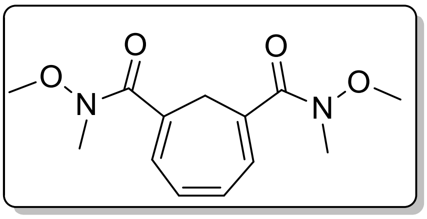

Figure S4. Compound **5**.

N^1,N^3-dimethoxy-N^1,N^3-dimethylcyclohepta-3,5,7-triene-1,3-dicarboxamide (**5**): To a suspension containing 900.0 mg (5.00 mmol) of diacid **4** in 60 mL of dichloromethane at 0 °C and under an argon atmosphere was added 1.65 g (16.9 mmol) of *N*-*O*-dimethylhydroxylamine hydrochloride, 390 mg (3.18 mmol) of *N*,*N*-Dimethyl-4-aminopyridine, 2.70 g (13.1 mmol) of *N*, *N'*-dicyclohexylcarbodiimide and 3.60 mL (2.61 g; 25.6 mmol) of triethylamine. The reaction mixture was stirred for 1 h at 0 °C, then 48 h at room temperature. After this period, the solvent was removed under vacuum and the residue partially dissolved with 150 mL of ethyl acetate/pentane mixture (1:1) and filtered off. The organic phase was ŧ concentrated under vacuum and the residue was purified by column chromatography on silica gel using dichloromethane/ethyl acetate (8:2) → (6:4) as eluent furnishing a yellow oil as product. **Yield:** 72% (956 mg; 3.60 mmol). **^{1}H-NMR** ($CDCl_3$, 400.15 MHz), δ (ppm): 2.84 (s, 2H); 3.18 (s, 6H); 3.53 (s, 6H); 6.62–6.63 (m, 4H). **^{13}C-NMR** ($CDCl_3$, 100.0 MHz) δ (ppm): 30.7; 33.4; 61.2; 129.4; 130.2; 132.0; 169.5. **HRMS** (ESI-TOF): calc. for $[M+H]^+$, $C_9H_9O_2^+$, 267.1339; **found:** 267.1341. **IR:** ν_{max}: (KBr): 750 cm^{-1}; 763 cm^{-1}; 973 cm^{-1}; 1275 cm^{-1}; 1380 cm^{-1}; 1610 cm^{-1}; 1634 cm^{-1}; 2934 cm^{-1}; 2963 cm^{-1}.

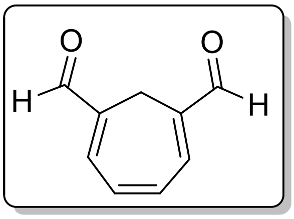

Figure S5. Compound **6**.

Cyclohepta-3,5,7-triene-1,3-dicarbaldehyde (**6**): To a suspension containing 2.73 g (71.9 mmol) of lithium aluminum hydride and 180 mL of anhydrous tetrahydrofuran at –78°C under an argon atmosphere, amide **5** (3.00 g; 11.3 mmol) previously dissolved in 80 mL of anhydrous tetrahydrofuran was added dropwise (*ca* 80 min). The reaction was stirred for 20 min, 100 mL of 0.50 mM aqueous solution of potassium bisulfate was added and the reaction allowed to reach 0 °C. Then 200 mL of 5% aqueous solution (*wt*/*v*) of citric acid was added and the reaction mixture was extracted with dichloromethane (3 × 100 mL). The organic layer was dried over anhydrous sodium sulphate, filtered and the solvent removed under vacuum (thermal bath at 20 °C). The aldehyde **6** was crystalized in an ethyl acetate/pentane mixture, furnishing a pale-yellow solid (999 mg; 6.73 mmol). The mother liquor was purified by chromatography on silica gel using CH_2Cl_2 → CH_2Cl_2:AcOEt (9:1) furnishing 156 mg (1.05 mmol). **Total Yield:** 69% (1.15 g, 7.78 mmol). **^{1}H-NMR** ($CDCl_3$, 400.15 MHz), δ (ppm): 3.08 (s, 2H); 7.00 (d, J = 2.7 Hz); 7.04 (dd, 2H, J_1 = 3.8 Hz, J_2 = 2.7 Hz); 9.54 (s, 2H). **^{13}C-NMR** ($CDCl_3$, 100.0 MHz) δ (ppm): 19.0; 134.2; 136.2; 141.3; 191.0. **IR:** ν_{max}: (KBr pellet): 1059 cm^{-1}; 1233 cm^{-1}; 1431 cm^{-1}; 1609 cm^{-1}; 1682 cm^{-1}; 2777 cm^{-1}, 2849 cm^{-1}, 2920 cm^{-1}, 3020 cm^{-1}, 3308 cm^{-1}, 3449 cm^{-1}. **HRMS** (ESI-TOF): **calc for** $[M+H]^+$, $C_9H_9O_2^+$, 149.0597; **found:** 149.0599. **mp:** 121–123 °C.

Diethyl(bromo(cyano)methyl)phosphonate: was synthesized according to the literature.[2]

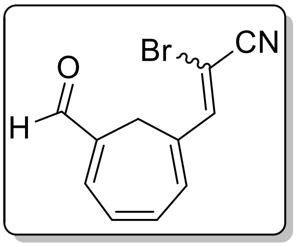

Figure S6. Compound **7**.

2-bromo-3-(6-formylcyclohepta-1,3,5-trien-1-yl)acrylonitrile (**7**): To a previously flamed round-bottom flask, under an argon atmosphere, 205 mg sodium hydride (60% wt.) and 10 mL of anhydrous

tetrahydrofuran were added, then cooled to 0 °C. A solution containing 1.33 g (5.19 mmol) of diethyl(bromo(cyano)methyl)phosphonate previously dissolved in 20 mL of anhydrous tetrahydrofuran was added dropwise (*ca* 5 min) and stirred for 30 min (the solution acquired an intense red colour). This solution was transferred via cannula (25 min) to a solution containing aldehyde **6** (500 mg, 3.37 mmol) in 30 mL of anhydrous tetrahydrofuran at –30 °C. The reaction mixture was stirred for 2 h at –30 °C and 12 h at 0 °C. After that, the reaction was quenched with 60 mL of saturated ammonium acetate solution and washed with dichloromethane (3 70 mL). The organic phases were united and dried over anhydrous sodium sulphate, filtered and the solvent removed under vacuum. The organic residue was purified by column chromatography on silica gel using the gradient hexane → hexane: ethyl acetate (9.5:0.5) as eluent, furnishing a yellow solid as product. **Yield:** 58% (486 mg; 1.94 mmol). **^{1}H-NMR** ($CDCl_3$, 400.15 MHz), δ (ppm): 2.96 (2H, s); 6.87–7.04 (5H, m); 7.15 (1H, s); 9.57 (1H, s). **^{13}C-NMR** ($CDCl_3$, 100.0 MHz) δ (ppm): 26.1; 87.7; 115.4; 129.6; 130.9; 132.0; 133.5; 135.3; 141.2; 148.3; 191.1. **IR:** ν_{max}: (KBr pellet): 738 cm^{-1}; 752 cm^{-1}; 875 cm^{-1}; 1149 cm^{-1}; 1193 cm^{-1}; 1232 cm^{-1}; 1429 cm^{-1}; 1516 cm^{-1}; 1566 cm^{-1}; 1585 cm^{-1}; 1674 cm^{-1}; 2205 cm^{-1}; 2725 cm^{-1}; 2821. **HRMS** (ESI-TOF): **calc** for [M+Na]$^+$, $C_{11}H_8BrNNaO$, 271.9681; **found:** 271.9690. **mp:** 92–94 °C.

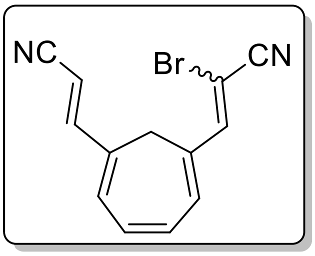

Figure S7. Compound **8**.

2-bromo-3-(6-((E)-2-cyanovinyl)cyclohepta-1,3,5-trien-1-yl)acrylonitrile (**8**): To a previously flamed round-bottom flask, under an argon atmosphere, 970 mg (3.88 mmol) of compound **7** was dissolved in 50 mL of anhydrous tetrahydrofuran. This solution was cooled to –78 °C. In another flamed round-bottom flask with a suspension at 0 °C of 194 mg NaH (60 % wt.) and 15 mL of anhydrous tetrahydrofuran, 810 µL (887 mg; 5.01 mmol) of diethylcyanomethylphosphonate was added dropwise (5 min). The mixture was left to react for 30 min, and then transferred via cannula to the solution containing compound **7** at –78 °C. The reaction mixture was left to react for 36 h, and then quenched with 50 mL of a saturated solution of NH_4Cl and the reaction mixture was allowed to reach room temperature. The reaction mixture was extracted with dichloromethane (3 × 100 mL) and the organic layer washed with 50 mL of water. The organic layer was dried over anhydrous Na_2SO_4, filtered and the solvent removed under vacuum. The organic residue was purified by column chromatography on silica gel using the gradient hexane to hexane: AcOEt (9.5:0.5) as eluent, furnishing a yellow solid as product. **Yield**: 30% (315 mg; 1.15 mmol). **^{1}H-NMR** ($CDCl_3$, 400.15 MHz), δ (ppm): 3.05 (2H, s); 5.34 (1H, d, ^3J = 12.1 Hz); 6.27–6.91 (4H, m); 6.88 (1H, d, ^3J = 12.1 Hz); 7.40 (1H, s). **^{13}C-NMR** ($CDCl_3$, 100 MHz) δ (ppm): 31.4; 86.3; 96.1; 116.0; 117.5; 127.4; 128.9; 132.6; 132.9; 133.1; 133.5; 148.2; 148.9. **IR:** ν_{max}: (KBr pellet): 754 cm^{-1}; 864 cm^{-1}; 923 cm^{-1}; 1088 cm^{-1}; 1177 cm^{-1};1246 cm^{-1}; 1381 cm^{-1}; 1445 cm^{-1}; 1574 cm^{-1}; 1589 cm^{-1}; 2203 cm^{-1}; 2849 cm^{-1}; 2913 cm^{-1}; 3059 cm^{-1}; 3447 cm^{-1}. **HRMS** (ESI-TOF): calcd for [M+H]$^+$, $C_{13}H_{10}BrN_2$, 273.0022 **found:** 273.0034. **mp:** 47–50 °C.

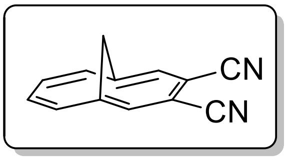

Figure S8. Compound **9**.

(annulenonitrile) - bicyclo[4.4.1]undeca-1(10),2,4,6,8-pentaene-3,4-dicarbonitrile (**9**): A solution of 233 mg (0.44 mmol) of compound **8** in 25 mL of freshly distilled DMF was degassed for 10 min under an argon atmosphere. Then, this solution was heated to 160 °C and stirred for 13 h. After this period, 50 mL of water was added and the reaction extracted with toluene (3 × 50 mL) The organic layer was washed with 50 mL of water, dried over anhydrous Na_2SO_4, filtered and the solvent removed under vacuum. The organic residue was purified by chromatography on neutral alumina utilizing gradient hexane to hexane: AcOEt (8:2) as eluent, furnishing a yellow solid as product. **Yield**: 52 % (85.0 mg; 0.44 mmol). **^{1}H-NMR** ($CDCl_3$, 400,15 MHz), δ (ppm): –0.23 (1H, dt, ^2J = 9.6 Hz, ^4J = 1.2 Hz); –0.01 (1H, dt, ^2J = 9.6 Hz, ^4J = 1.2 Hz); 7.30–7.36 (2H, m); 7.53–7.60 (2H, m); 8.02(2H, s). **^{13}C-NMR** ($CDCl_3$, 100 MHz) δ (ppm): 34.1; 110.1; 115.8; 118.5; 129.9; 130.1; 137.3. **IR:** ν_{max}: (KBr pellet): 718 cm^{-1}; 870 cm^{-1}; 908 cm^{-1}; 1022 cm^{-1}; 1261 cm^{-1}; 1437 cm^{-1}; 1458 cm^{-1}; 2218 cm^{-1}; 2851 cm^{-1}; 2918 cm^{-1}; 2962 cm^{-1}; 3031 cm^{-1}; 3445 cm^{-1}. **HRMS** (ESI-TOF): **calc for** $[M+H]^+$, $C_{13}H_9N_2^+$, 193.0760; **found:** 193.0759. **mp:** 185–187 °C. **Literature:** 194–195 °C [3].

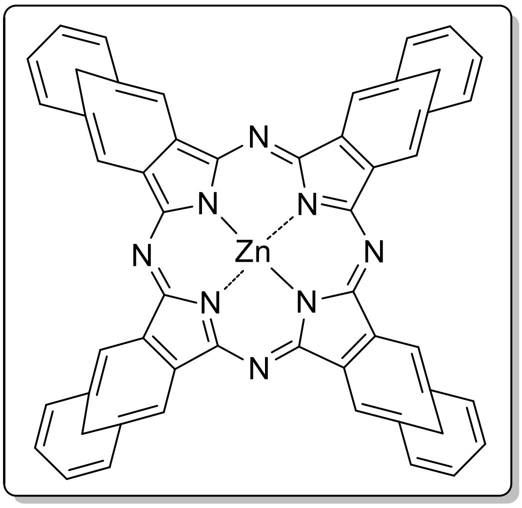

Figure S9. Compound **10**.

Zn(II)-1,6-methano[10]annulenecyanine (**10**) via *annulenonitrile* (**9**): To a high pressure glass tube under an argon atmosphere were added 50.0 mg (260 µmol) annulenonitrile **9**, 24.2 mg (60.0 µmol) of zinc (II) triflate - $Zn(OTf)_2$, 114 µL (87.8 mg; 540 µmol) of hexamethyldisilazane (HMDS) and 266 µL (253 mg; 3.46 mmol) of *N*,*N*-dimethylformamide (DMF). The reaction mixture was stirred at 120 °C for 24 h. After this period, the solvent was removed and the organic residue was purified by chromatography on silica gel utilizing CH_2Cl_2: MeOH (9.5:0.5) as eluent furnishing a green solid as product. For additional purification, it was necessary to utilize preparative TLC utilizing CH_2Cl_2 : MeOH (9:1) as eluent. **Yield:** 63% (34.8 mg; 40.0 µmol). **UV–Vis** (DMF), λ_{max}, (logε): 362(4.89), 720(4.62), 800(5.07). **HRMS** (MALDI-TOF): **calc for** $[M]^+$, $C_{52}H_{32}N_8Zn^+$, 832.2041, found: 832.2053.

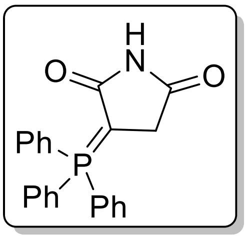

Figure S10. Compound **12**.

3-(triphenylphosphoranylidene)pyrrolidine-2,5-dione (**12**): To a round-bottom flask containing 10 mL of acetic acid, 485.0 mg (5.00 mmol) of maleimide **11** and 1.38 g (5.25 mmol) of triphenylphosphine were added. The reaction mixture was stirred and refluxed at 125 °C for 3.5 h. After this period, the acetic

acid was removed under vacuum and the organic residue dissolved in acetone (10 mL), and then diethyl ether was slowly added resulting in the precipitation of an off-white solid. This solid was filtered, washed with diethyl ether (3 × 20 mL) and dried under vacuum. **Yield:** 90% (1.61 g; 4.49 mmol). **^{1}H-NMR** ($CDCl_3$, 400.15 MHz), δ (ppm): 1.65 (1H, bs); 3.03 (2H, s); 7.51–7.65 (15H, m). **^{13}C-NMR** ($CDCl_3$, 100.0 MHz) δ (ppm): 38.5; 125.1; 126.0; 128.6; 128.7; 128.8; 129.3; 129.4; 132.9; 133.0; 133.5; 133.6; 133.8; 134.0; 171.0; 178.0. **IR:** ν_{max}: (KBr pellet): 3453 cm^{-1}; 3111 cm^{-1}; 3087 cm^{-1}; 2961 cm^{-1}; 2818 cm^{-1}; 2743 cm^{-1}; 1715 cm^{-1}; 1616 cm^{-1}, 1483 cm^{-1}; 1435 cm^{-1}, 1373 cm^{-1}; 1312 cm^{-1}, 1287 cm^{-1}; 1213 cm^{-1}; 1167 cm^{-1}; 1109 cm^{-1}; 997 cm^{-1}; 899 cm^{-1}; 837 cm^{-1}. **mp**: **found:** 218–220 °C.[4,5].

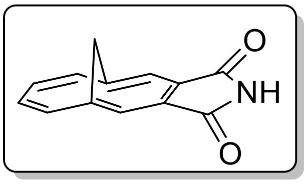

Figure S11. Compound **12**.

(annulenoimide)-1H-5,10-methanocyclodeca[c]pyrrole-1,3(2H)-dione (**13**): To a round-bottom flask containing 8 mL of acetic acid, 100 mg (0.68 mmol) of dialdehyde **6** and 364 mg (1.00 mmol) of phosphorane **12** were added. The reaction mixture was heated to 145 °C and stirred for 86 h. After this period, the acetic acid was removed under vacuum and the organic residue was purified by chromatography on silica gel utilizing hexane : AcOEt (6:4) as eluent, furnishing a yellow solid as product. Yield: 56% (80.7 mg; 0.38 mmol). **^{1}H-NMR** ($CDCl_3$, 400.15 MHz), δ (ppm): –0.20 (1H, dt, J = 9.9 Hz, J = 1.1 Hz); 0.10 (1H, dt, J = 9.9 Hz, J = 1.1 Hz); 7.32–7.38 (2H, m); 7.58–7.65 (2H, m); 7.96 (1H, bs); 8.27(2H, s). **^{13}C-NMR** ($CDCl_3$, 100.0 MHz) δ (ppm): 35.4; 119.5; 129.1; 129.4; 130.3; 130.8; 169.9. **IR**: ν_{max}: (KBr pellet): 3184 cm^{-1}; 3046 cm^{-1}; 2955 cm^{-1}; 2916 cm^{-1}; 2848 cm^{-1}; 1800 cm^{-1}; 1757 cm^{-1}; 1688 cm^{-1}, 1520 cm^{-1}; 1422 cm^{-1}, 1368 cm^{-1}; 1163 cm^{-1}, 1020 cm^{-1}; 872 cm^{-1}, 745 cm^{-1}. HRMS (ESI-TOF): calc for $[M+H]^+$, $C_{13}H_{10}NO_2^+$, 212.0706; found: 212.0706. mp found: 230–232 °C [4,5].

Zn(II)-1,6-methano[10]annulenecyanine (**10**) via *annulenoimide* (**13**): To a high pressure glass tube were added under an argon atmosphere 119 mg (560 µmol) of annulenoimide **13**, 51.2 mg (140 µmol) of $Zn(OTf)_2$, 470 µL (360 mg; 2.23 mmol) of HMDS and 43 µL (41.8 mg; 573 µmol) of DMF. The reaction mixture was stirred at 160 °C for 24 h. After this period, the solvent was removed and the organic residue was purified by chromatography on silica gel utilizing CH_2Cl_2 : MeOH (9.8:0.2) as eluent, furnishing a green solid as product. For additional purification, it was necessary to utilize preparative TLC utilizing CH_2Cl_2 : MeOH (9,4:0,6) as eluent. **Yield:** 16% (22.5 mg; 27.0 µmol).

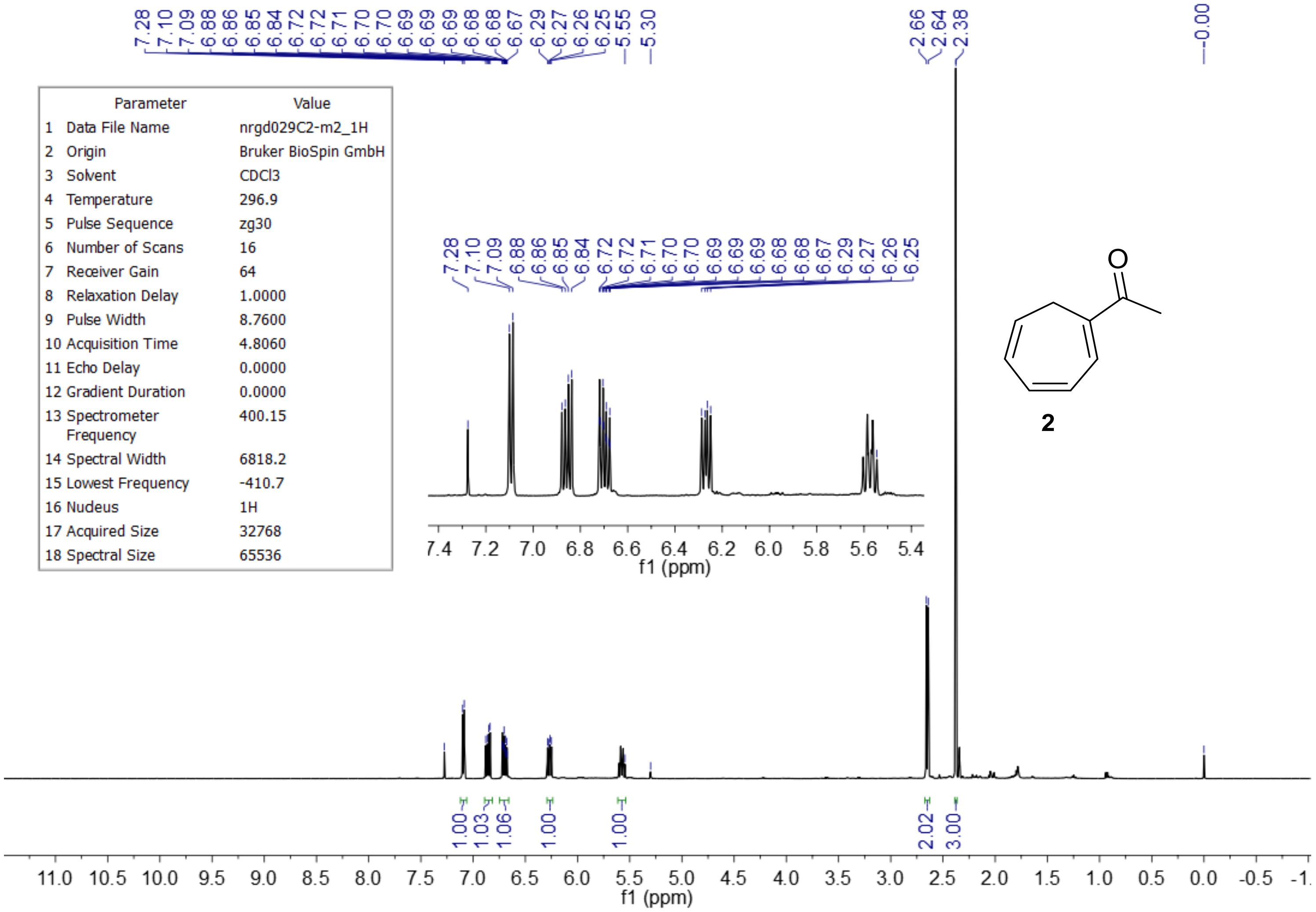

Figure S12. ^{1}H-NMR (400 MHz) ($CDCl_3$)—compound **2**.

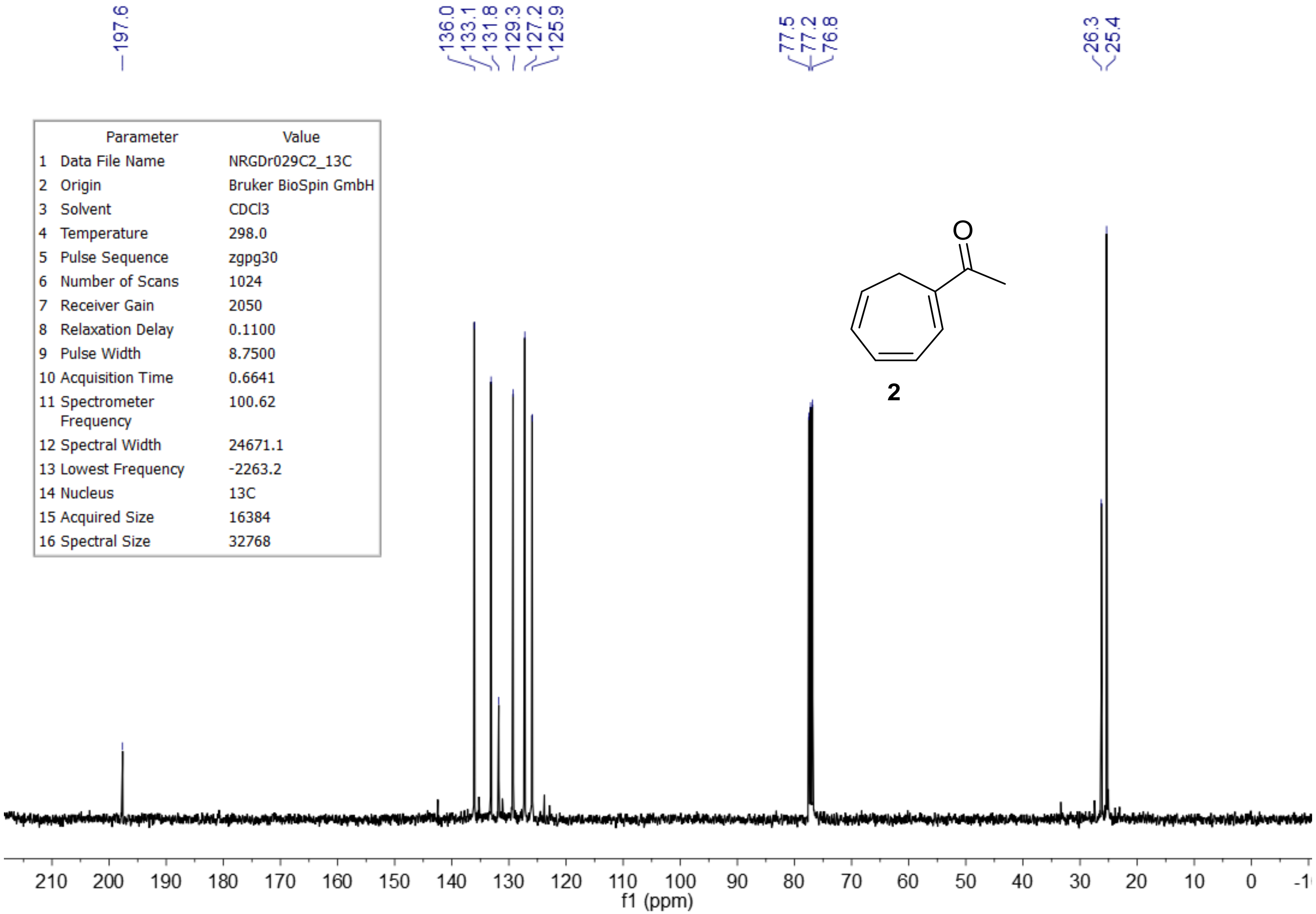

	Parameter	Value
1	Data File Name	NRGDr029C2_13C
2	Origin	Bruker BioSpin GmbH
3	Solvent	CDCl3
4	Temperature	298.0
5	Pulse Sequence	zgpg30
6	Number of Scans	1024
7	Receiver Gain	2050
8	Relaxation Delay	0.1100
9	Pulse Width	8.7500
10	Acquisition Time	0.6641
11	Spectrometer Frequency	100.62
12	Spectral Width	24671.1
13	Lowest Frequency	-2263.2
14	Nucleus	13C
15	Acquired Size	16384
16	Spectral Size	32768

Figure S13. $^{13}C\ \{^{1}H\}$ (100 MHz)—compound **2**.

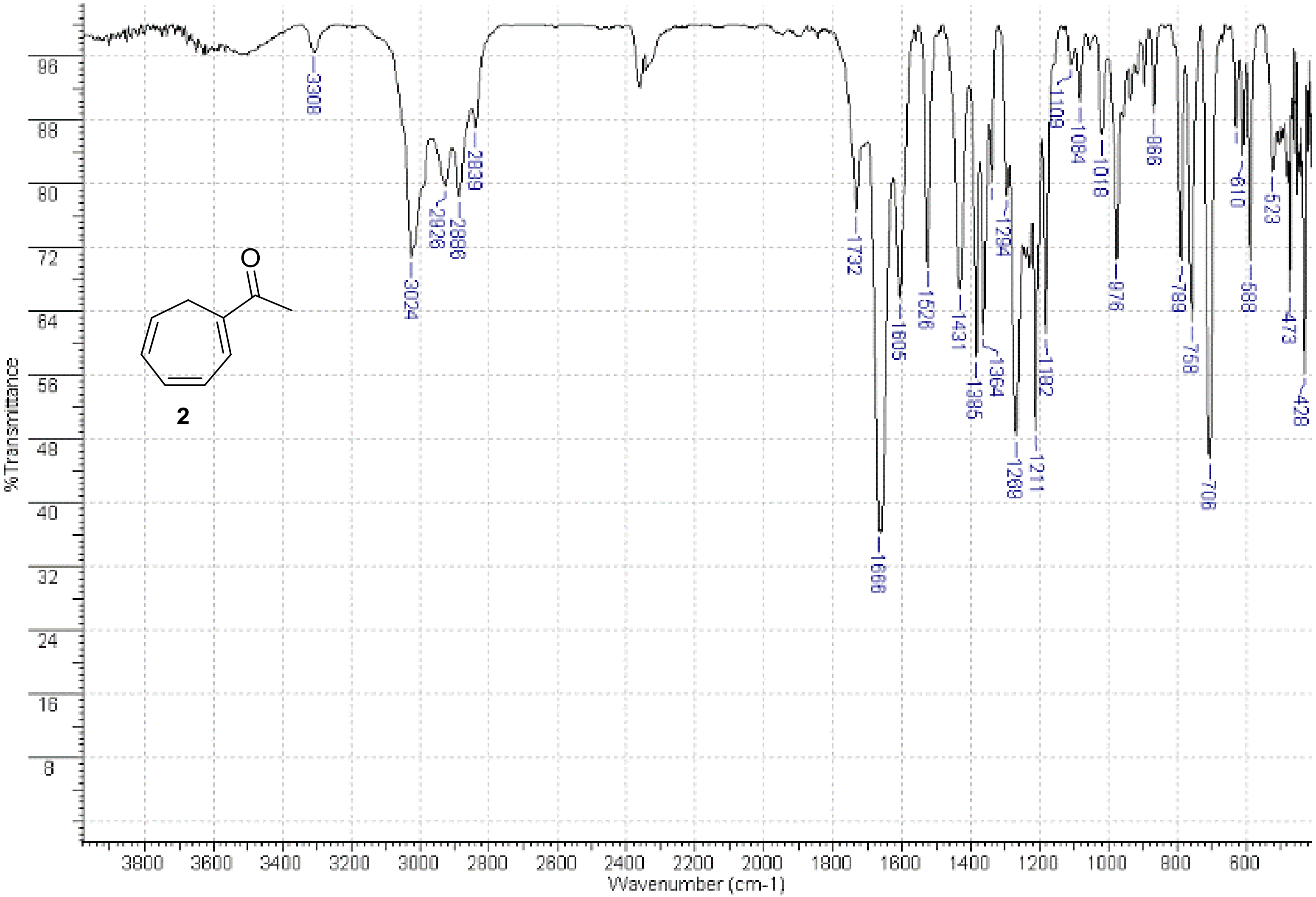

Figure S14. IR (KBr cell)—compound **2**.

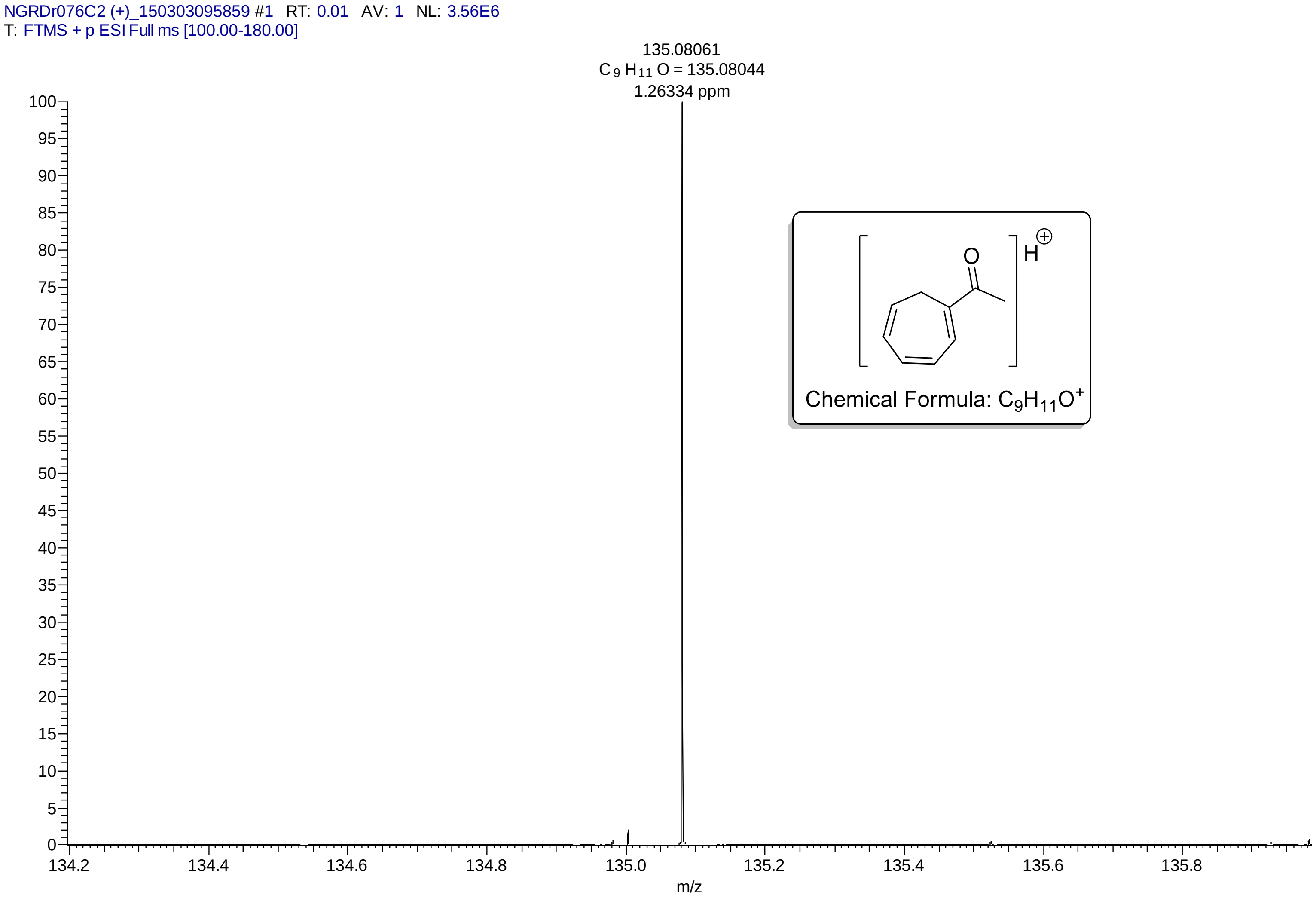

Figure S15. HRMS—ESI-TOF—compound **2**.

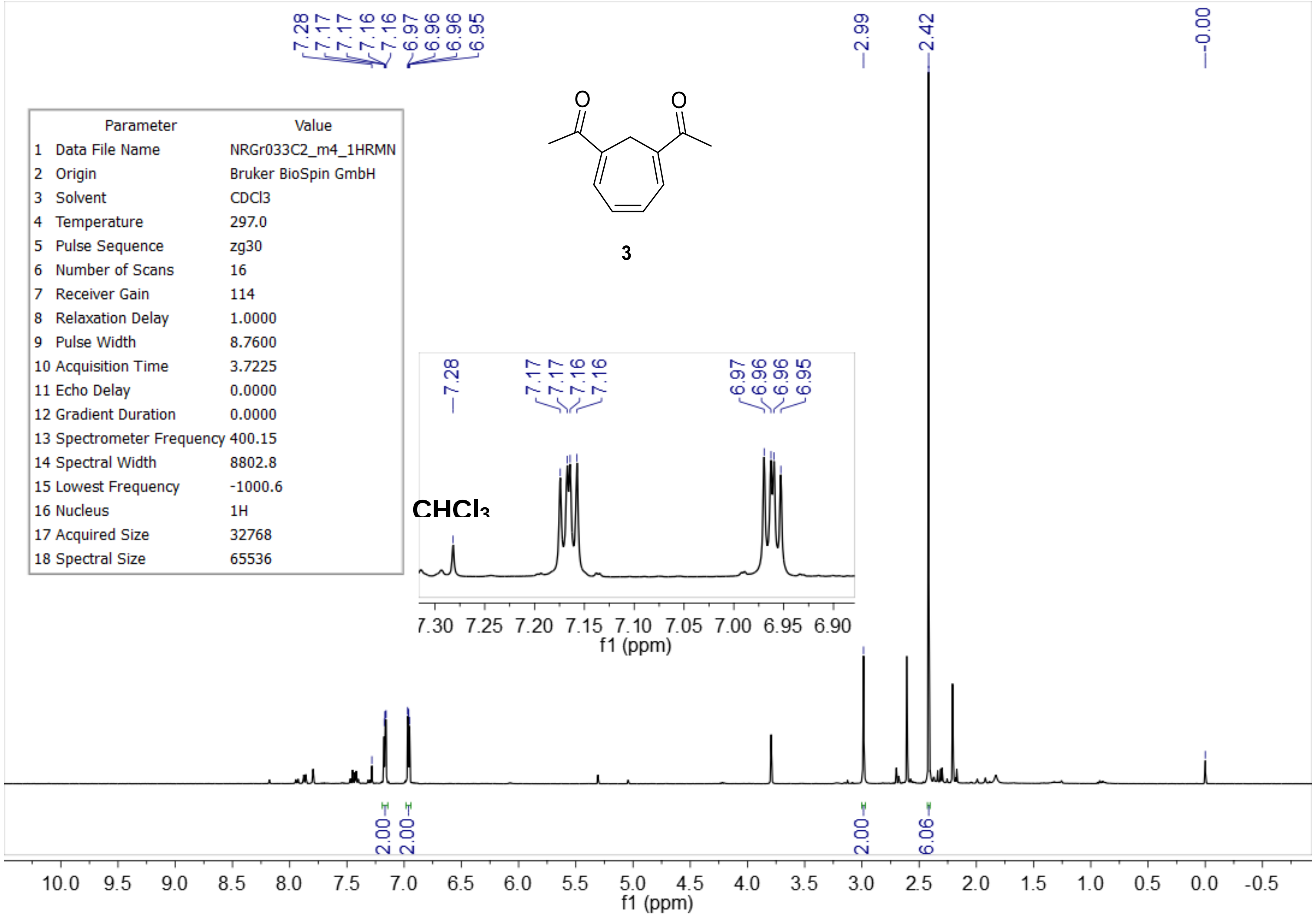

Figure S16. ^{1}H-NMR (400 MHz) ($CDCl_3$)—compound **3**.

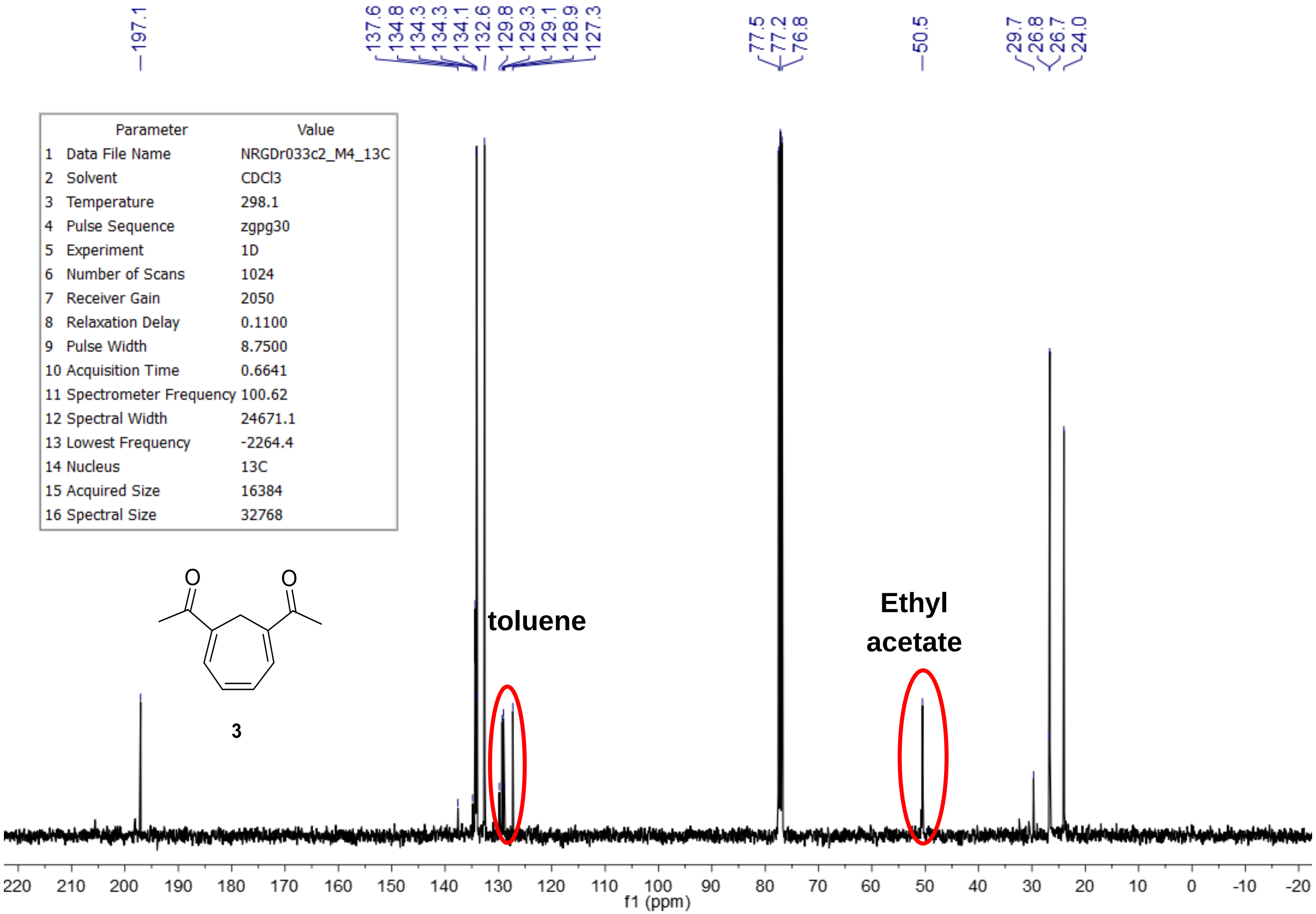

Figure S17. $^{13}C\ \{^1H\}$ (100 MHz) ($CDCl_3$)—compound **3**.

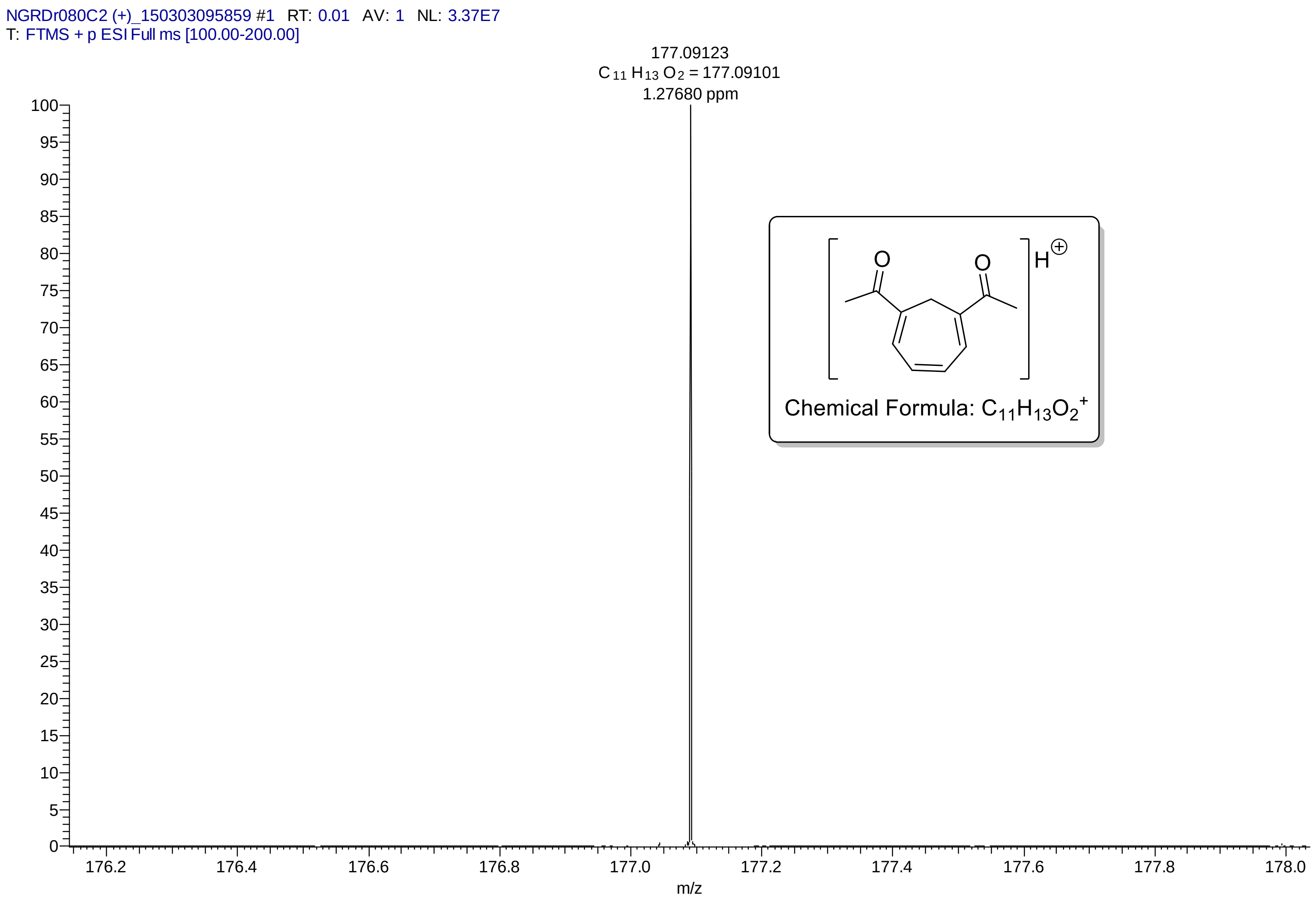

Figure S18. HRMS—ESI-TOF—compound **3**.

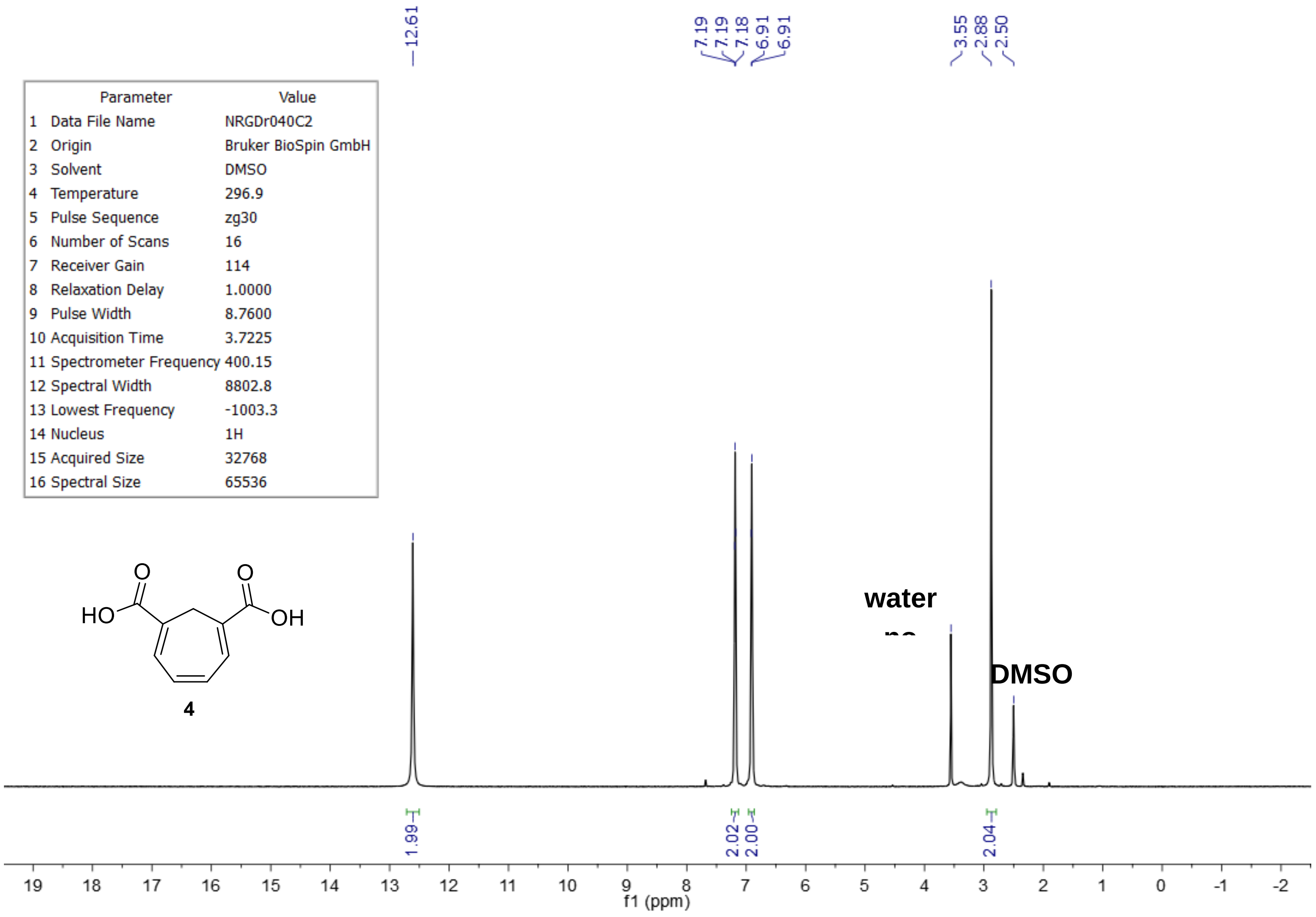

	Parameter	Value
1	Data File Name	NRGDr040C2
2	Origin	Bruker BioSpin GmbH
3	Solvent	DMSO
4	Temperature	296.9
5	Pulse Sequence	zg30
6	Number of Scans	16
7	Receiver Gain	114
8	Relaxation Delay	1.0000
9	Pulse Width	8.7600
10	Acquisition Time	3.7225
11	Spectrometer Frequency	400.15
12	Spectral Width	8802.8
13	Lowest Frequency	-1003.3
14	Nucleus	1H
15	Acquired Size	32768
16	Spectral Size	65536

Figure S19. ^{1}H-NMR (400 MHz) ($CDCl_3$)—compound **4**.

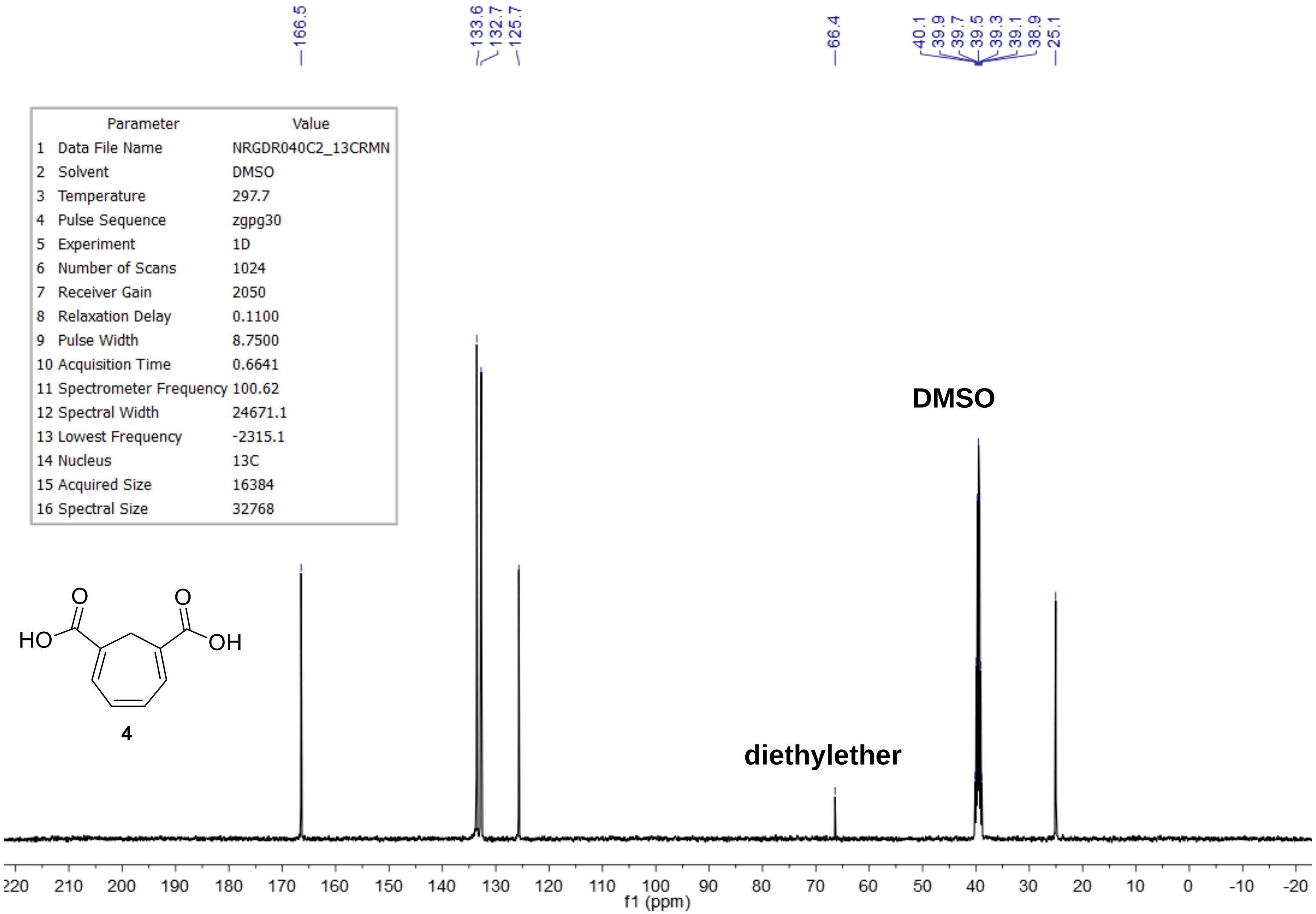

Figure S20. ^{13}C {^{1}H} (100 MHz) (DMSO-d6)—compound **4**.

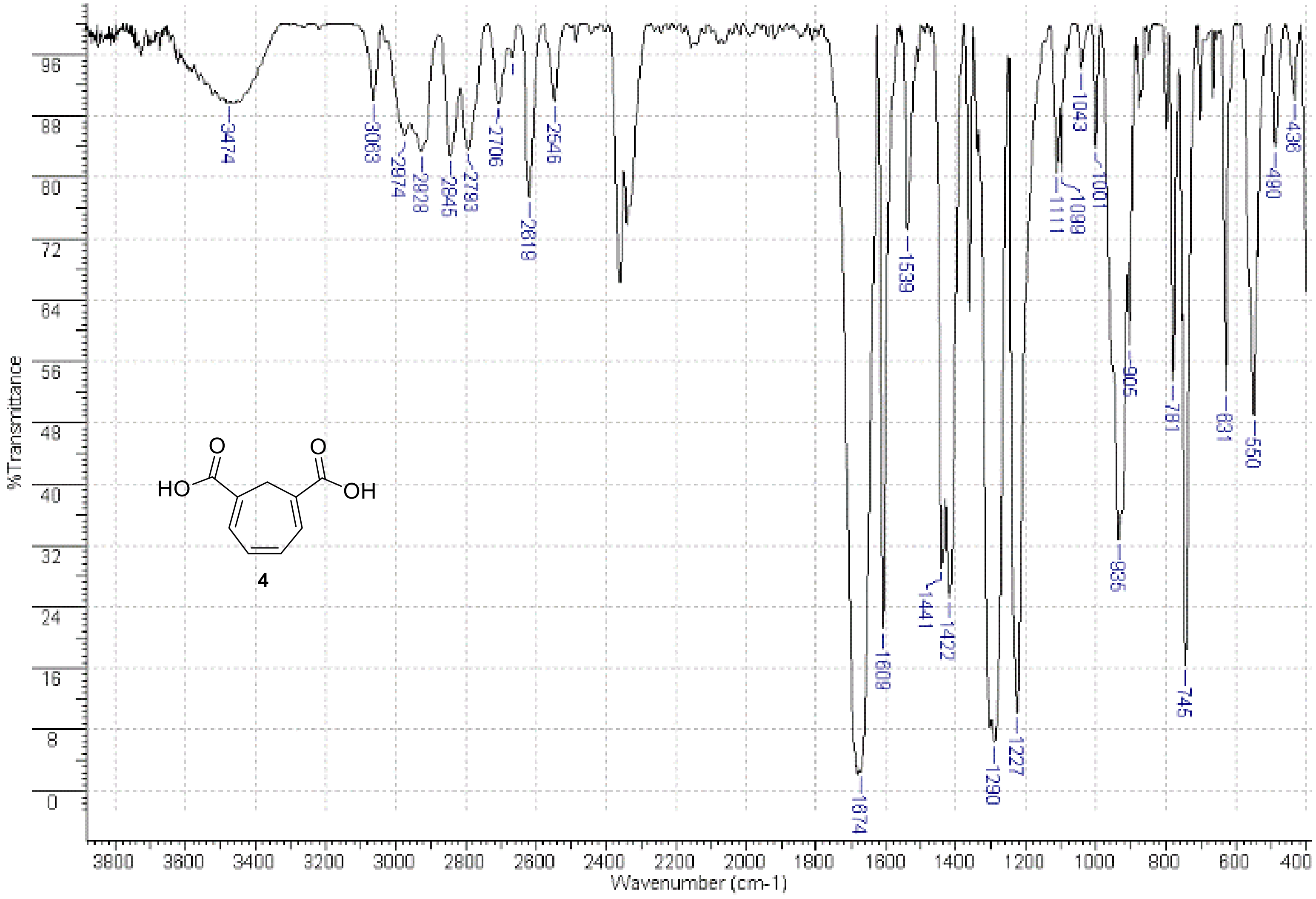

Figure S21. IR (KBr pellet)—compound **4**.

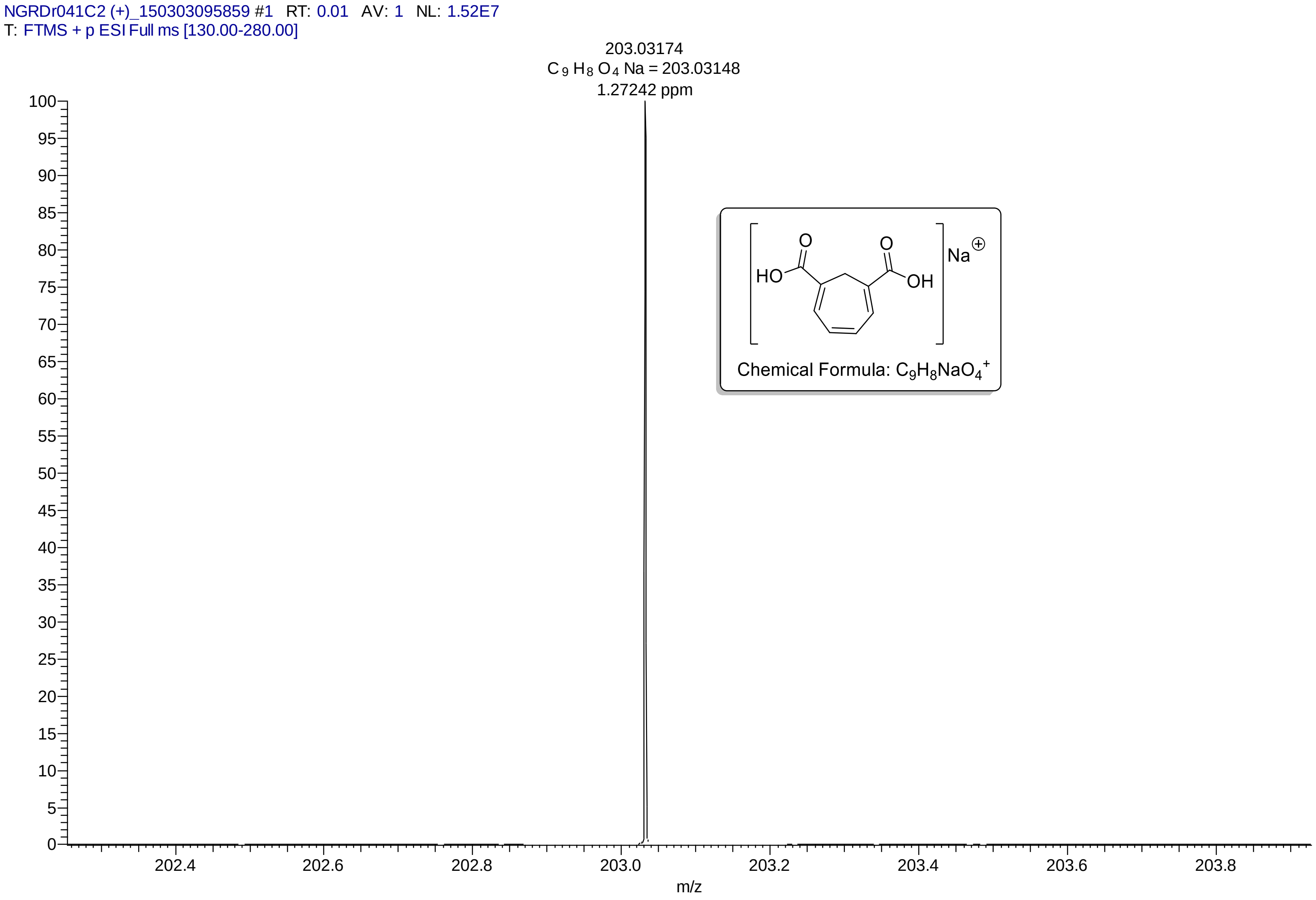

Figure S22. HRMS—ESI-TOF—compound **4**.

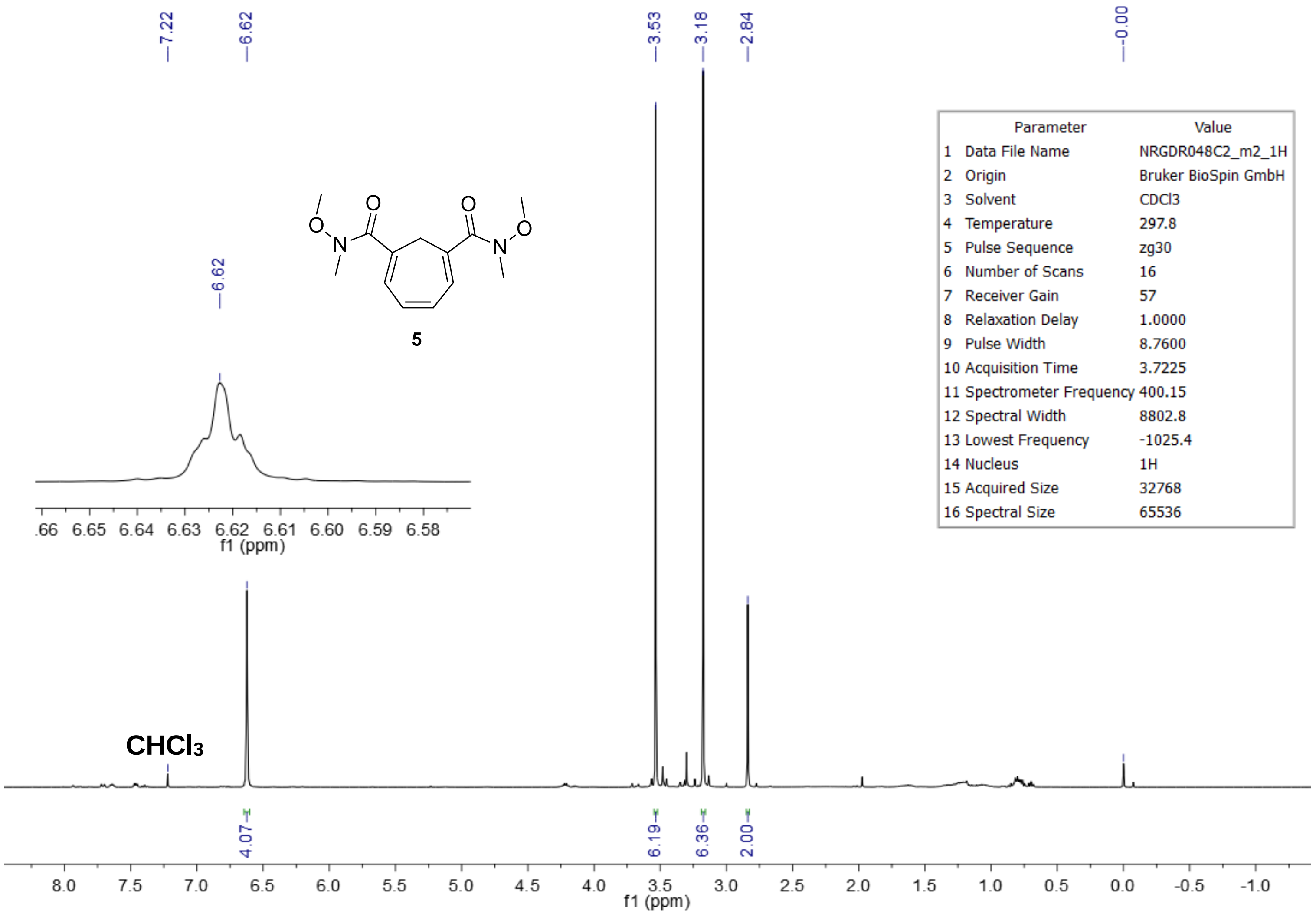

	Parameter	Value
1	Data File Name	NRGDR048C2_m2_1H
2	Origin	Bruker BioSpin GmbH
3	Solvent	CDCl3
4	Temperature	297.8
5	Pulse Sequence	zg30
6	Number of Scans	16
7	Receiver Gain	57
8	Relaxation Delay	1.0000
9	Pulse Width	8.7600
10	Acquisition Time	3.7225
11	Spectrometer Frequency	400.15
12	Spectral Width	8802.8
13	Lowest Frequency	-1025.4
14	Nucleus	1H
15	Acquired Size	32768
16	Spectral Size	65536

Figure S23. ^{1}H-NMR (400 MHz) ($CDCl_3$)—compound **5**.

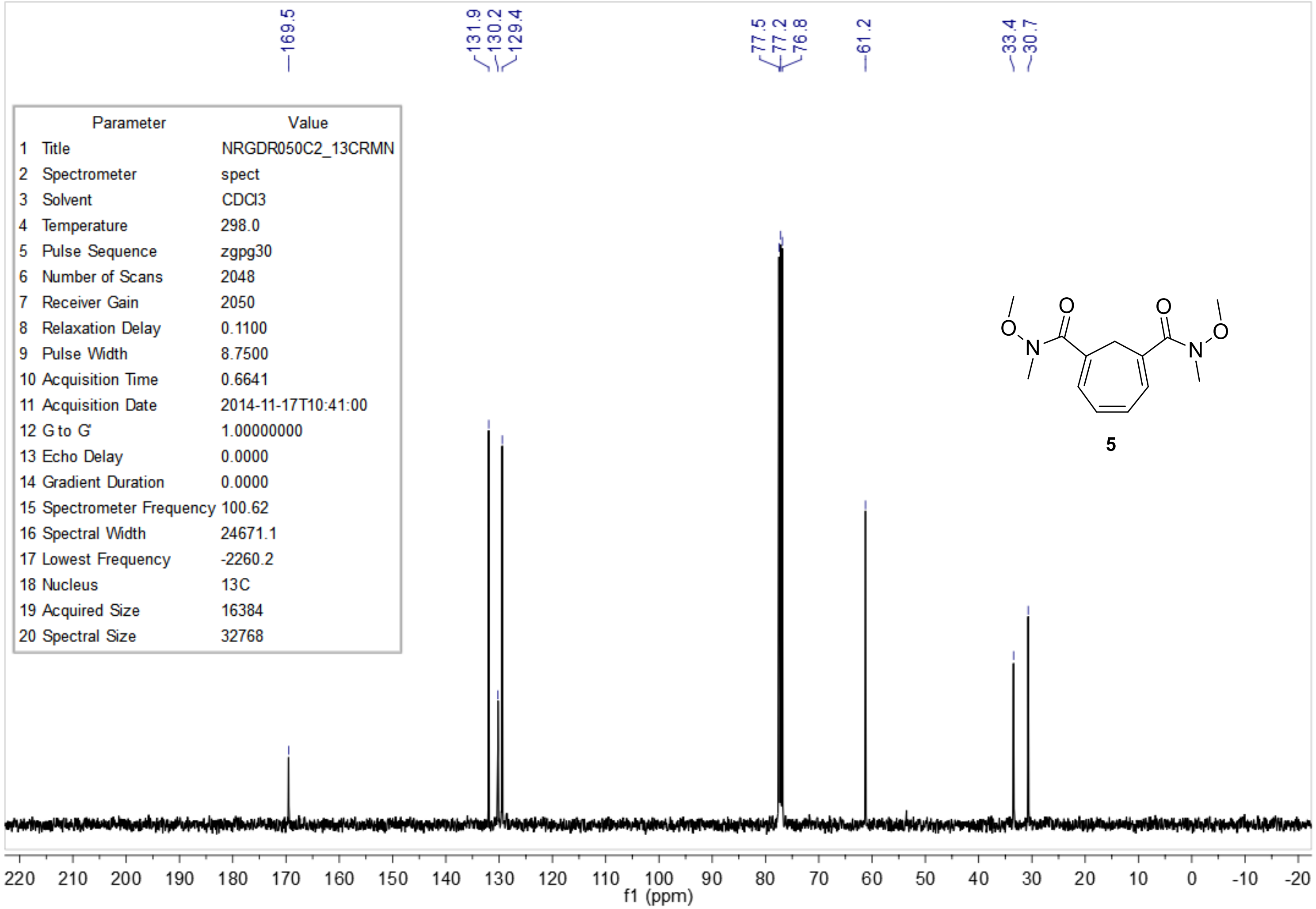

Figure S24. ^{13}C {^{1}H} (100 MHz) ($CDCl_3$)—compound **5**.

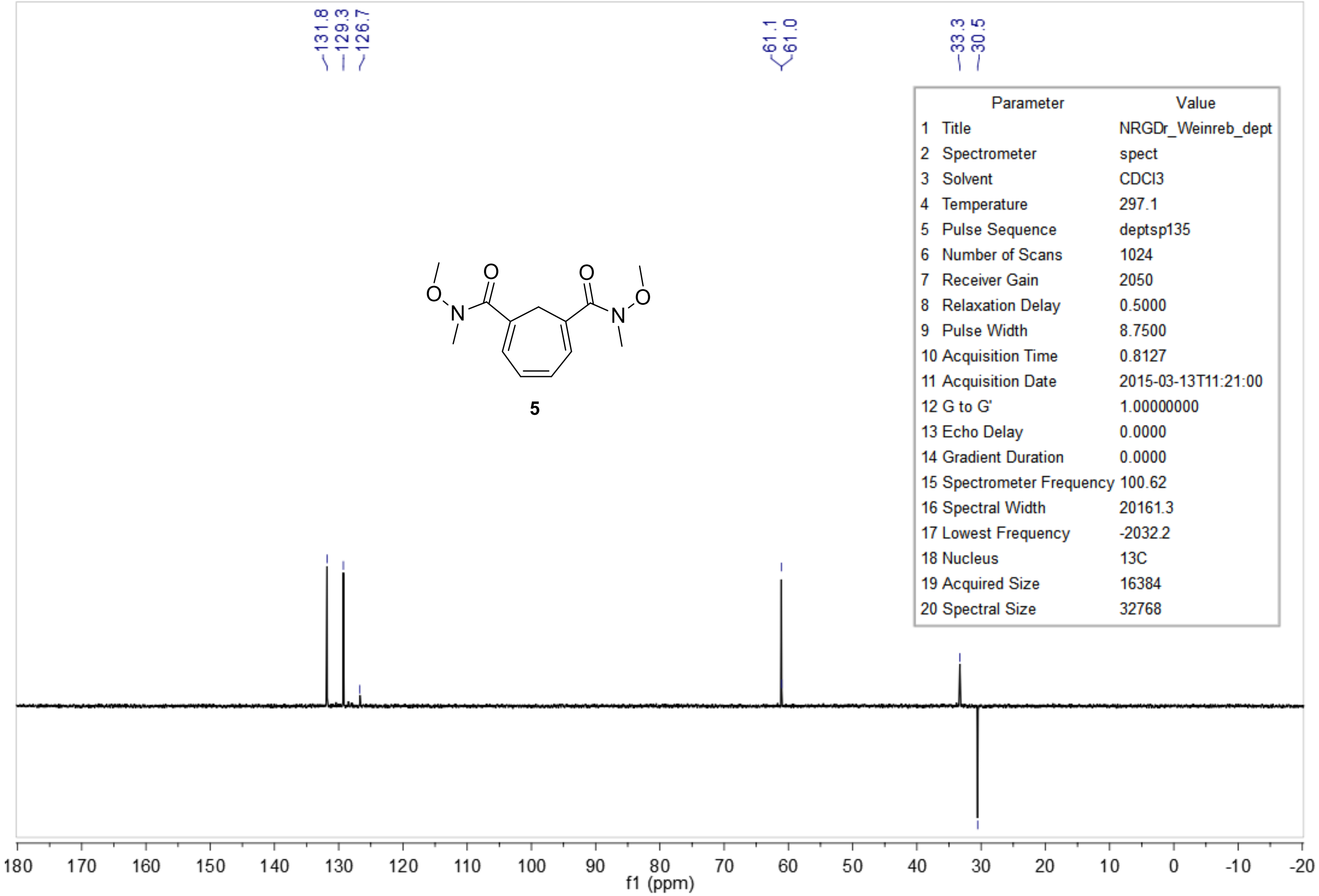

Figure S25. ^{13}C (DEPT-135) ($CDCl_3$)—compound **5**.

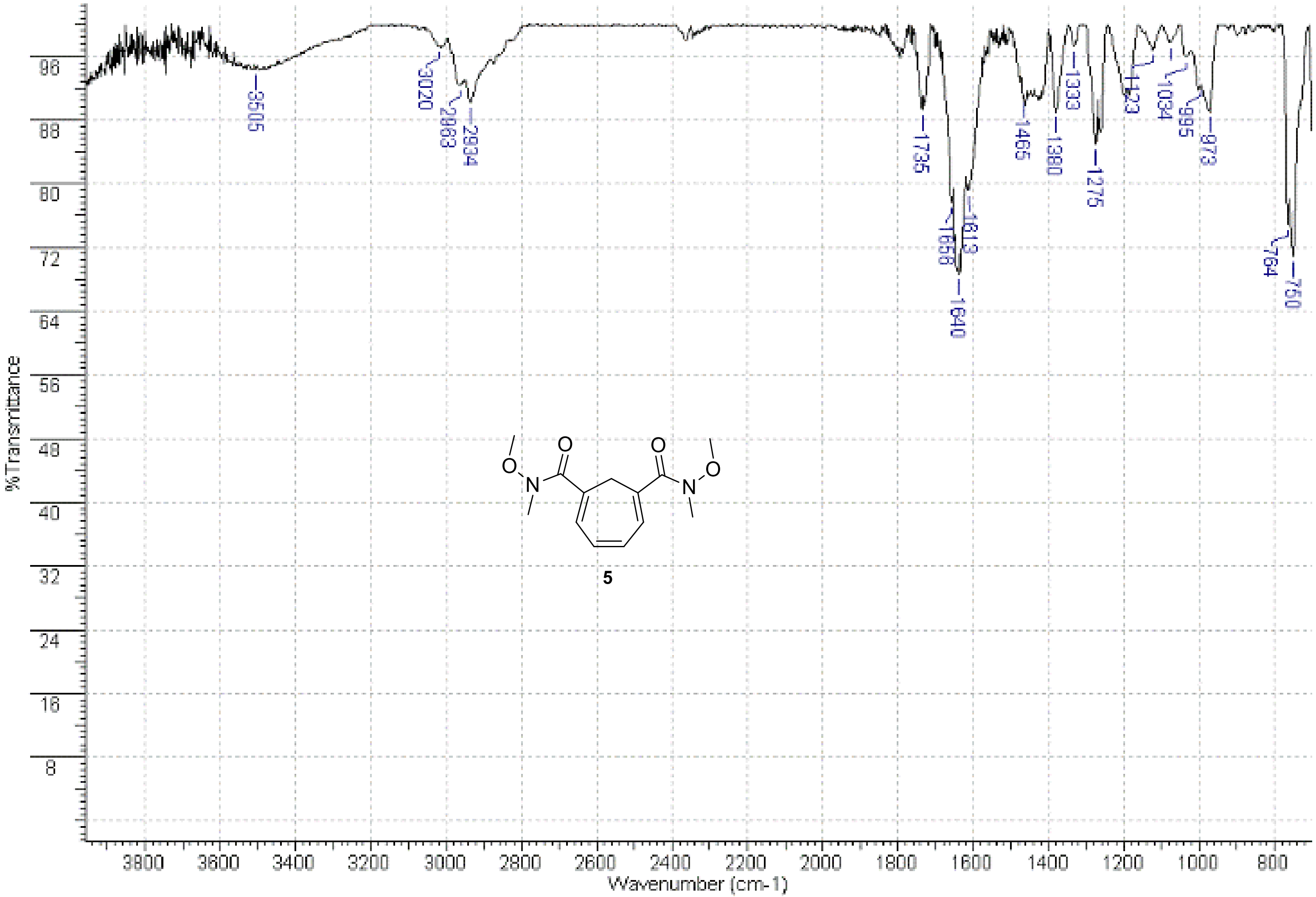

Figure S26. IR (KBr cell)—compound **5**.

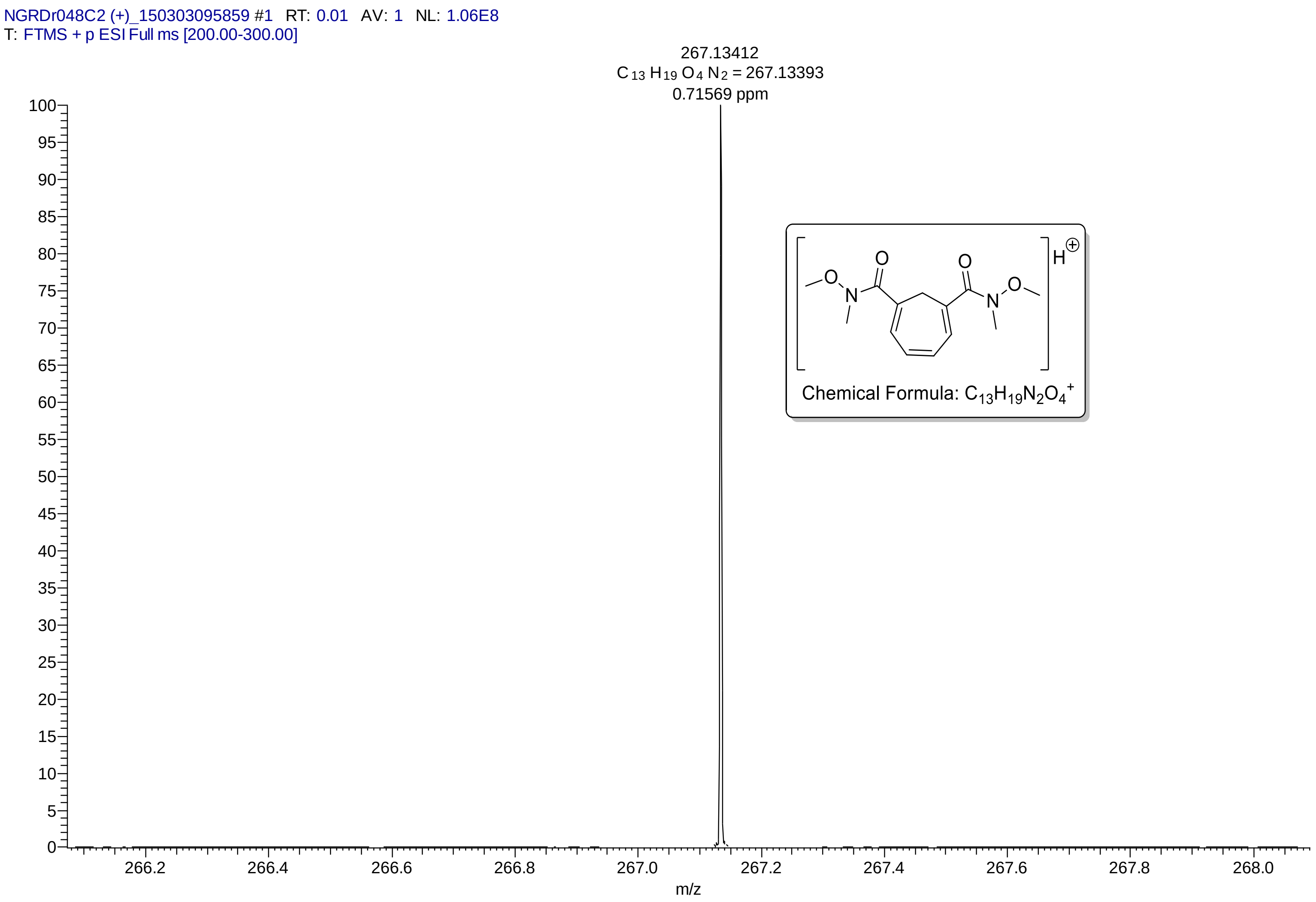

Figure S27. HRMS—ESI-TOF—compound **5**.

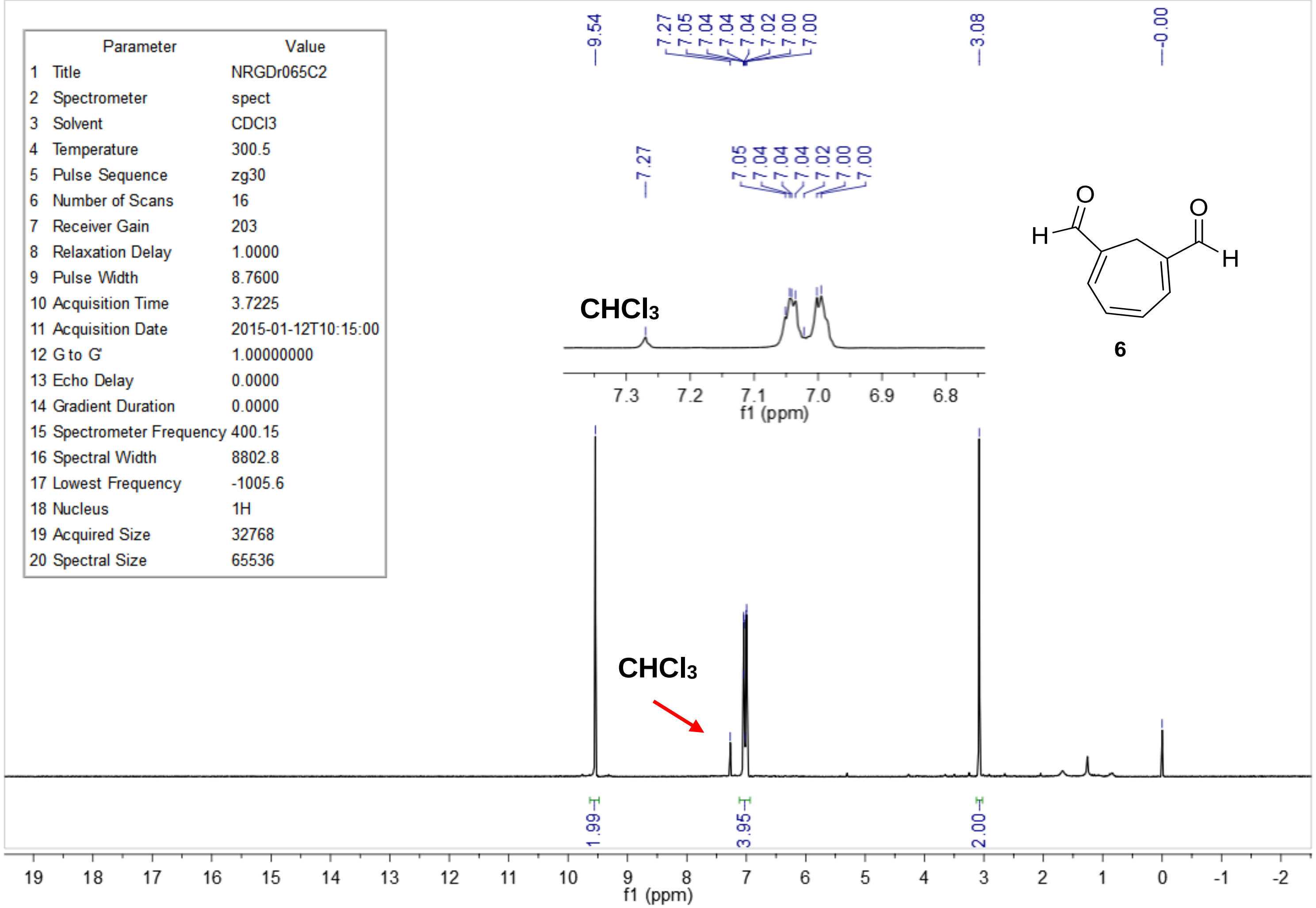

Figure S28. ^{1}H-NMR (400 MHz)($CDCl_3$)—compound **6**.

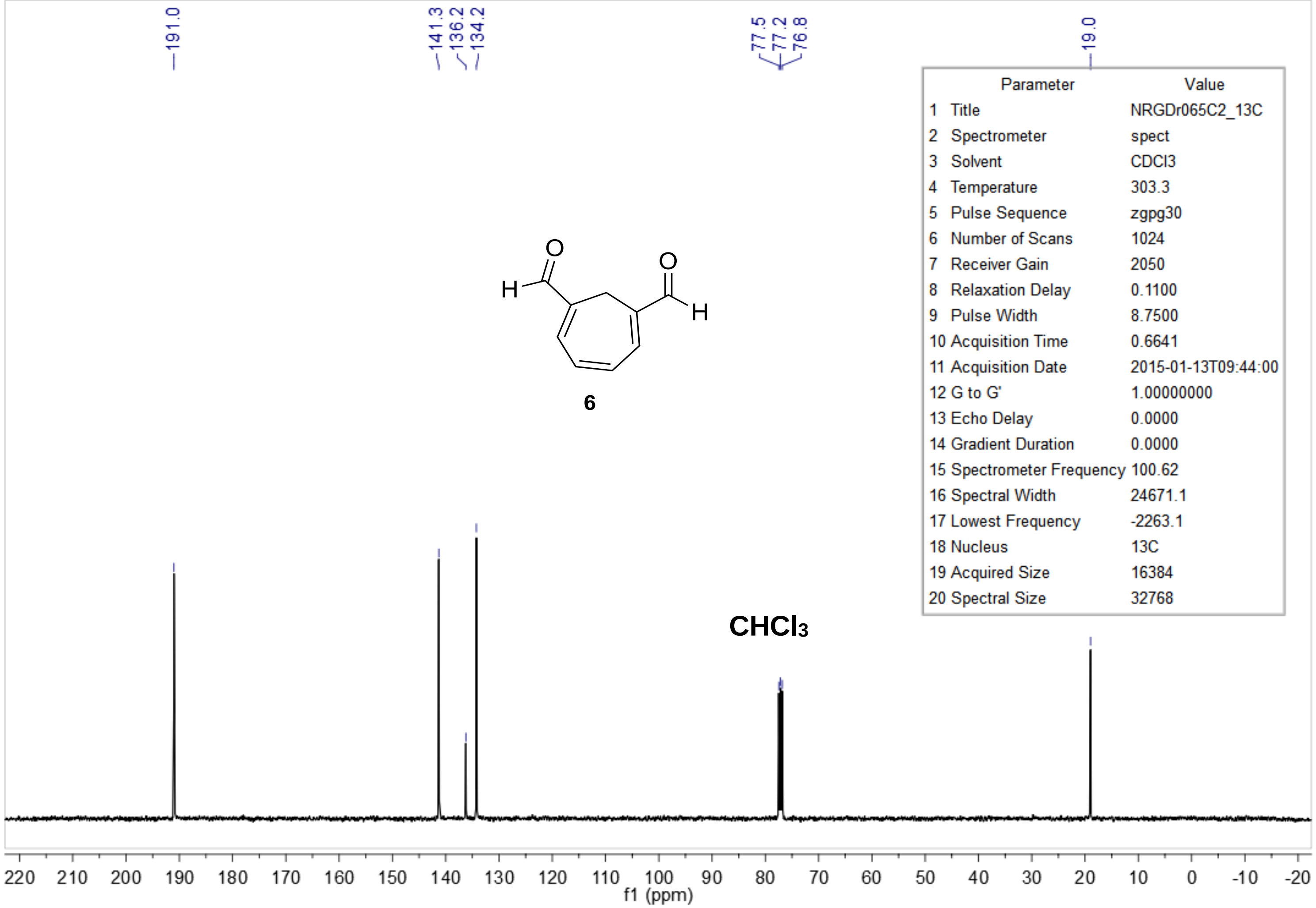

Figure S29. ^{13}C {^1H} (100 MHz)—compound **6**.

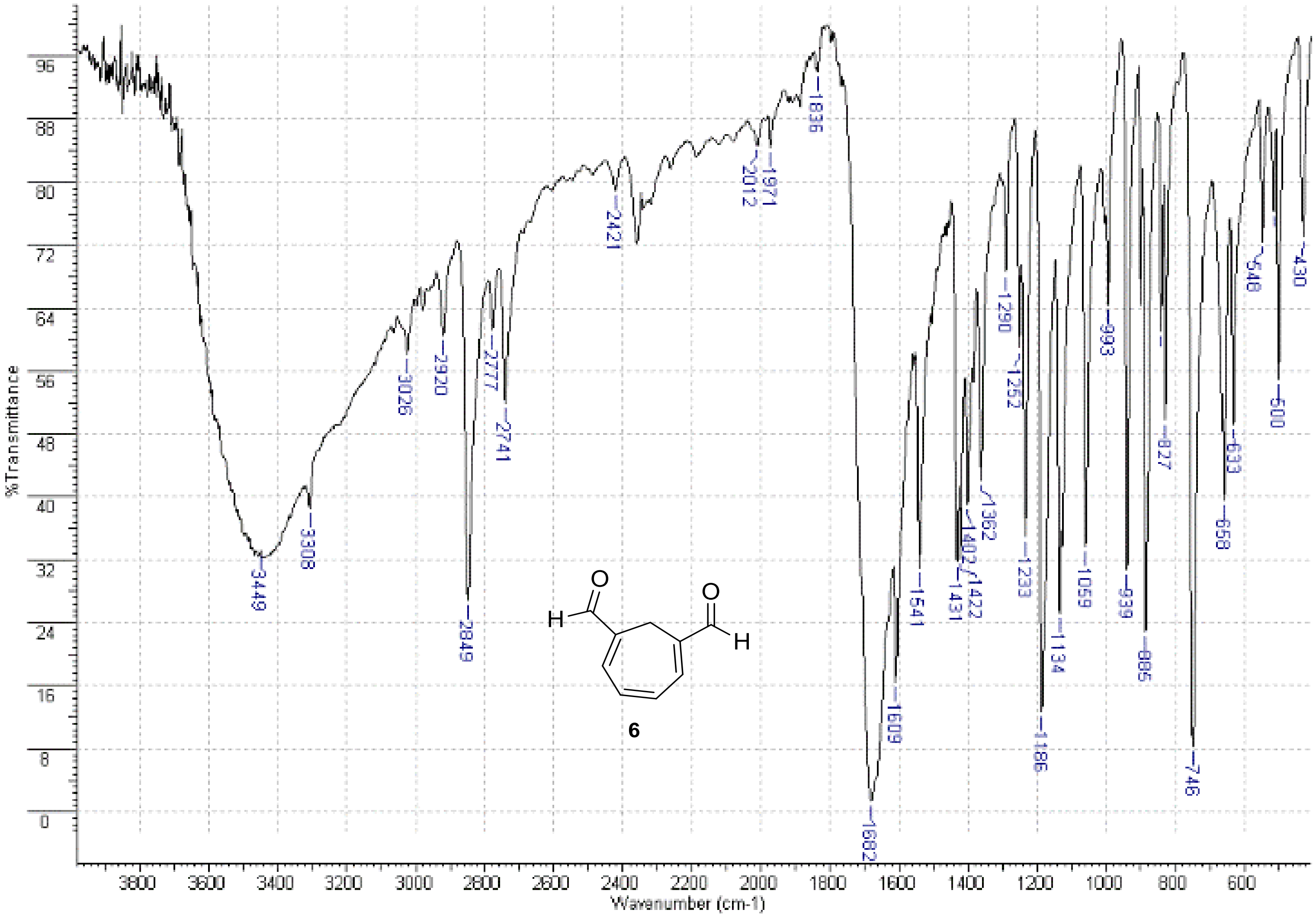

Figure S30. IR (KBr pellet)—compound **6**.

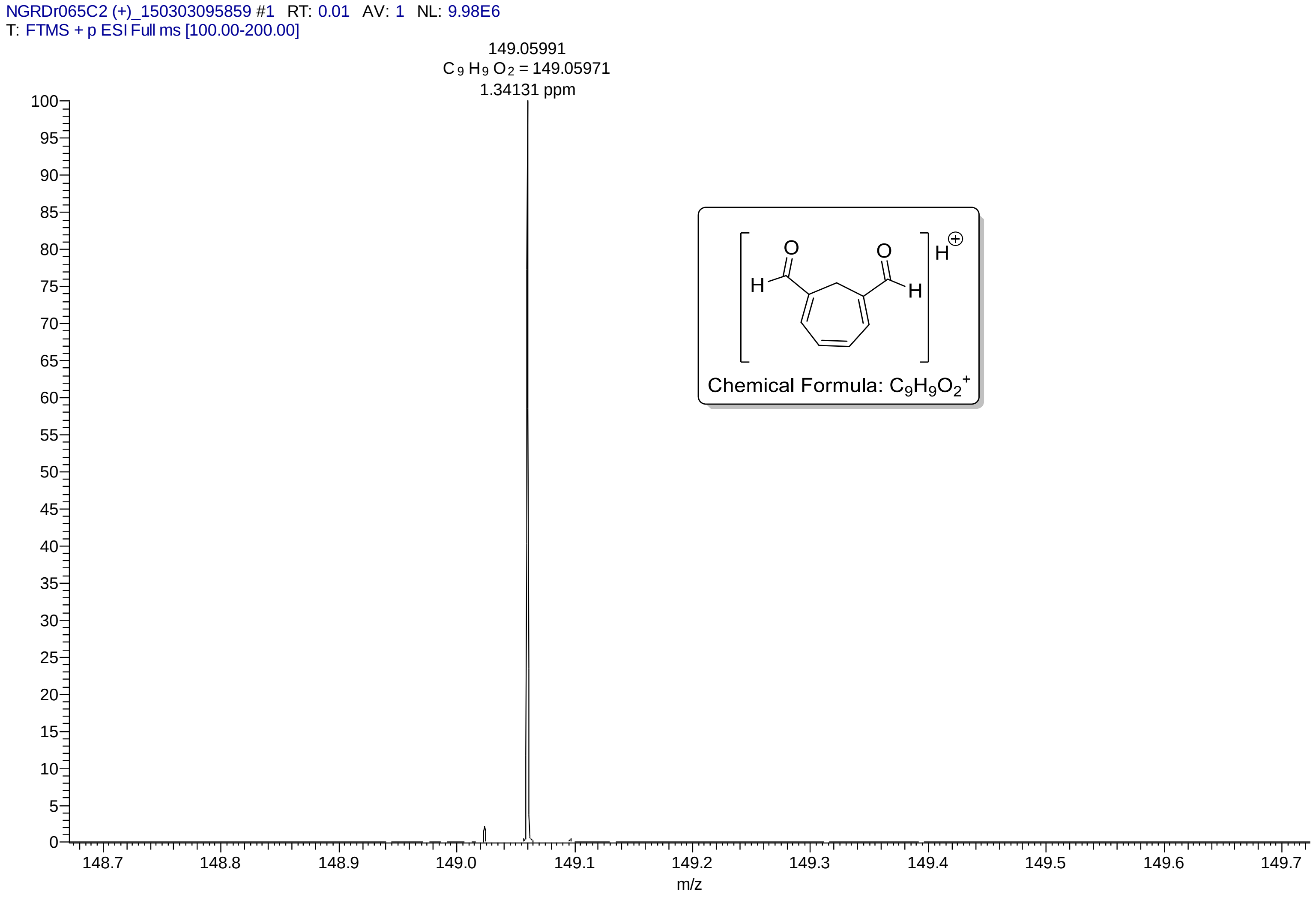

Figure S31. HRMS—ESI-TOF—compound **6**.

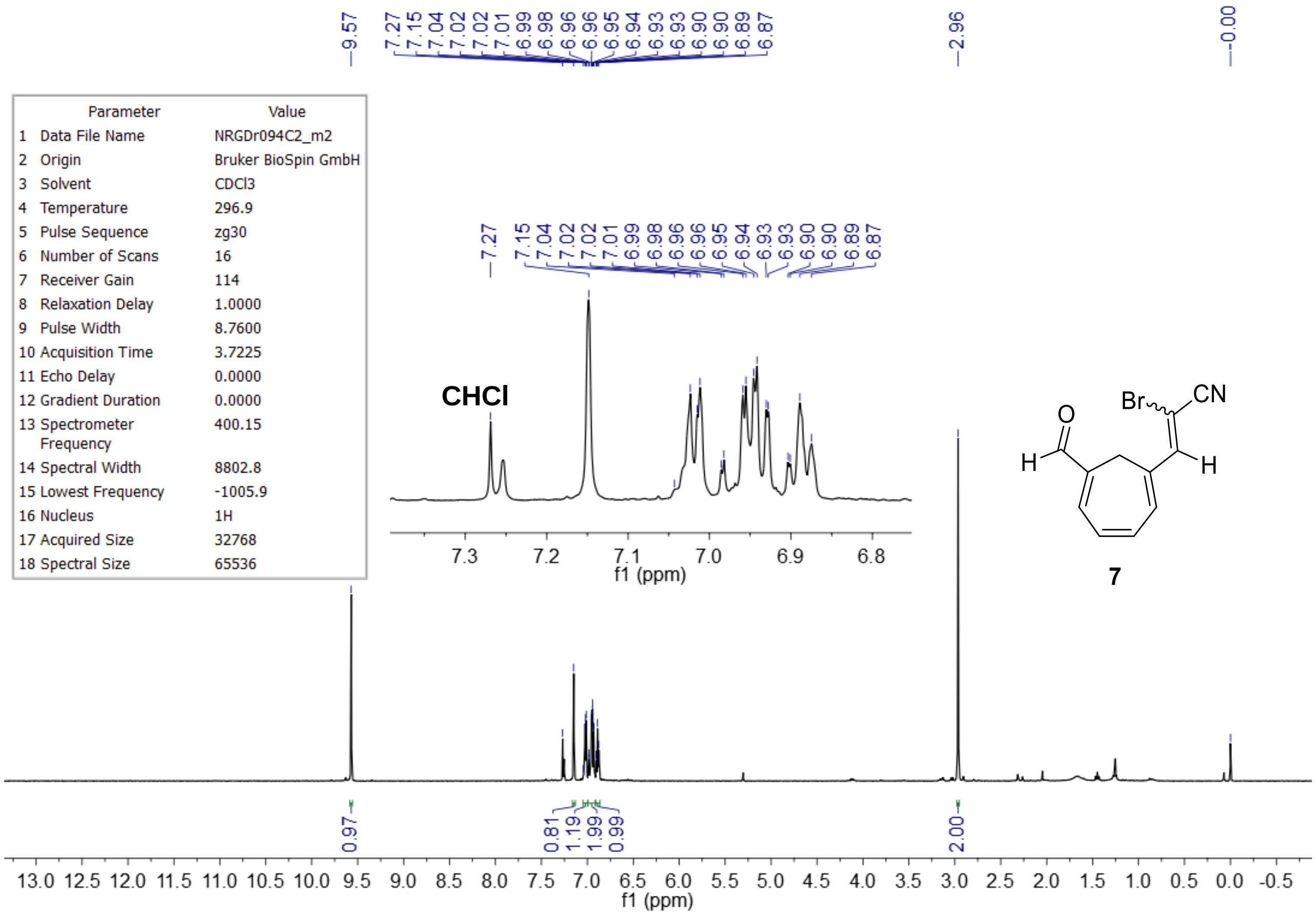

Figure S32. ^{1}H-NMR (400 MHz) ($CDCl_3$)—compound **7**.

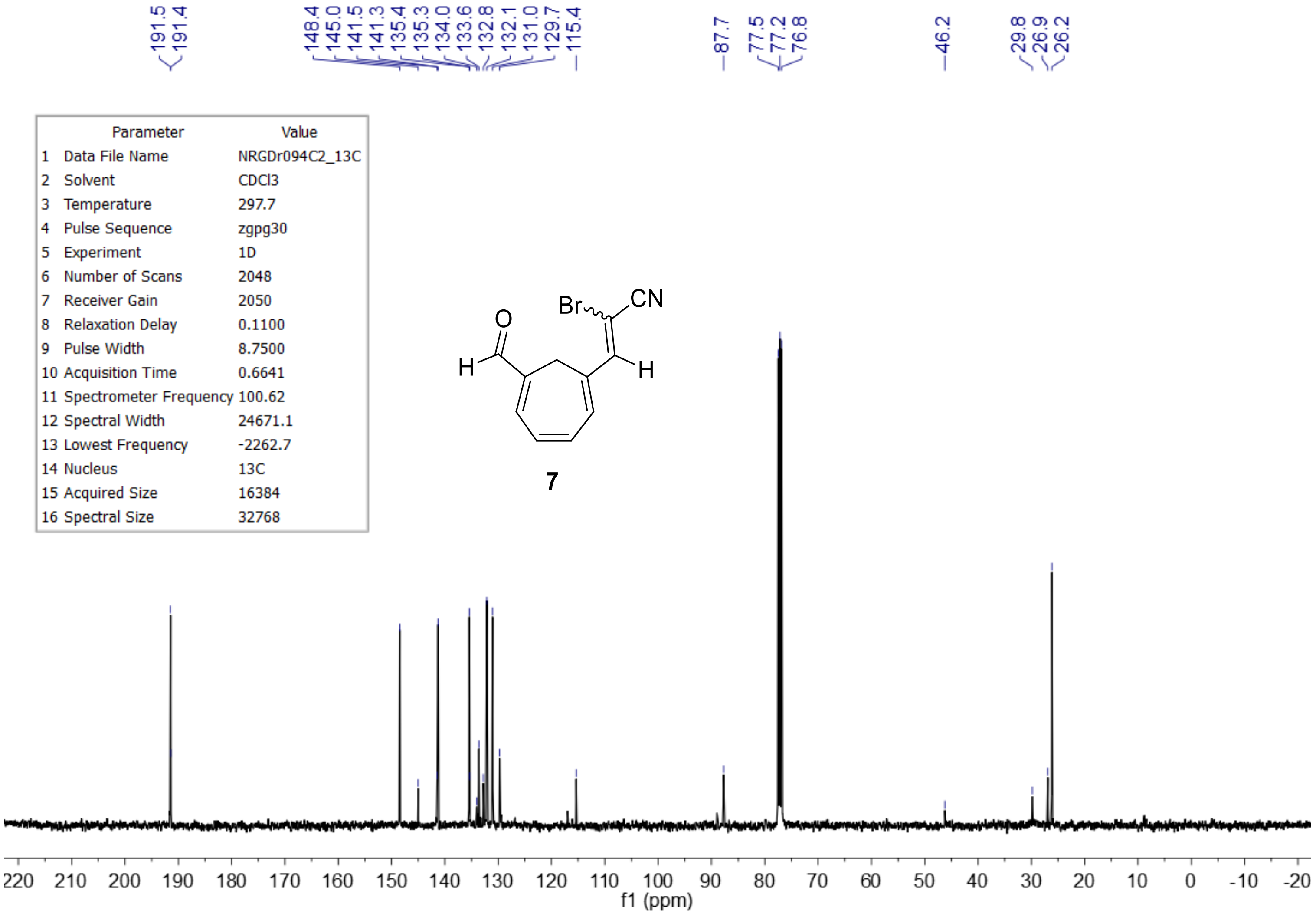

Figure S33. $^{13}C\ \{^1H\}$ (100 MHz) ($CDCl_3$)—compound **7**.

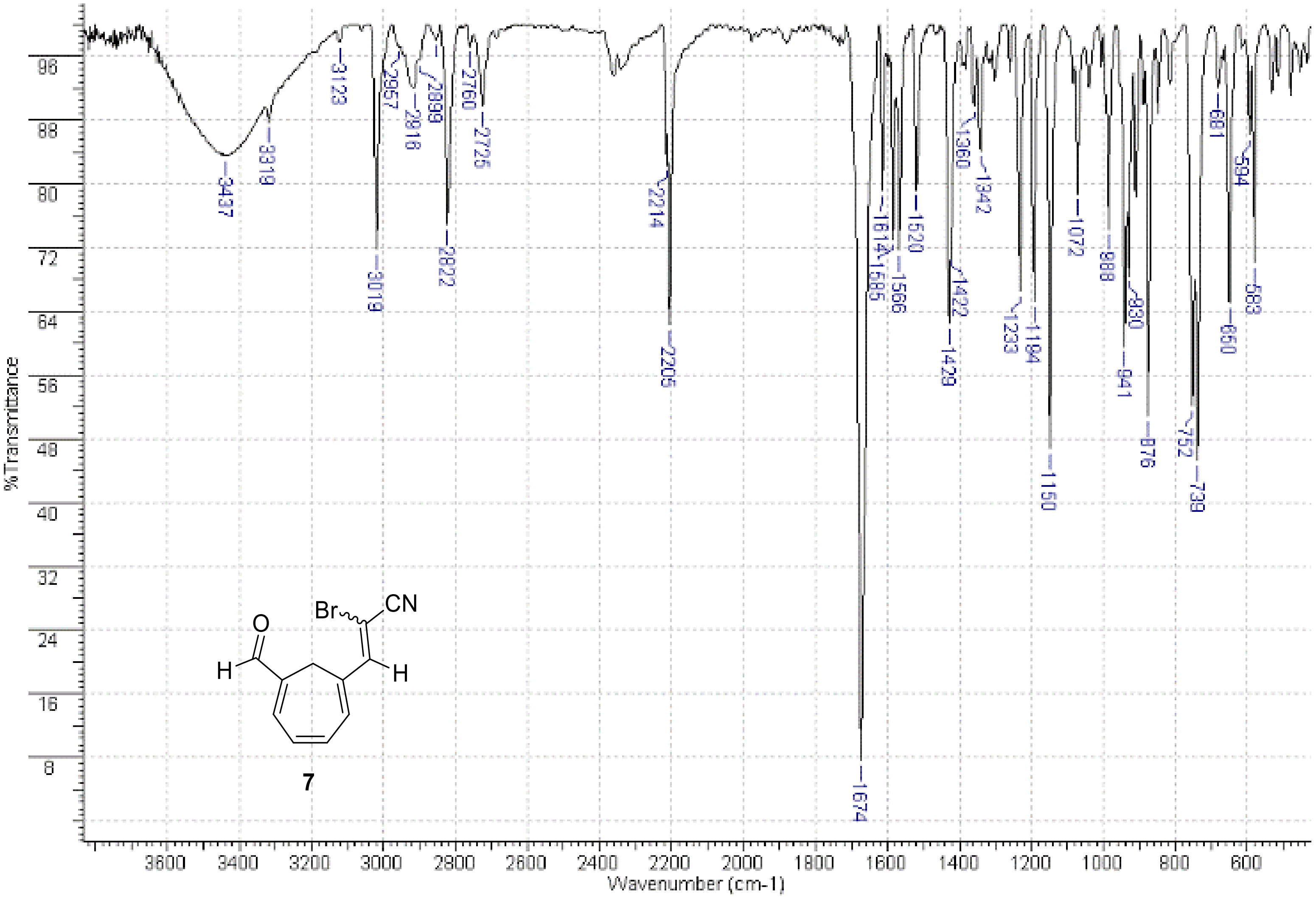

Figure S34. IR (KBr pellet)—compound **7**.

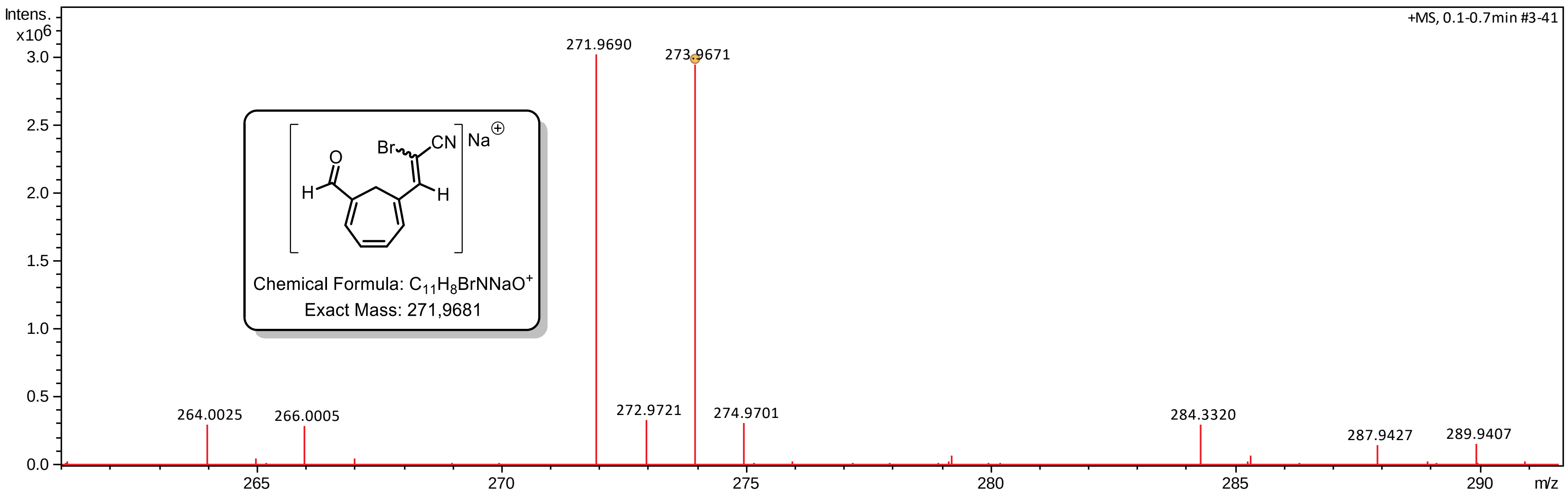

Figure S35. HRMS—ESI-TOF—compound **7**.

Meas. *m*/*z*	Ion Formula	*m*/*z*	Err [ppm]	mSigma	Score	rdb	Adduct
271.9690	$C_{11}H_8BrNNaO$	271.9681	–3.3	12.8	100.00	7.5	M+Na

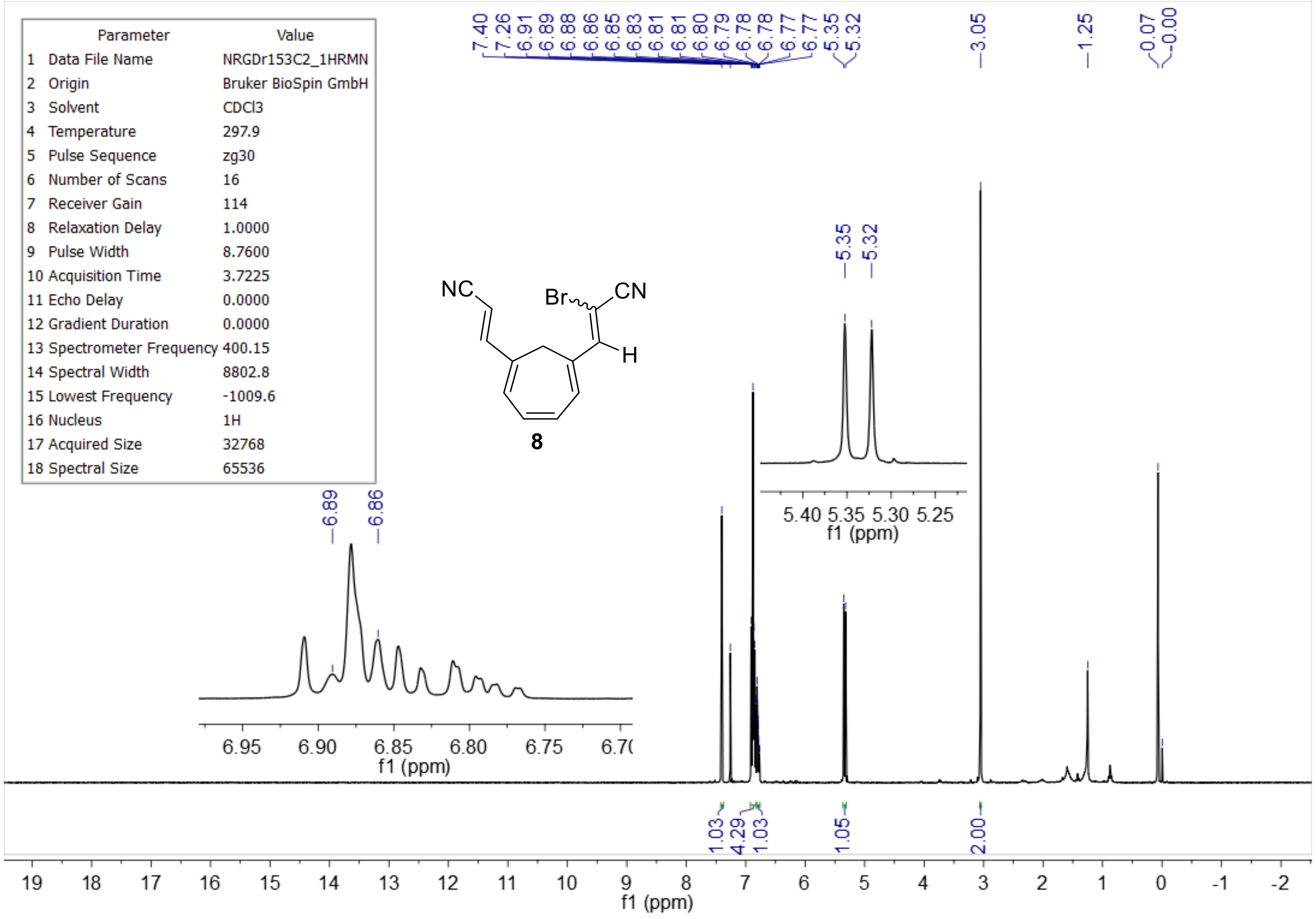

Figure S36. ^{1}H-NMR (400 MHz) ($CDCl_3$)—compound **8**.

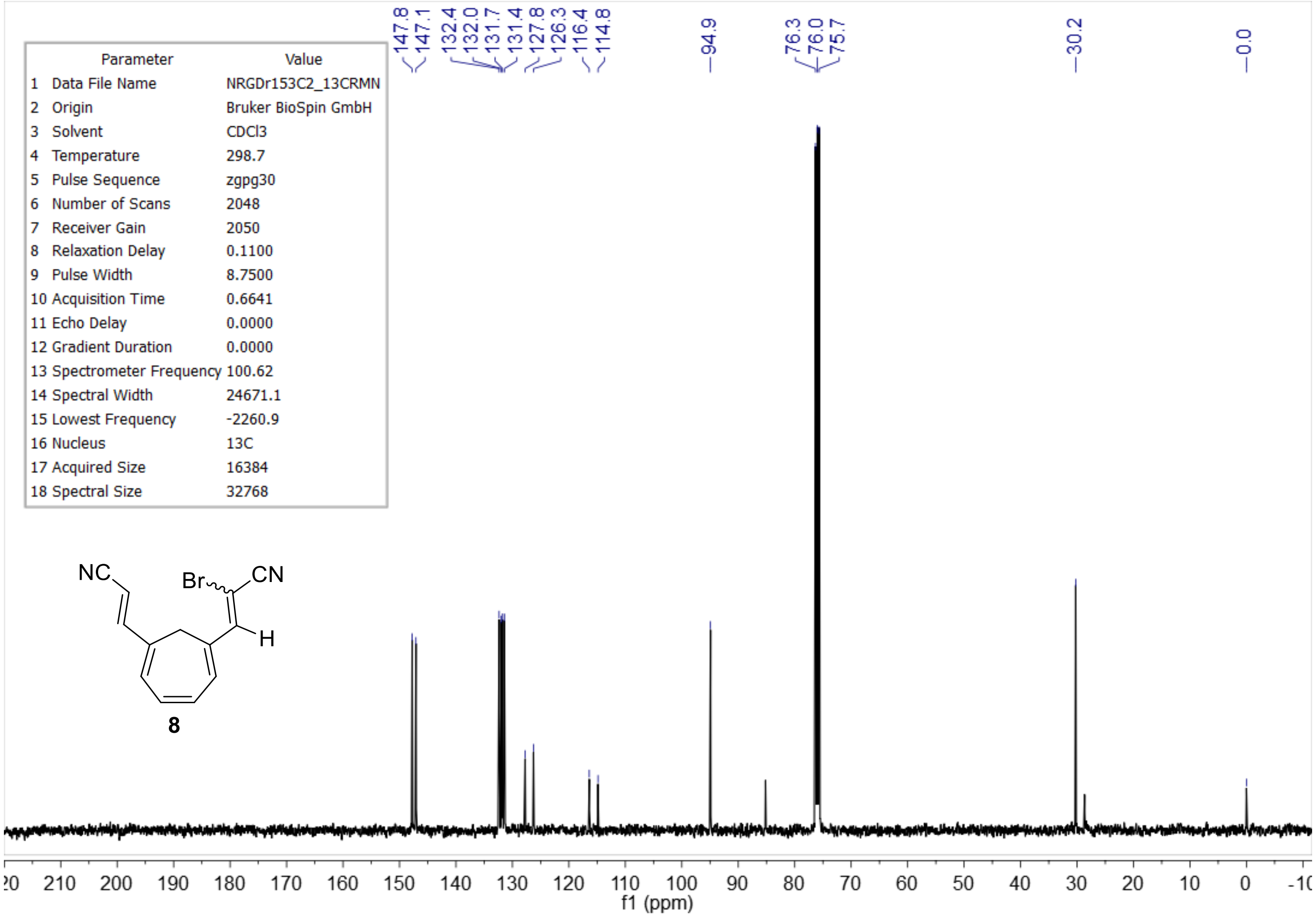

	Parameter	Value
1	Data File Name	NRGDr153C2_13CRMN
2	Origin	Bruker BioSpin GmbH
3	Solvent	CDCl3
4	Temperature	298.7
5	Pulse Sequence	zgpg30
6	Number of Scans	2048
7	Receiver Gain	2050
8	Relaxation Delay	0.1100
9	Pulse Width	8.7500
10	Acquisition Time	0.6641
11	Echo Delay	0.0000
12	Gradient Duration	0.0000
13	Spectrometer Frequency	100.62
14	Spectral Width	24671.1
15	Lowest Frequency	-2260.9
16	Nucleus	13C
17	Acquired Size	16384
18	Spectral Size	32768

Figure S37. ^{13}C {^1H} (100 MHz) ($CDCl_3$)—compound **8**.

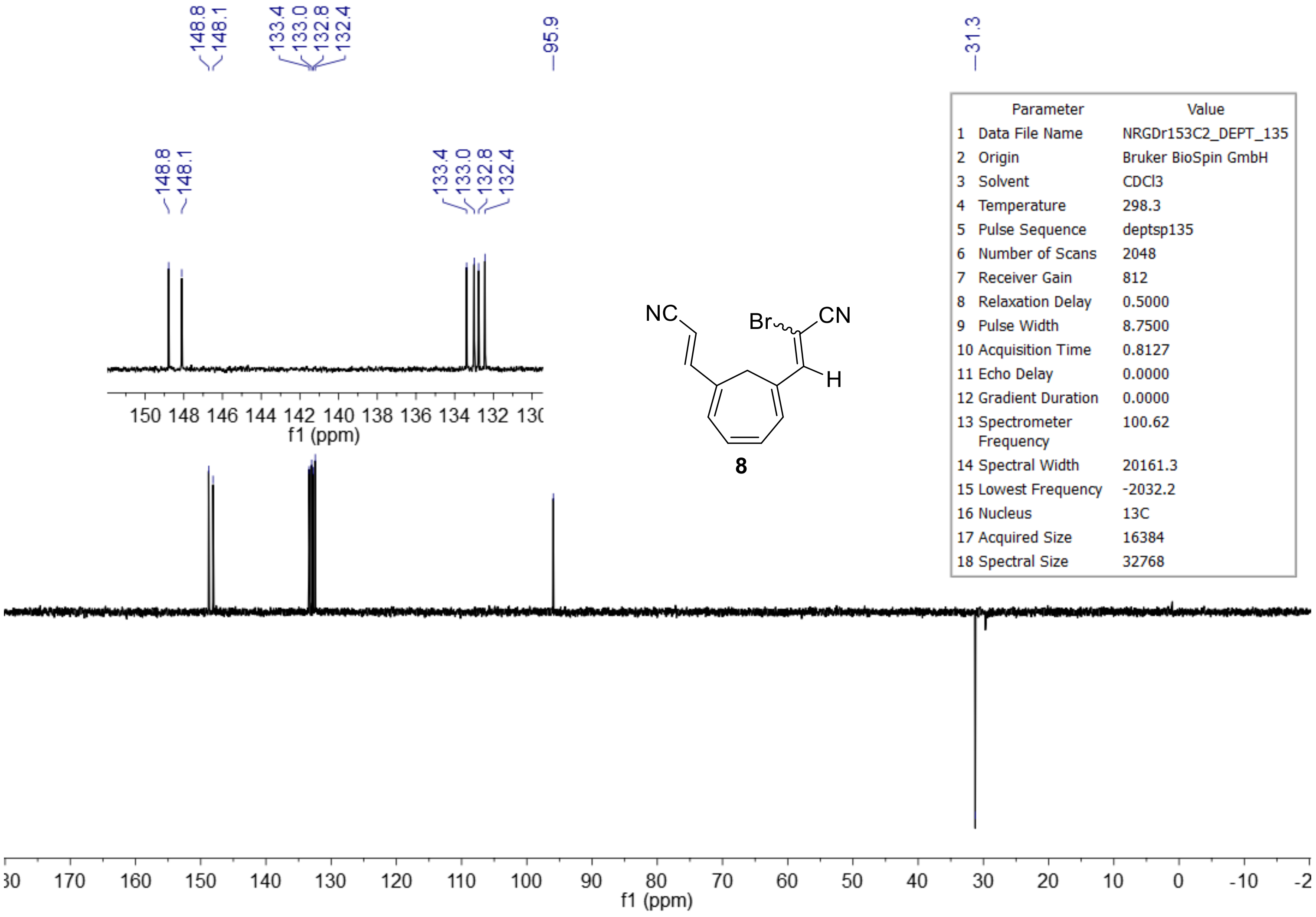

	Parameter	Value
1	Data File Name	NRGDr153C2_DEPT_135
2	Origin	Bruker BioSpin GmbH
3	Solvent	CDCl3
4	Temperature	298.3
5	Pulse Sequence	deptsp135
6	Number of Scans	2048
7	Receiver Gain	812
8	Relaxation Delay	0.5000
9	Pulse Width	8.7500
10	Acquisition Time	0.8127
11	Echo Delay	0.0000
12	Gradient Duration	0.0000
13	Spectrometer Frequency	100.62
14	Spectral Width	20161.3
15	Lowest Frequency	-2032.2
16	Nucleus	13C
17	Acquired Size	16384
18	Spectral Size	32768

Figure S38. ^{13}C (DEPT-135) ($CDCl_3$)—compound **8**.

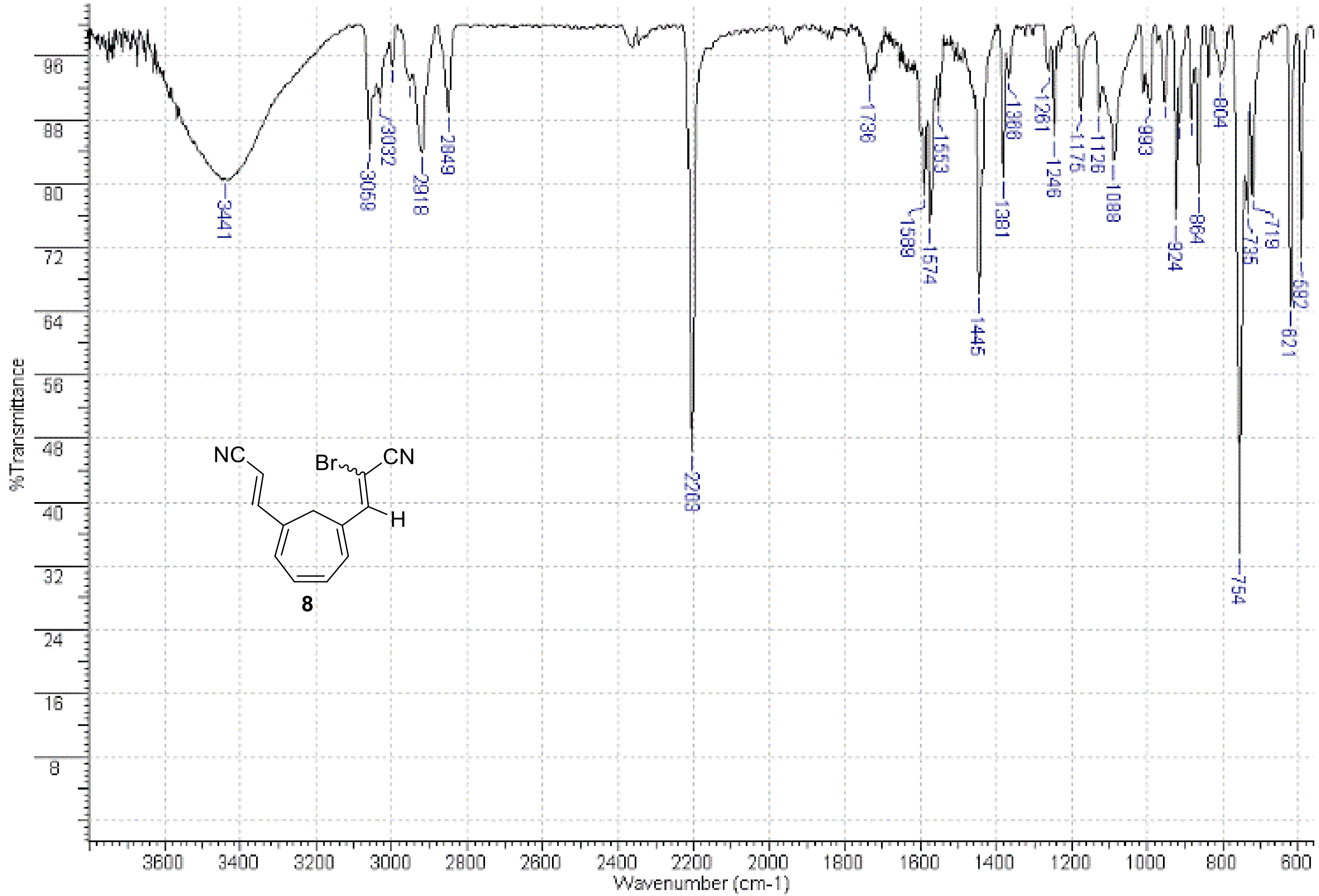

Figure S39. IR (KBr pellet)—compound **8**.

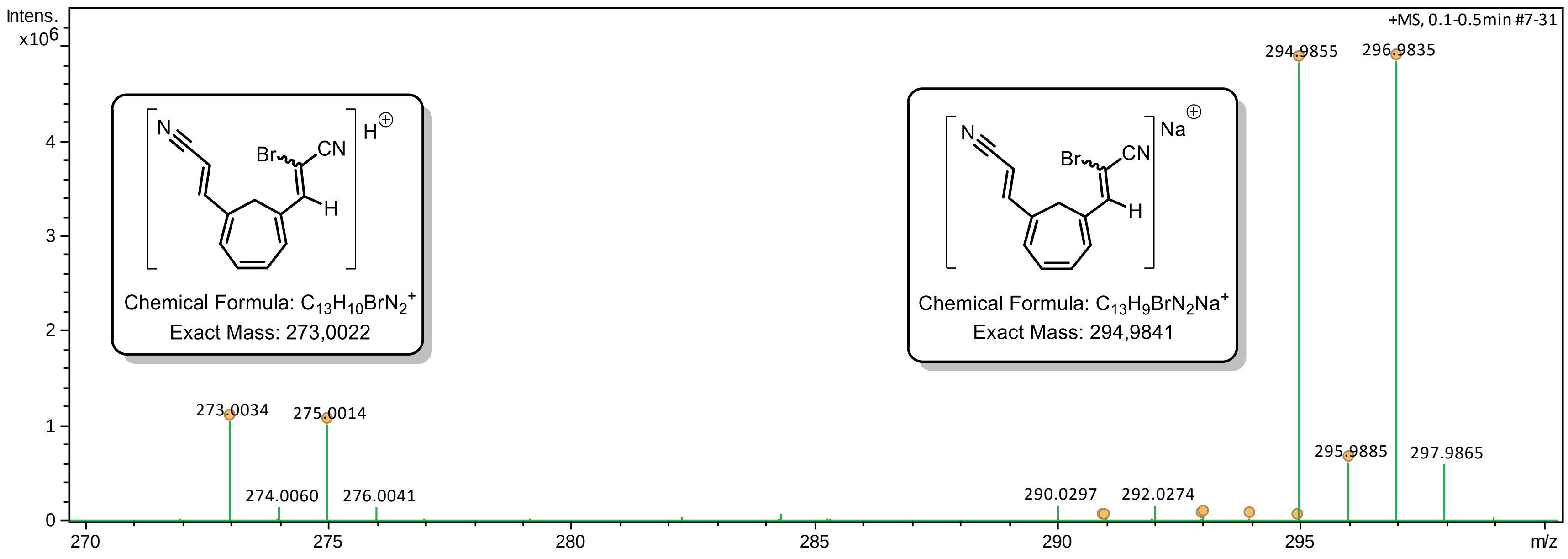

Figure S40. HRMS—ESI-TOF—compound **8**.

Meas. *m/z*	Ion Formula	*m/z*	Err [ppm]	mSigma	Score	rdb	Adduct
273.0034	$C_{13}H_{10}BrN_2$	273.0022	–4.4	12.6	100.00	9.5	M+H
294.9855	$C_{13}H_9BrN_2Na$	294.9841	–4.5	18.5	100.0	9.5	M+Na

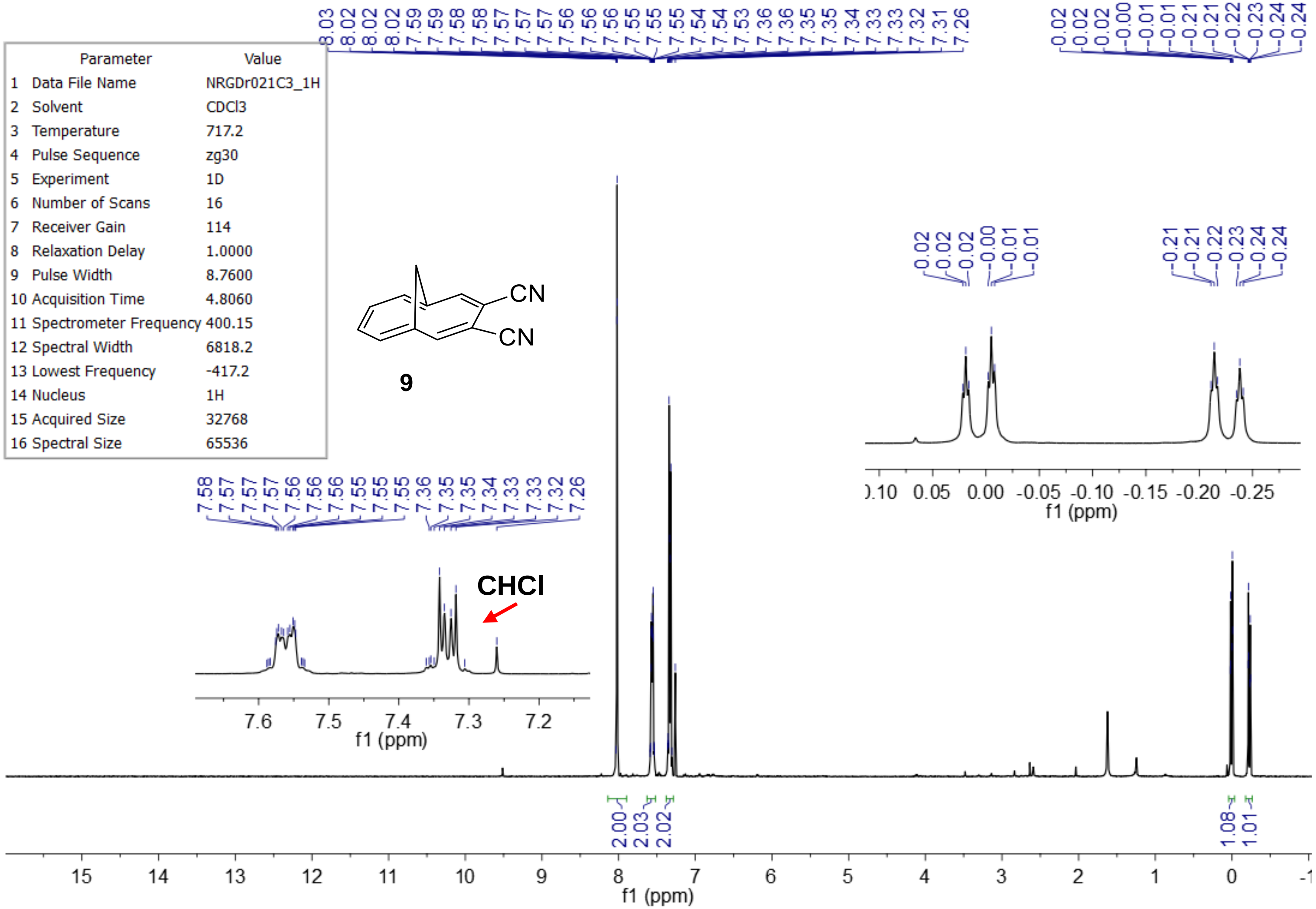

Figure S41. ^{1}H-NMR (400 MHz) ($CDCl_3$)—compound **9**.

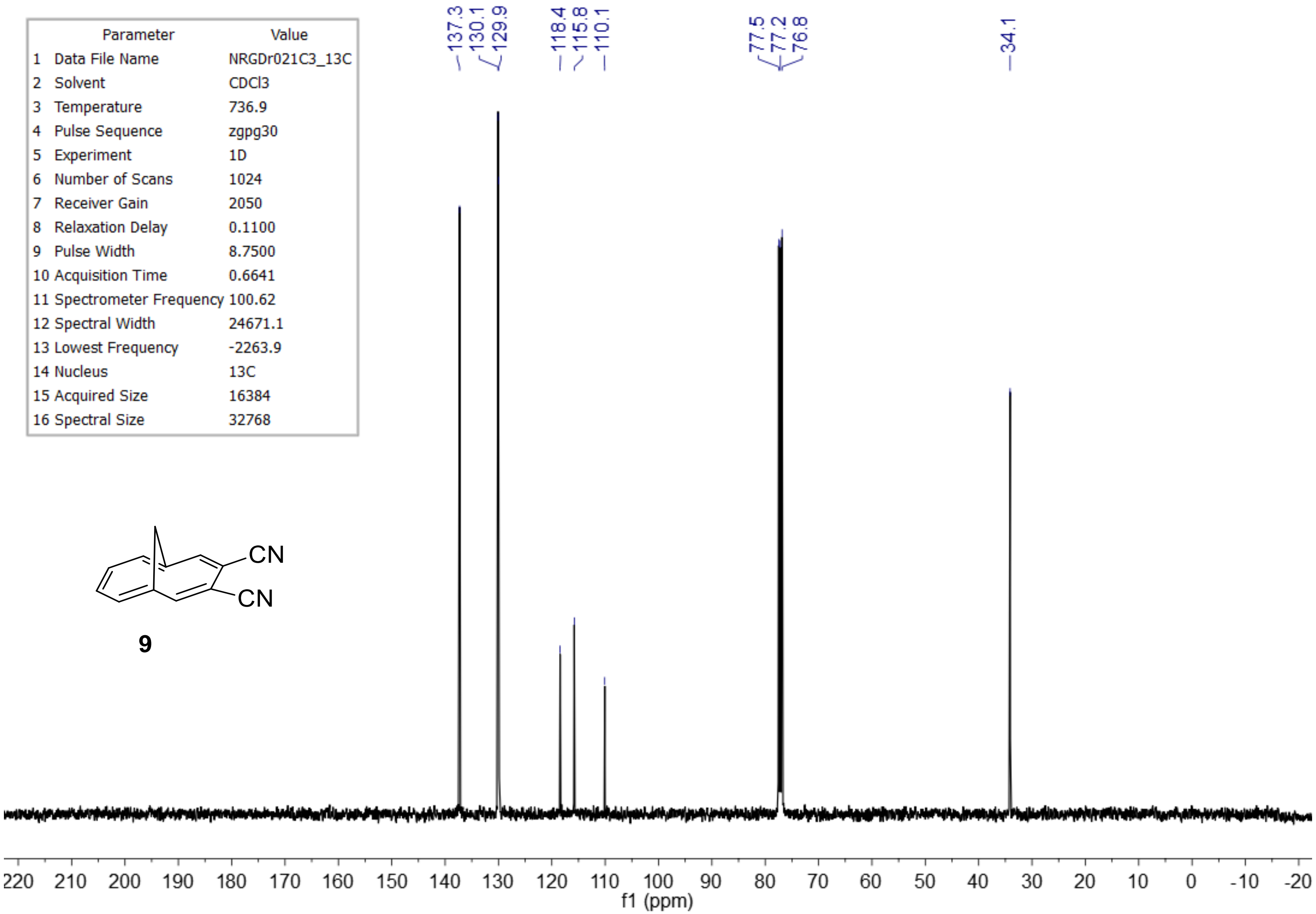

Figure S42. $^{13}C\{^1H\}$ (100 MHz) ($CDCl_3$)—compound **9**.

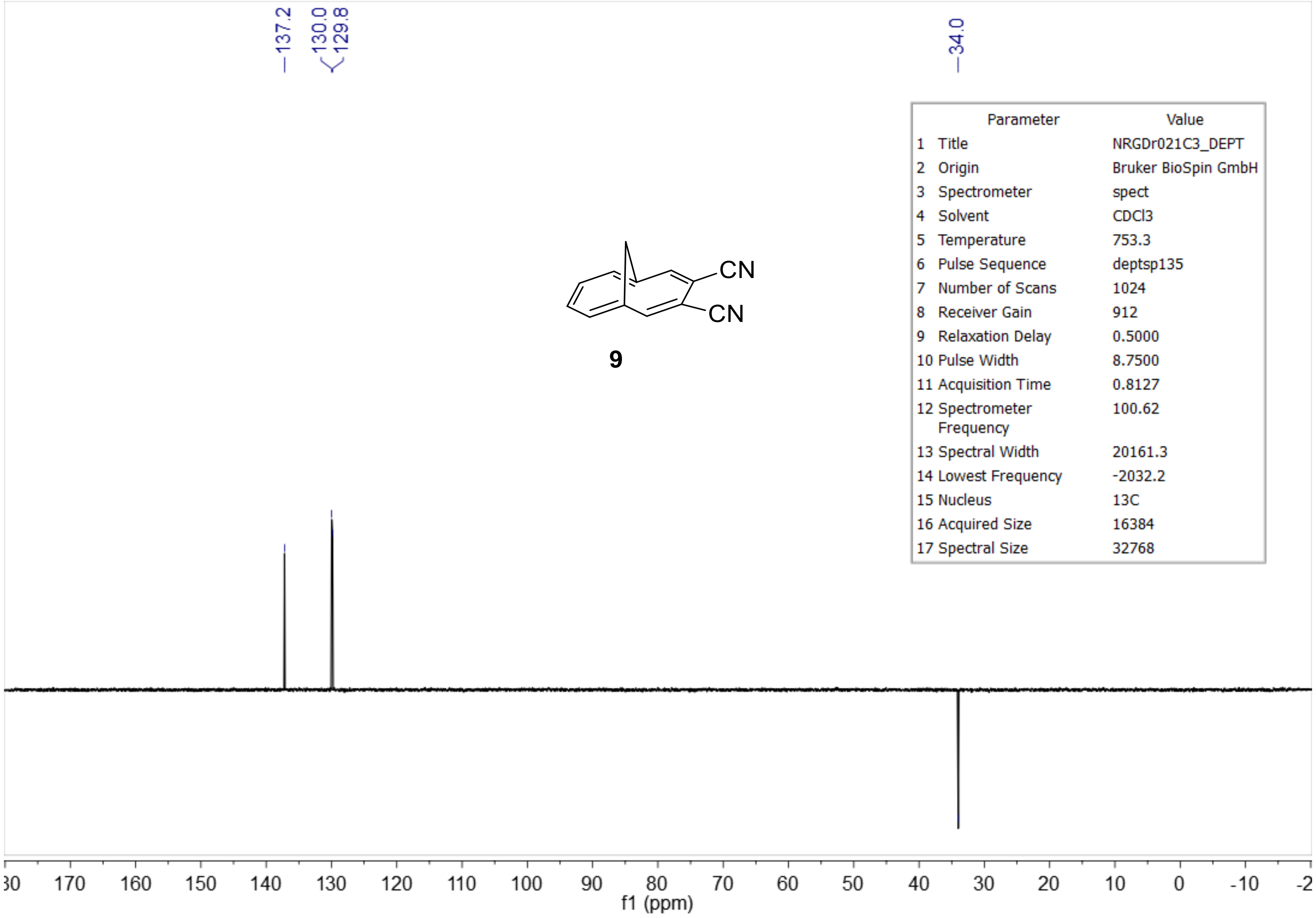

	Parameter	Value
1	Title	NRGDr021C3_DEPT
2	Origin	Bruker BioSpin GmbH
3	Spectrometer	spect
4	Solvent	CDCl3
5	Temperature	753.3
6	Pulse Sequence	deptsp135
7	Number of Scans	1024
8	Receiver Gain	912
9	Relaxation Delay	0.5000
10	Pulse Width	8.7500
11	Acquisition Time	0.8127
12	Spectrometer Frequency	100.62
13	Spectral Width	20161.3
14	Lowest Frequency	-2032.2
15	Nucleus	13C
16	Acquired Size	16384
17	Spectral Size	32768

Figure S43. ^{13}C (DEPT-135) ($CDCl_3$)—compound **9**.

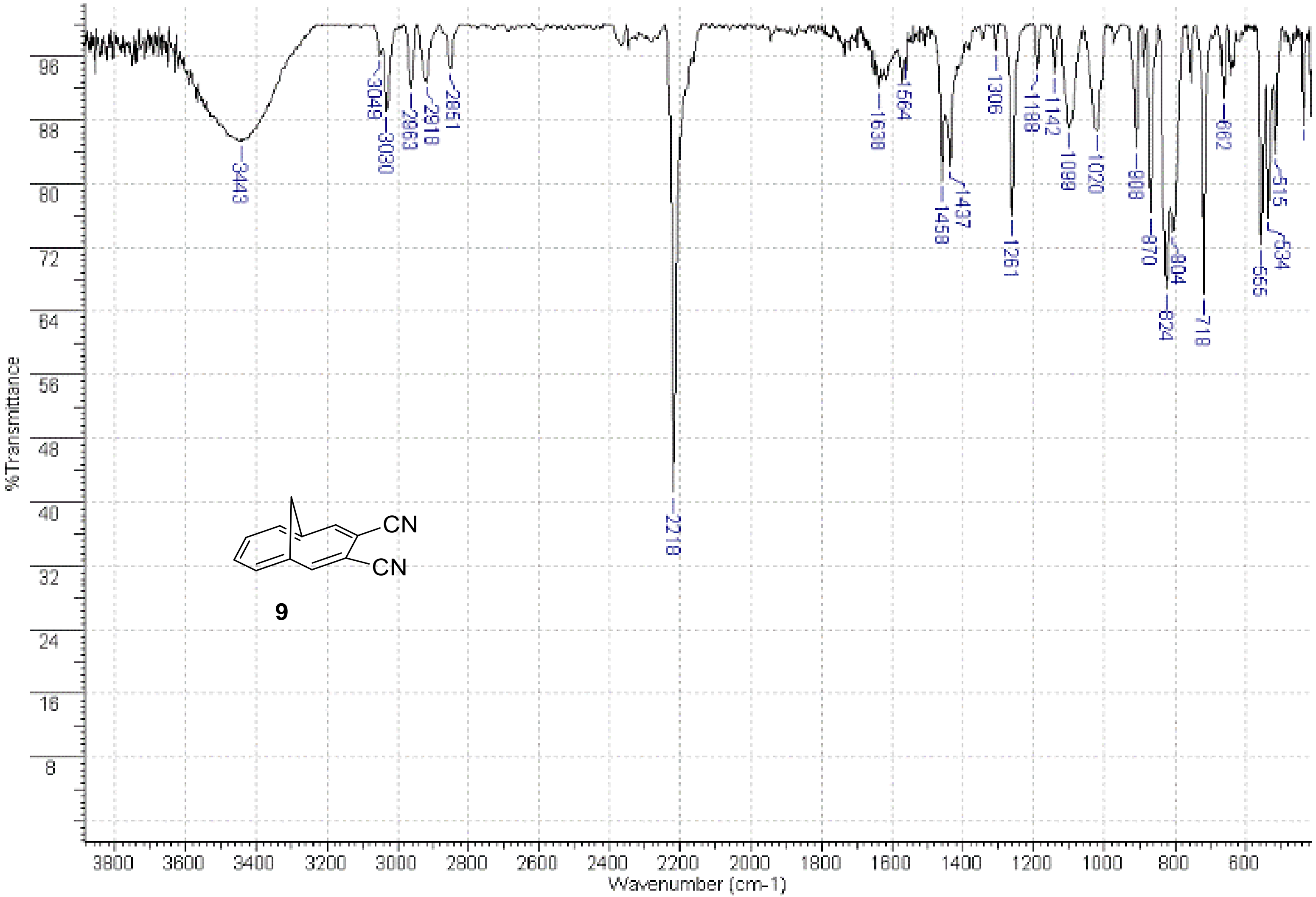

Figure S44. IR (KBr pellet)—compound **9**.

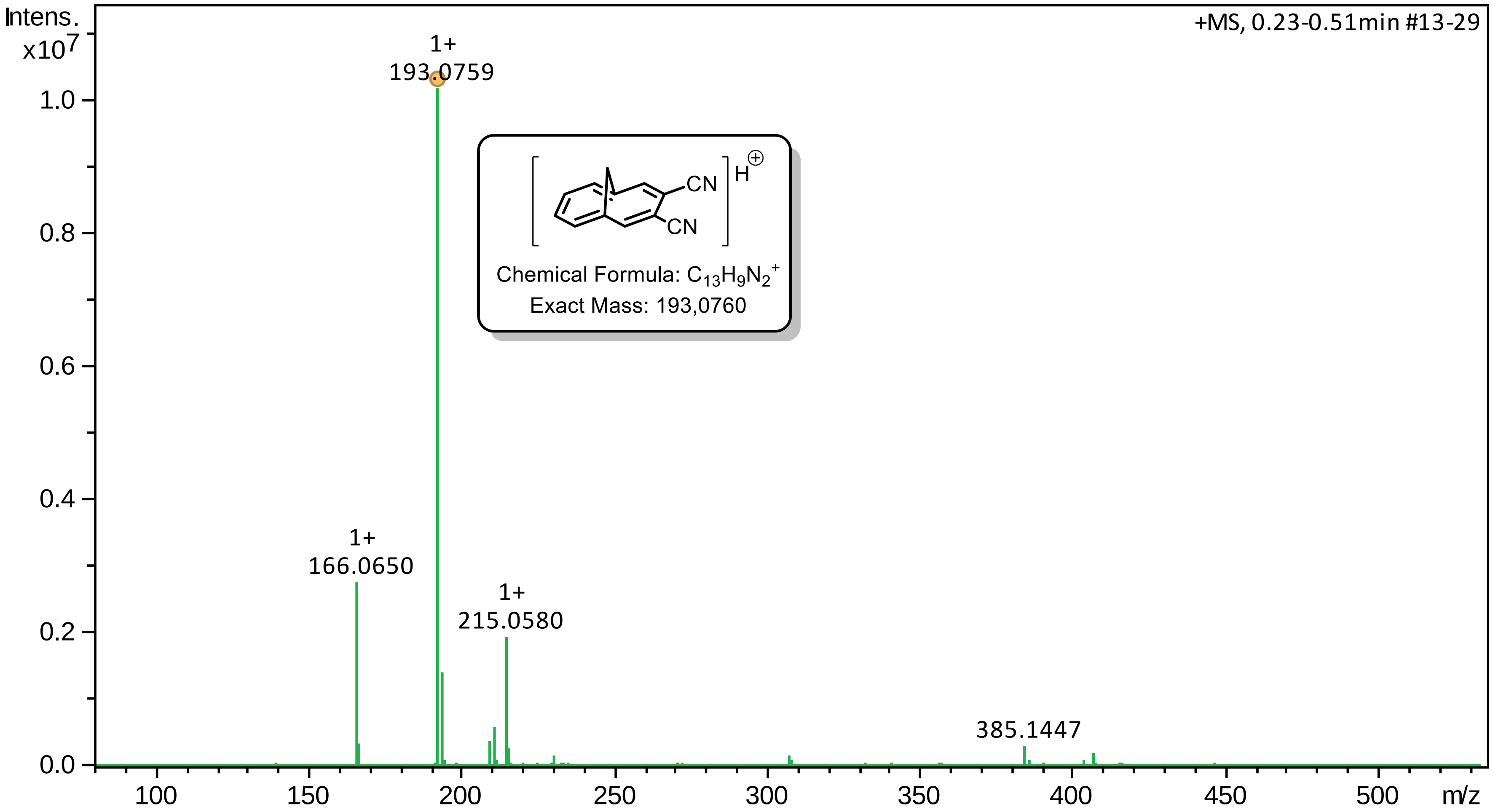

Figure S45. HRMS—ESI-TOF—compound **9**.

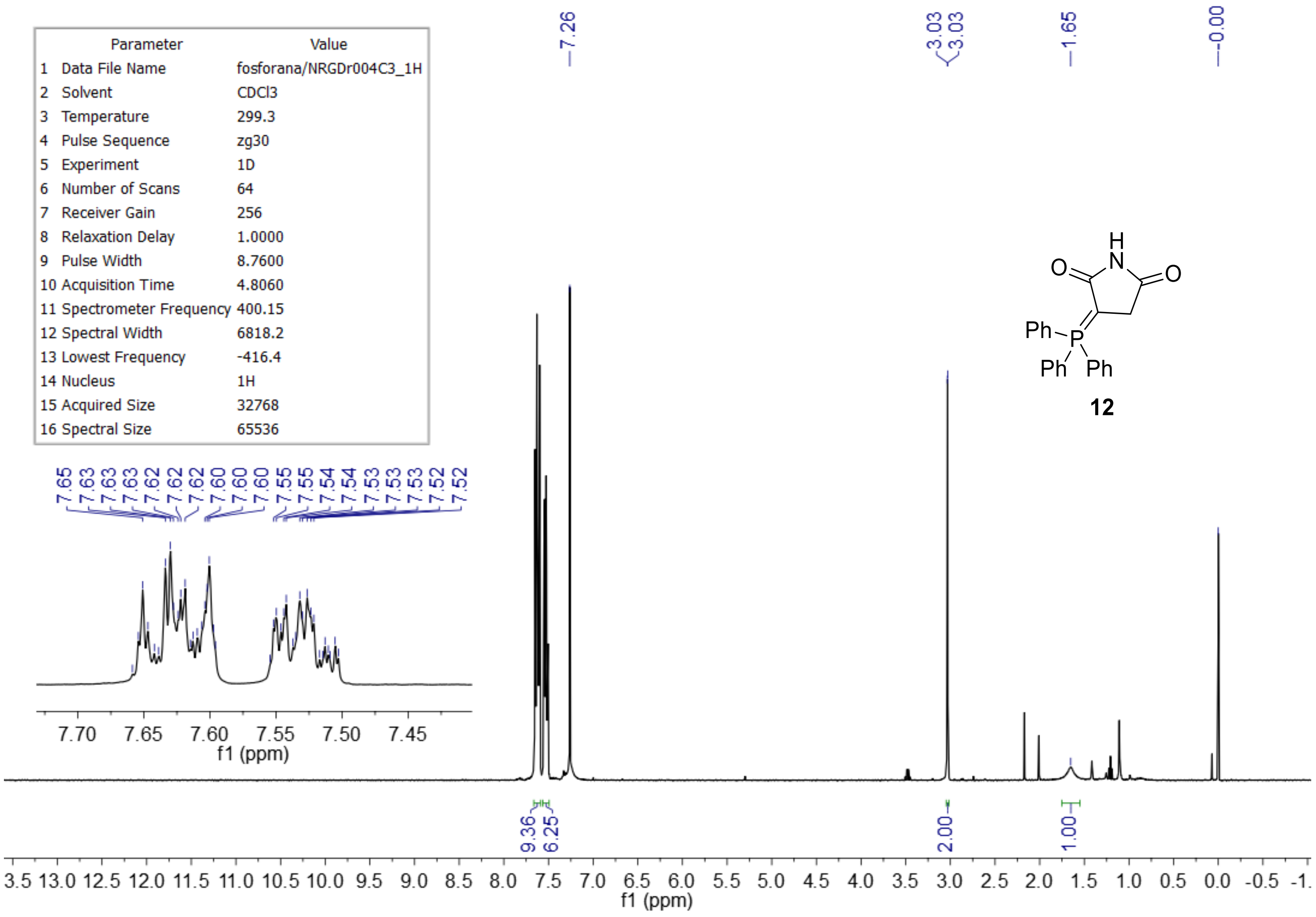

Figure S46. ^{1}H-NMR (400 MHz) ($CDCl_3$)—compound **12**.

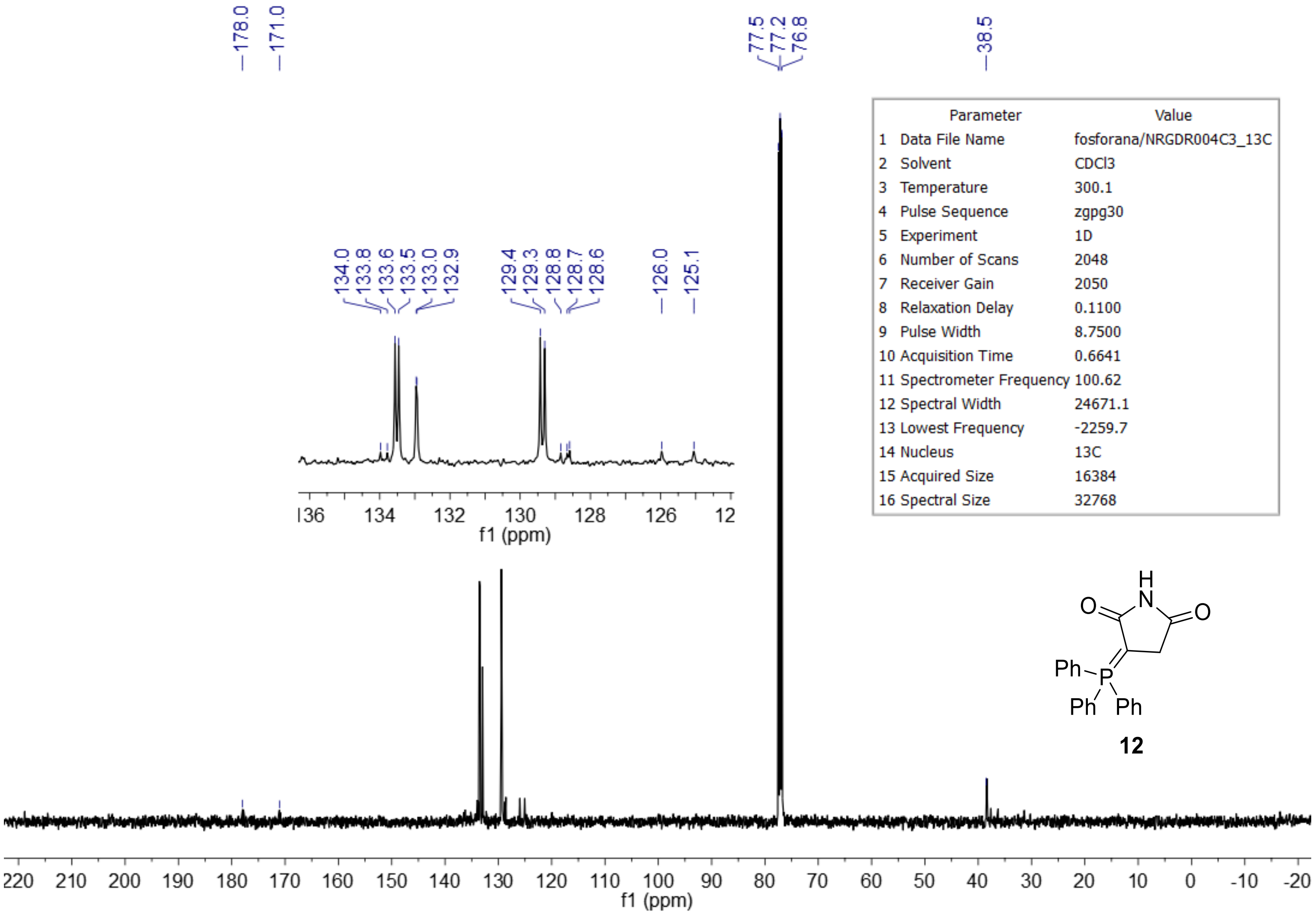

Figure S47. $^{13}C\ \{^1H\}$ (100 MHz) ($CDCl_3$)—compound **12**.

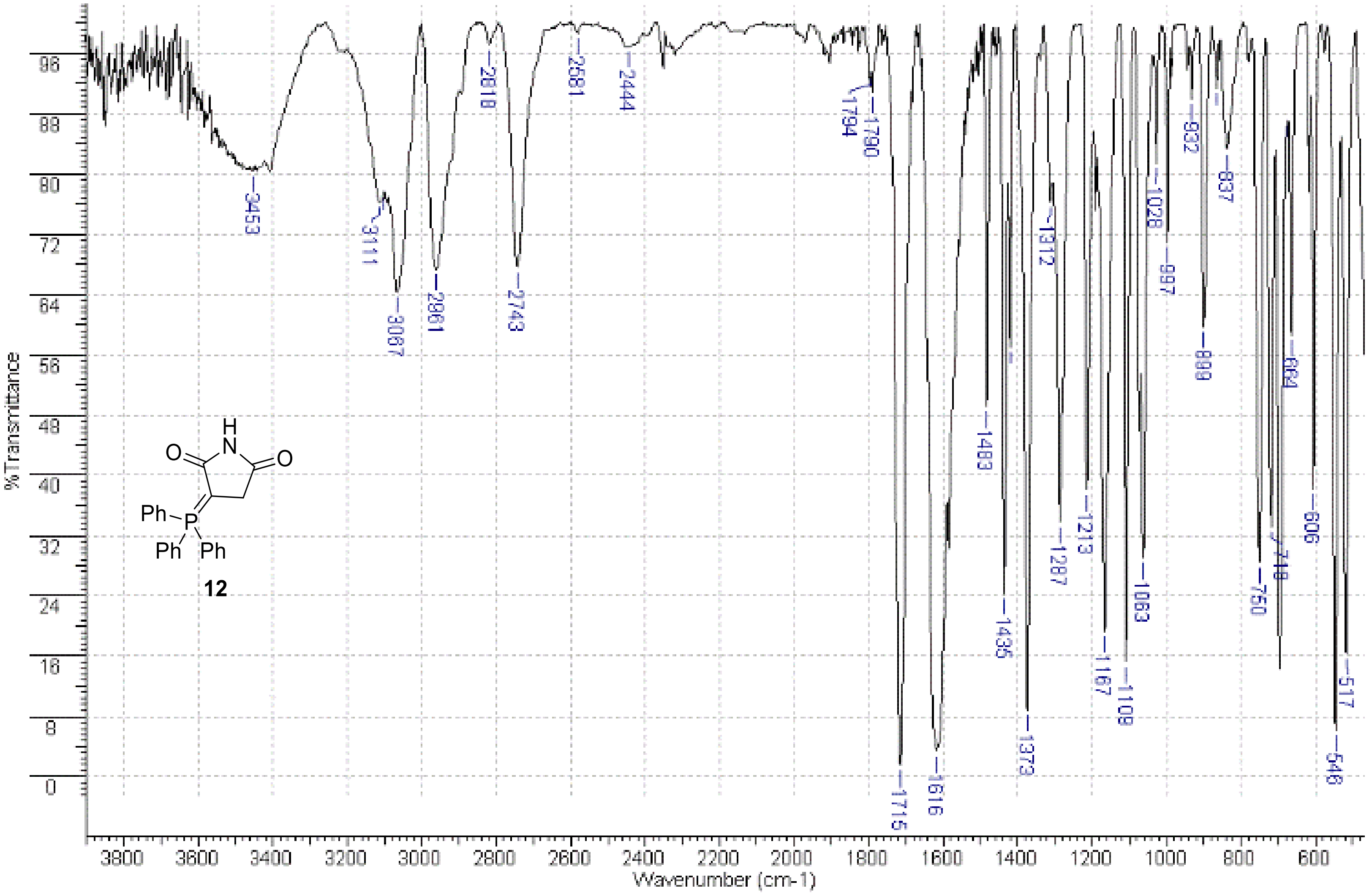

Figure S48. IR (KBr pellet)—compound **12**.

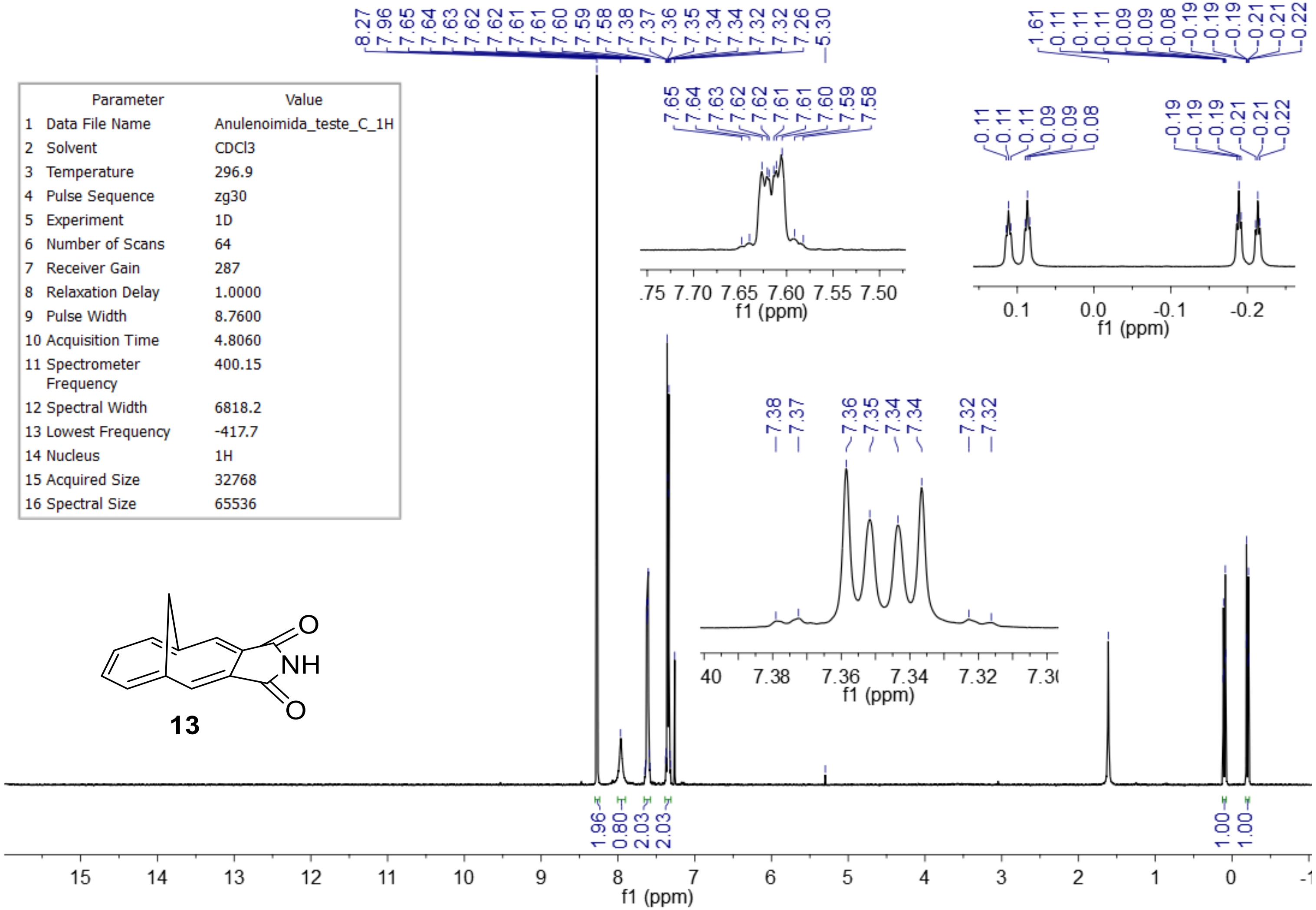

Figure S49. ^{1}H-NMR (400 MHz) ($CDCl_3$)—compound **13**.

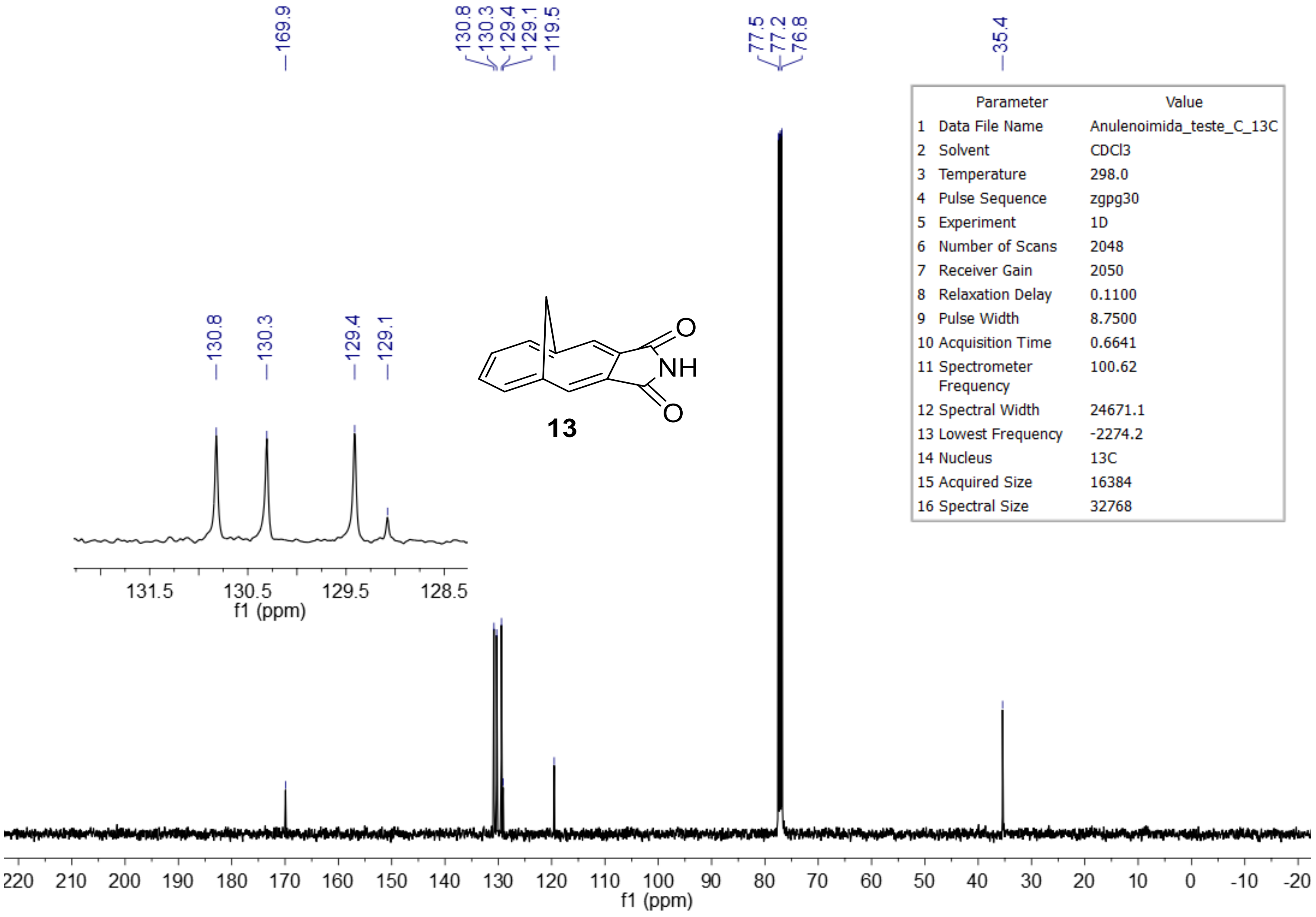

Figure S50. $^{13}C\ \{^{1}H\}$ (100 MHz) ($CDCl_3$)—compound **13**.

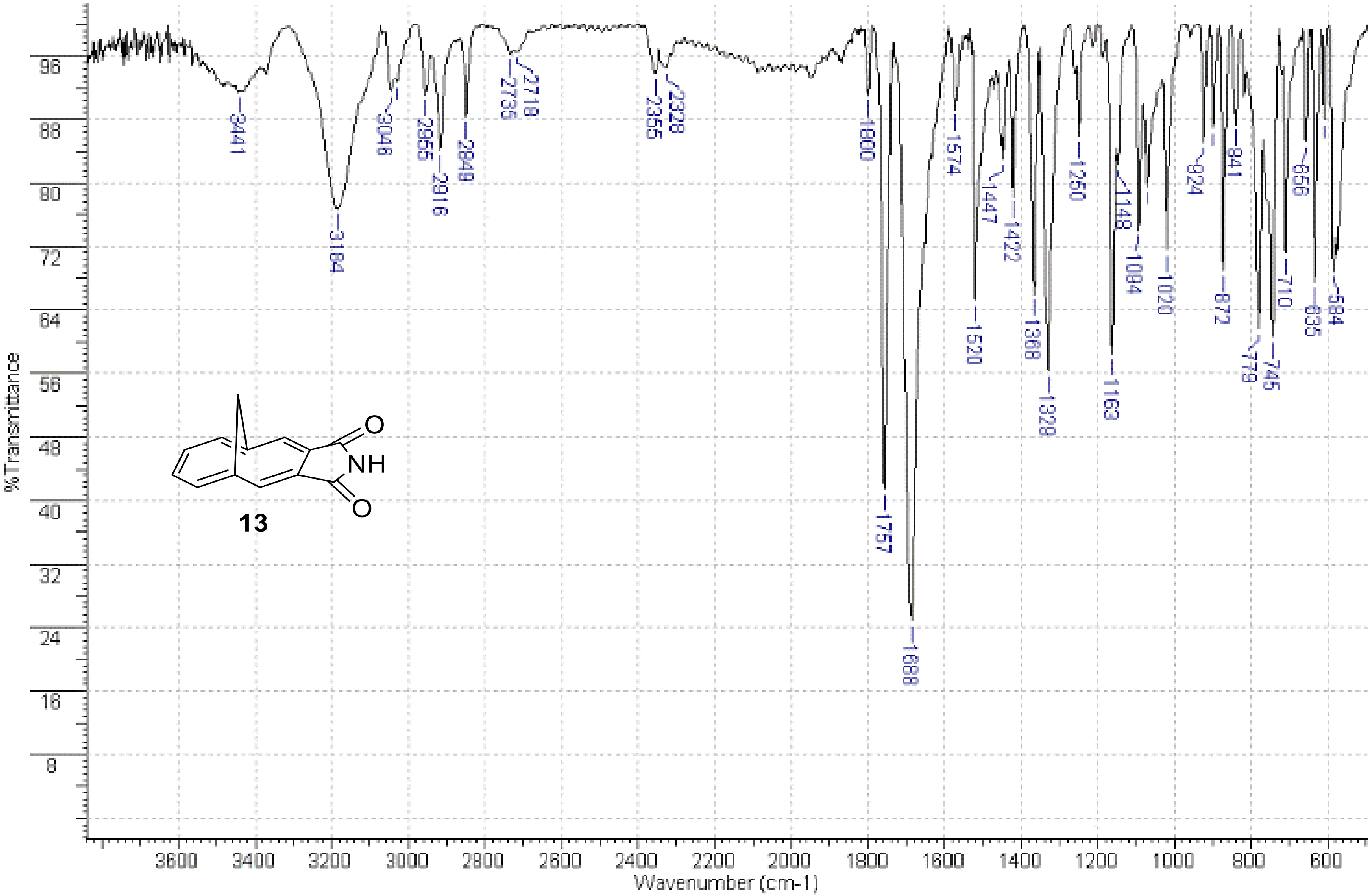

Figure S51. IR (KBr pellet)—compound **13**.

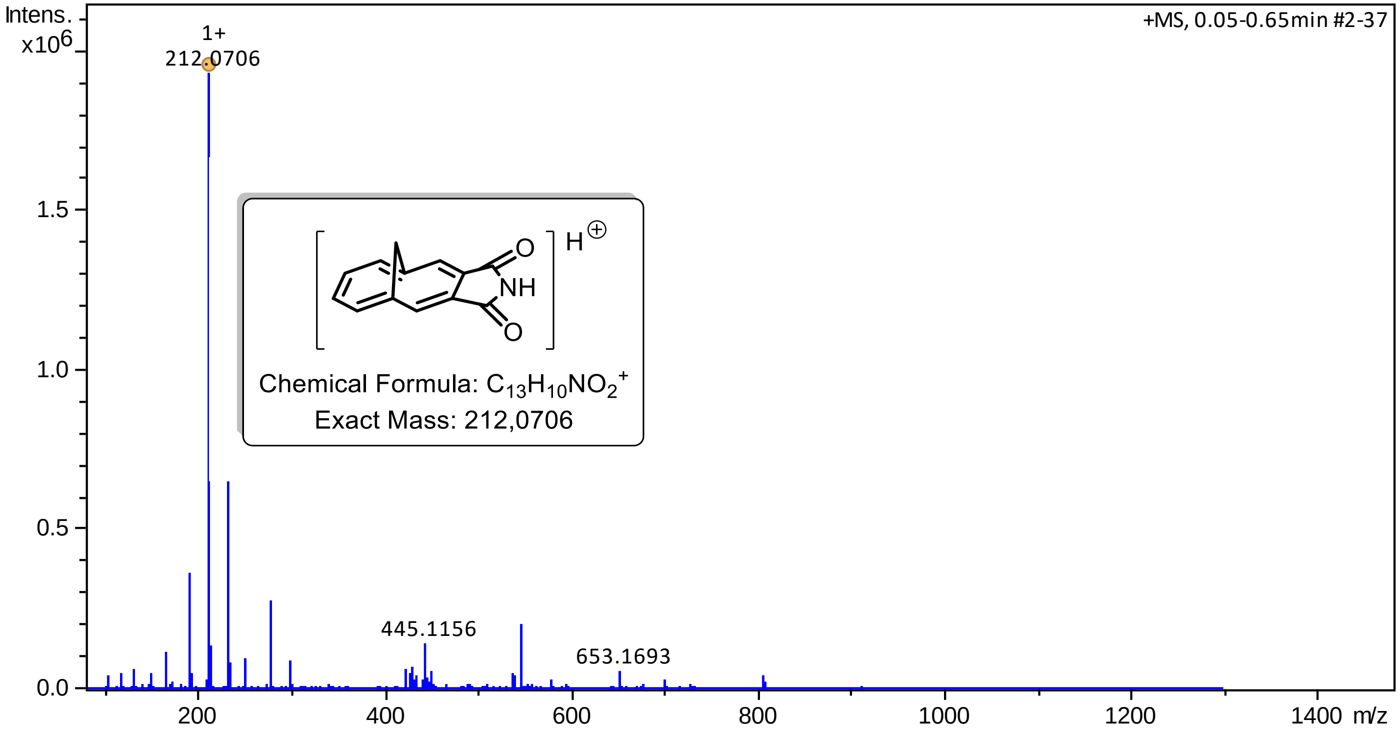

Figure S52. HRMS—ESI-TOF—compound **13**.

**Meas. *m/z***	**Ion Formula**	***m/z***	**Err [ppm]**	**mSigma**	**Score**	**rdb**	**Adduct**
212.0706	$C_{13}H_{10}NO_2$	212.0706	0.1	11.7	100	9.5	M+H

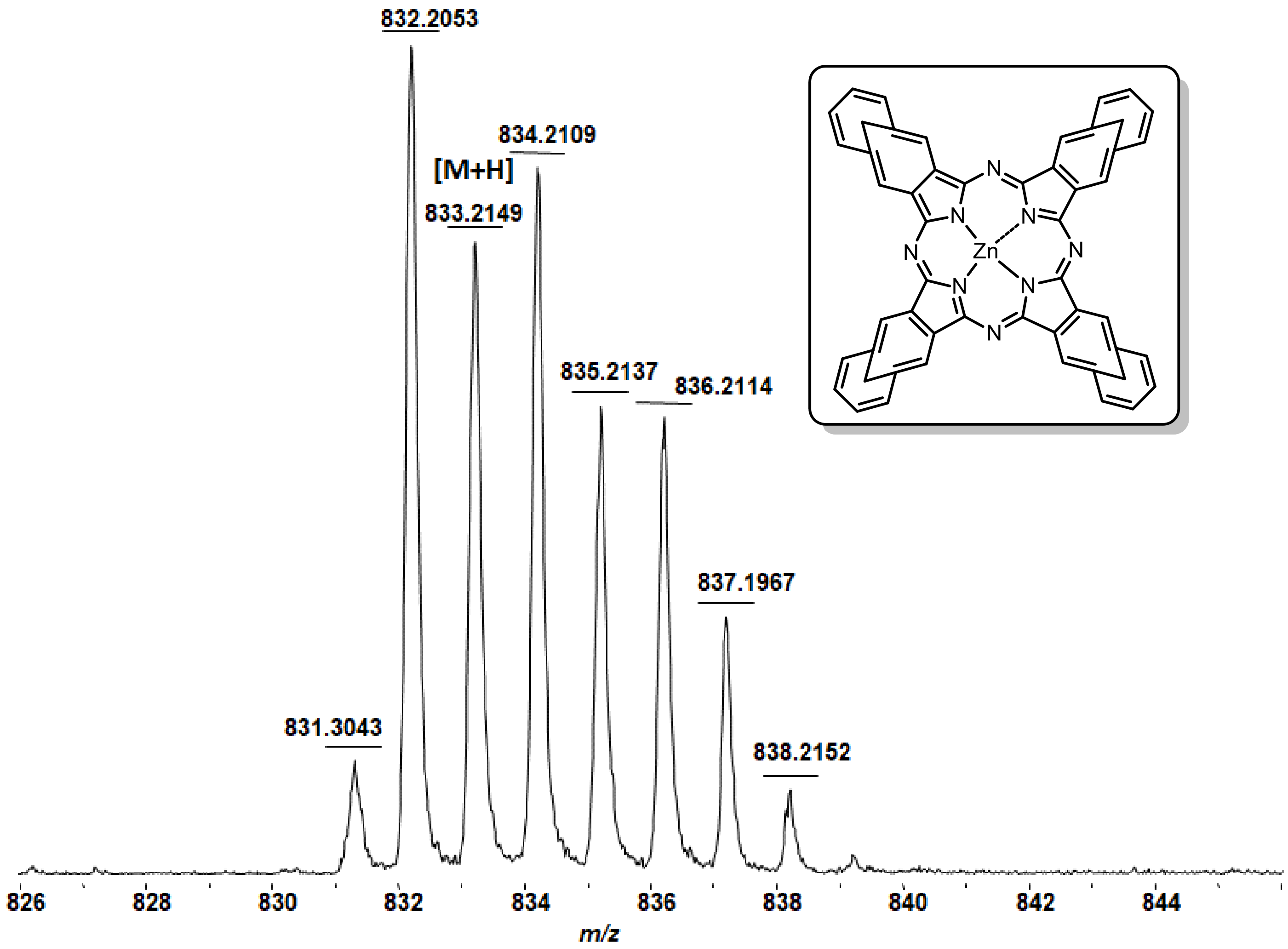

Figure S53. HRMS (MALDI-TOF) Annulenecyanine 10. Calc for $[M]^{\cdot+}$, $C_{52}H_{32}N_8Zn^+$, 832.2041, found: 832.2053.

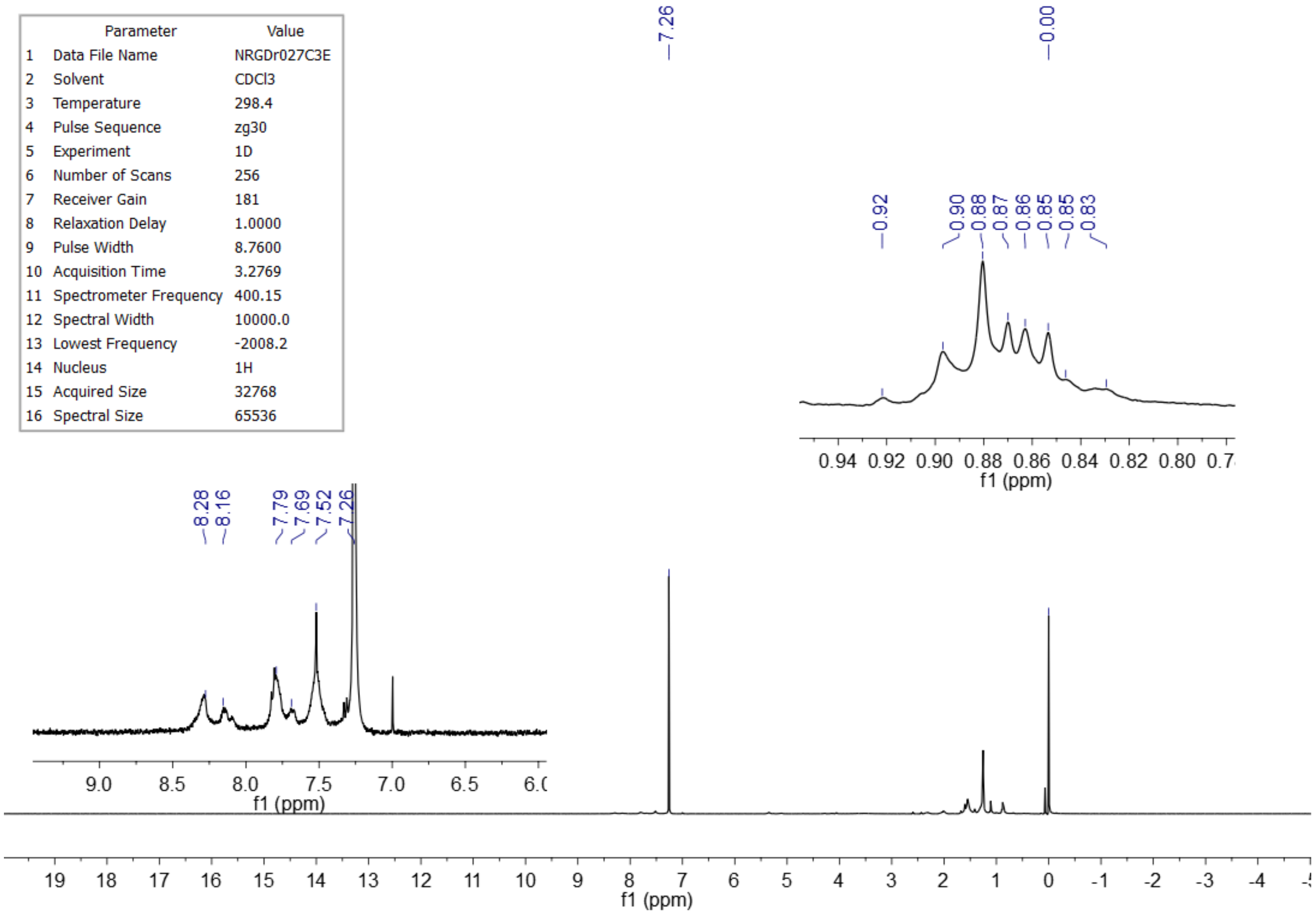

Figure S54. ^{1}H-NMR ($CDCl_3$, 400MHz) for **10** (diastereoisomeric mixture).

2.1. Fluorescence Quantum Yield Measurement of Compound 10 in DMF.

Fluorescence quantum yield (Φf) measurements were performed by a comparative method. Figure S55 show the emission spectrum of compounds **10** and the standard zinc naphthalocyanine (ZnNc). Excitation wavelength**:** 350 nm. The absorbance of both dyes was maintained at 0.1.

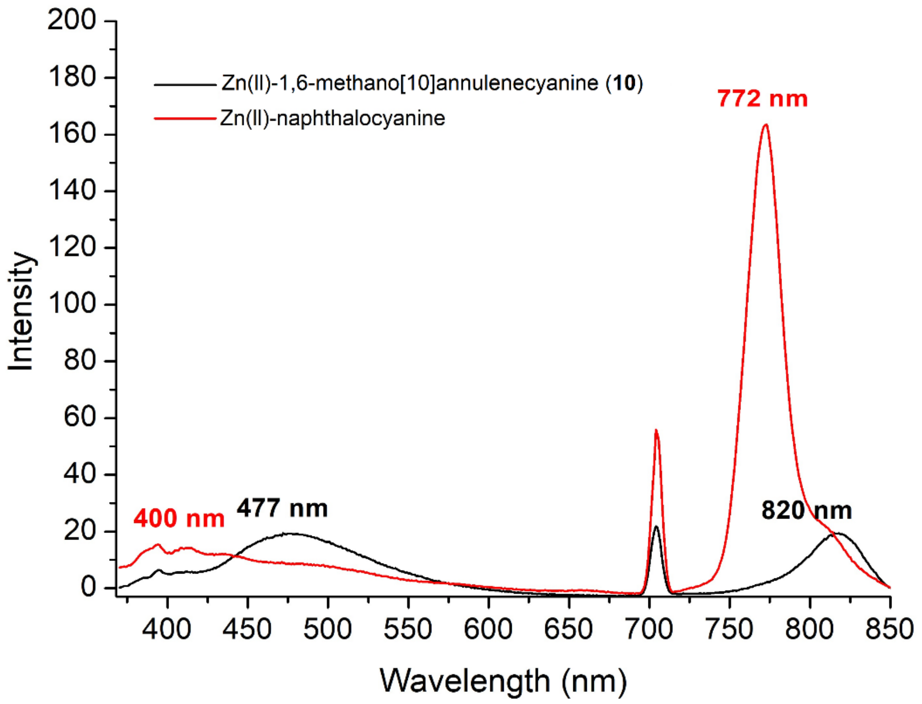

Figure S55. Emission spectra of compound **10** and standard **ZnNc** in DMF.

The fluorescence quantum yield Φ_F can be calculated by the expression below:

$$\Phi_F = \frac{\Phi_{std} F. A_{std}. n^2}{F_{std}. A. n_{std}^2} \tag{1}$$

Where:

- **Φ_{std}** = standard fluorescence quantum yield (ZnNc)[6,7].
- **F** and **F_{std}** = areas below emission curves of compound and standard, respectively.
- **A** and **A_{std}** = absorbances of compound and standard respectively.
- **n** and **n_{std}** = refractive index of solvents utilized for compound and standard.

The excitation wavelength was 350 nm. The Φ_F found for annulenocyanine **10** was 0.01 for the emission band at 820 nm [7,8].

2.2. Aggregation Study of Zn(II)-1,6-methano[10]annulenecyanine (10)

Different concentrations of compound **10** were prepared in DMF and the absorbance measured (Figure S56).

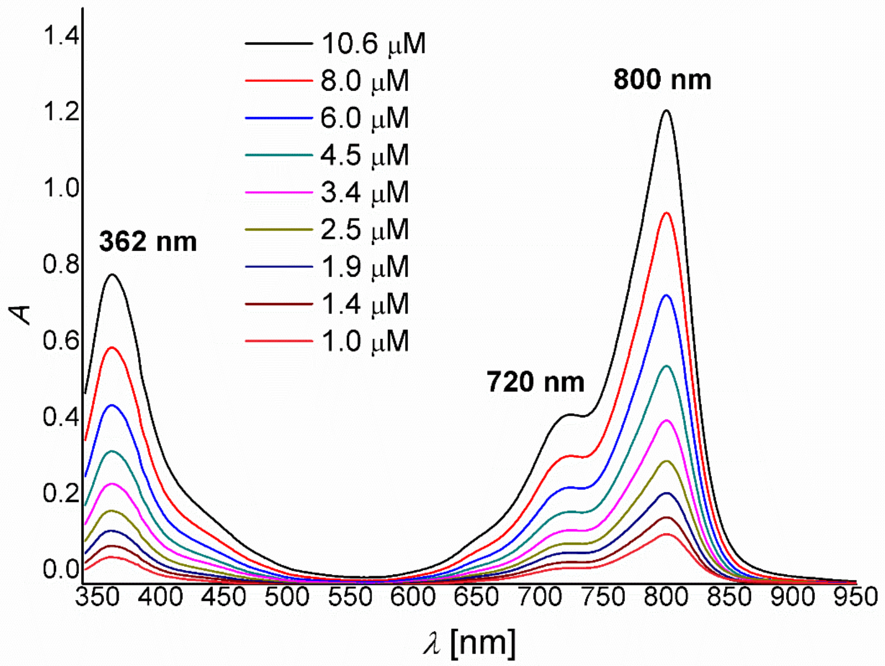

Figure S56. Aggregation behaviour of **10** in DMF at different concentrations.

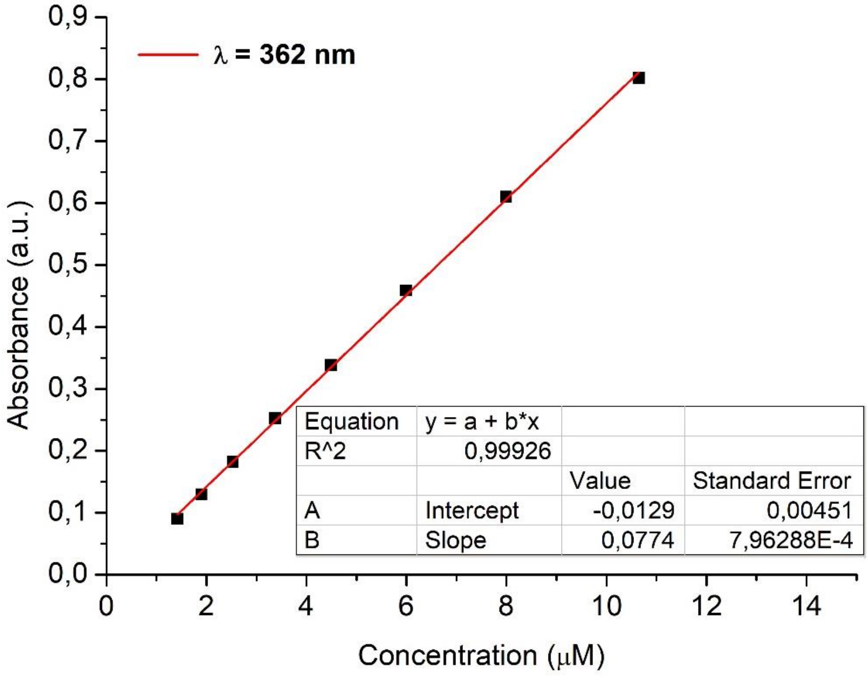

Figure S57. Linear fit equation for ε molar measure for **10** at 362 nm.

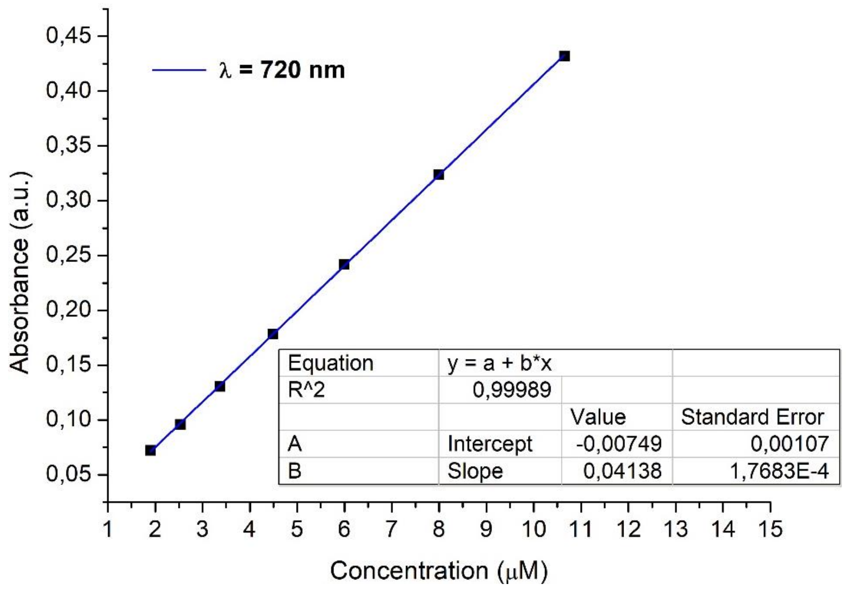

Figure S58. Linear fit equation for ε molar measure for **10** at 720 nm.

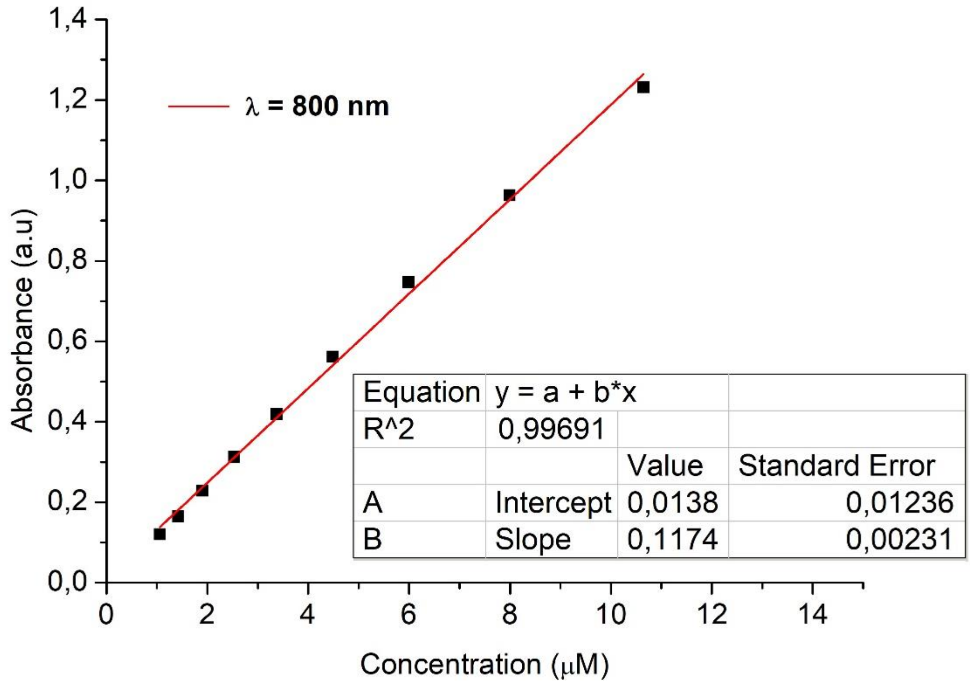

Figure S59. Linear fit equation for ε molar measure for **10** at 800 nm.

Through the linear regression graph, the value of ε can be obtained through the value of the slope of the line for each band being analyzed and obtained through equation (1). Table 1 below represents the logε values (as normally the value is represented) obtained for each absorption band.

Table 1. Molar extinction coefficient (Ɛ) for **10**.

**λ (nm)**	***362***	***720***	***800***
**Log(ε)**	*4.89*	*4.62*	*5.07*
**R^2**	*0.999*	*0.999*	*0.997*

References – Supplementary

1. Darms, R.; Threlfall, T.; Pesaro, M.; Eschenmoser, A. Cycloheptatrien-1, 6-dicarbonsäure und Bicyclo-[4, 1, 0]-2, 4-heptadien-1, 6-dicarbonsäureanhydrid. *Helvetica Chim. Acta* **1963**, *46*, 2893–2905, doi:10.1002/hlca.19630460746.
2. Iorga, B.I.; Ricard, L.; Savignac, P. Carbanionic displacement reactions at phosphorus. Part III. Cyanomethylphosphonate vs. cyanomethylenediphosphonate. Synthesis and solid-state structures. *J. Chem. Soc. Perkin Trans. 1* **2000**, *1*, 3311–3316, doi:10.1039/b003371p.
3. Sarmah, C.S. ; Kataky, J.C.S. A novel synthesis of substituted 1,6- methano[10]annulenes using a phase transfer catalyst. Indian *J. Chem. Sect. B Org. Chem. Incl. Med. Chem.* **1993**, *25*, 1149–1150.
4. Oda, M.; Nakamura, T.; Neha, M.; Miyawaki, D.; Ohta, A.; Kuroda, S.; Miyatake, R. A Short-Step Synthesis of 1,6-Methano[10]annulene-3,4-dicarboximides and Their Benzene-, Naphthalene-, and Thiophene-Annulated Compounds. *European J. Org. Chem.* **2014**, *2014*, 5976–5985.
5. Kuroda, S.; Oda, M.; Tamura, N.; Miyatake, R.; Matsumoto, N.; Horino, Y.; Miyawaki, D. Synthesis and Emission Properties of 1,6-Methano[10]annulene-3,4-dicarboximides. *HETEROCYCLES* **2011**, *83*, 789, doi:10.3987/com-11-12135.
6. Topal, S.Z.; Işci, Ümit; Kumru, U.; Atilla, D.; Gürek, A.G.; Hirel, C.; Durmuş, M.; Tommasino, J.-B.; Luneau, D.; Berber, S.; et al. Modulation of the electronic and spectroscopic properties of Zn(ii) phthalocyanines by their substitution pattern. *Dalton Trans.* **2014**, *43*, 6897, doi:10.1039/c3dt53410c.
7. Ogunsipe, A.; Chen, J.-Y.; Nyokong, T. Photophysical and photochemical studies of zinc(ii) phthalocyanine derivatives?effects of substituents and solvents. *New J. Chem.* **2004**, *28*, 822, doi:10.1039/b315319c.
8. Wheeler, B.L.; Nagasubramanian, G.; Bard, A.J.; Schechtman, L.A.; Kenney, M.E. A silicon phthalocyanine and a silicon naphthalocyanine: synthesis, electrochemistry, and electrogenerated chemiluminescence. *J. Am. Chem. Soc.* **1984**, *106*, 7404–7410, doi:10.1021/ja00336a019.
